# A Case-by-Case Evolutionary Analysis of Four Imprinted Retrogenes

**DOI:** 10.1111/j.1558-5646.2010.01213.x

**Published:** 2011-05

**Authors:** Ruth B McCole, Noeleen B Loughran, Mandeep Chahal, Luis P Fernandes, Roland G Roberts, Franca Fraternali, Mary J O'Connell, Rebecca J Oakey

**Affiliations:** 1 Department of Medical and Molecular Genetics, King's College LondonLondon SE1 9RT, United Kingdom; 2 E-mail: ruth.mccole@genetics.harvard.edu; 3 Bioinformatics and Molecular Evolution Group, School of Biotechnology, Faculty of Science and Health, Dublin City UniversityGlasnevin Dublin 9, Ireland; 4 Centre for Scientific Computing & Complex Systems modeling (SCI-SYM), Dublin City UniversityGlasnevin Dublin 9, Ireland; 5 E-mail: noeleen.loughran@gmail.com; 6 E-mail: mandeep.chahal@kcl.ac.uk; 7 Randall Division of Cell and Molecular Biophysics, King's College LondonLondon SE1 1UL, United Kingdom; 8 E-mail: lpfernandes@gmail.com; 9 E-mail: roli.roberts@genetics.kcl.ac.uk; 10 E-mail: franca.fraternali@kcl.ac.uk; 11 E-mail: mary.oconnell@dcu.ie; 12 E-mail: rebecca.oakey@kcl.ac.uk

**Keywords:** Epigenetics, gene expression, imprinting, molecular evolution, retrogene

## Abstract

Retroposition is a widespread phenomenon resulting in the generation of new genes that are initially related to a parent gene via very high coding sequence similarity. We examine the evolutionary fate of four retrogenes generated by such an event; mouse *Inpp5f_v2, Mcts2, Nap1l5*, and *U2af1-rs1*. These genes are all subject to the epigenetic phenomenon of parental imprinting. We first provide new data on the age of these retrogene insertions. Using codon-based models of sequence evolution, we show these retrogenes have diverse evolutionary trajectories, including divergence from the parent coding sequence under positive selection pressure, purifying selection pressure maintaining parent-retrogene similarity, and neutral evolution. Examination of the expression pattern of retrogenes shows an atypical, broad pattern across multiple tissues. Protein 3D structure modeling reveals that a positively selected residue in *U2af1-rs1*, not shared by its parent, may influence protein conformation. Our case-by-case analysis of the evolution of four imprinted retrogenes reveals that this interesting class of imprinted genes, while similar in regulation and sequence characteristics, follow very varied evolutionary paths.

Retrogenes are functional protein-coding genes derived from the RNA-mediated retroposition of a “parent” gene (Kaessmann et al. 2009). Because a retrogene is formed on the basis of the mature spliced mRNA of a parental gene, it will normally be mono-exonic, and hence is easily recognizable as a retroposed copy of its parent gene.

Retrogene generation in mammals depends upon the enzymatic machinery of LINE1 elements reverse transcribing endogenous mRNAs of “parent” genes and inserting the cDNA into the genome at a new location (Esnault et al. 2000; Wei et al. 2001). The products of retroposition events often have, in the past, been dismissed as nonfunctional. The term “retrogene” refers to a product of retroposition that generates a functional protein, and so direct evidence of protein function is formally required for a product of a retroposition event to be classified as a retrogene.

Retroposition creates an interesting evolutionary scenario for the genes involved. The new gene, in terms of the open reading frame, is often an exact copy of its parent (although retroposition can also result in truncation of the open reading frame). Retrogenes may also co-opt existing exons around them to form chimeric genes (Wang et al. 2006; Zhu et al. 2009). What impact has this retroposition process had on the evolution of these genes? Possibilities include neofunctionalization, (Ohno 1970), subfunctionalization (Force et al. 1999), and others, reviewed in Conant and Wolfe (2008).

Retroposition is akin to gene duplication although the evolutionary consequences in terms of rate variation have not been widely studied in the case of retrogenes. There is a conflict in the literature as regards the evolutionary rates of gene duplicates, certain studies conclude that pairs of duplicate genes evolve at different rates, with one gene per pair undergoing changes in function (Zhang et al. 2003; Kellis et al. 2004). Other analyses have concluded that gene duplication has no effect on the rate of evolution of either duplicate (Robinson-Rechavi and Laudet 2001). Additionally, it has been proposed that duplicate genes evolve faster than nonduplicates, but that there is no asymmetry in the rate of evolution between the two duplicates (Kondrashov et al. 2002).

In general, gene duplications involving the relocation of a gene duplicate to a new genomic context result in different evolutionary rates in those duplicate copies, specifically new duplicates evolve faster than their “parent” genes (Cusack and Wolfe 2007). Retroposition can create this precise scenario as broadly supported by analyses of retroposed genes (Gayral et al. 2007). Similarly, genomic context has been found to affect the rate of duplicate gene evolution, both in drosophila (Zhang and Kishino 2004a) and yeast (Zhang and Kishino 2004b). In summary, retrogenes seem to evolve faster than their parent genes, although this reflects broad trends rather than gene-by-gene studies. Here, we present a new case study of the evolution of four retrogenes with similar origins and genomic environments, together with their “parent” genes. We wished to determine if the similar mode of origination of these genes, their shared classification as imprinted, and their similar regulation have all contributed to a similar evolutionary rate variation in these genes.

We examine the evolution of the mouse retrogenes *Inpp5f_v2, Mcts2, Nap1l5*, and *U2af1-rs1*, or *Zrsr1*, together with their parent genes. All four retrogenes are subject to genomic imprinting (Nabetani et al. 1997; Smith et al. 2003; Choi et al. 2005; Wood et al. 2007). Although direct evidence for functional protein products has yet to be obtained, transcription of these genes occurs in a wide variety of tissues and each contains an intact open reading frame. Imprinted genes are epigenetically marked so that they are exclusively or predominantly expressed from the chromosome of a particular parental origin. The above four genes are exclusively paternally expressed. All four genes possess a CpG island promoter, which is differentially methylated in the gametes (unmethylated in sperm, methylated in oocytes) (Zhang et al. 2006; Wood et al. 2007). Each is located within the intron of a “host” gene (Table 1), and has a parent gene on the X chromosome (reviewed by McCole and Oakey 2008 and Wood and Oakey 2006). Understanding how imprinted genes evolved can inform on their function, which is an important facet of understanding imprinting in general.

**Table 1:** Information on the retrogenes studied, their “parent” and “host” genes

Imprinted retrogene	Accession number	Parent gene	Parent gene accession number	Position of retrogene in mouse genome build mm9	Host gene	Host intron size (bp)
*Inpp5f_v2*	DQ648020	*Vma21 (or Tmem114a)*	BC028317	*chr7:135,832,012–135,832,332*	*Inpp5f*	5,329
*Mcts2*	NM_025543	*Mcts1 (or Mct1)*	NM_026902	*chr2:152,512,884–152,513,678*	*H13*	2,480
*Nap1l5*	NM_021432	*Nap1l2*	NM_008671	*chr6:58,855,227–58,857,120*	*Herc3*	21,751
		*Nap1l3*	NM_138742			
*U2af1-rs1 (or Zrsr1)*	NM_011663	*U2af1-rs2 (or Zrsr2)*	NM_178754	*chr11:22,872,029–22,874,908*	*Commd1 (or Murr1)*	25,337

The parent genes for mouse *Inpp5f_v2, Mcts2* and *U2af1-rs1* have been identified unambiguously as *Tmem114a, Mcts1* (Wood et al. 2007) and *U2af1-rs2* or *Zrsr2*, (Smith et al. 2003) respectively. We retain the “U2af1-rs” nomenclature here because of the confusing presence of *ZRSR1* in the human genome. Human *ZRSR1* is likely to have been formed by an independent retroposition event of the ancestral U2af1-rs2, because it is located in a different genomic position compared to mouse *U2af1-rs1* (Zhang et al. 2006; Wood et al. 2007). It is possible to date the retroposition event that lead to these retrogenes being generated, by examining the host gene intron for presence of a retrogene ortholog, as in Wood et al. (2007). We have extended this analysis further here using more recent sequence data from a wider variety of species.

*Inpp5f_v2* is derived from *Tmem114a*. Recently it has emerged that *Tmem114a* is the murine homolog of human *VMA21*, an essential assembly chaperone for the V-ATPase complex, which is the main mammalian proton pump (Ramachandran et al. 2009). We will henceforth refer to *Tmem114a* as *Vma21*

The parentage of *Nap1l5* is less clear. Two paralogues, *Nap1l2* and *Nap1l3*, exist on the X chromosome. Both are mono-exonic, and are likely to be retrogenes derived from one of the multiexonic genes *Nap1l4* or *Nap1l1*. Previously, *Nap1l2* was identified as the most likely parent for *Nap1l5* through phylogenetic reconstruction (Wood et al. 2007). Both *Nap1l2* and *Nap1l3* have been examined here as putative parents. For information on the gene families and nomenclature used in this study see Table 1.

To examine the evolution of these four parent-imprinted retrogene families at the protein level, we have used codon-based models of evolution in a maximum likelihood framework to test for heterogeneity in selective pressures across parent and retrogenes (Yang 1997; Yang and Nielsen 2002; Zhang et al. 2005). We note that results using computational analyses alone can be misinformative when not examined in the context of the underlying biology of the proteins concerned (Hughes 2007, 2008). Here, we have suggested biological reasons for the selection pressures predicted, and treat the results as a tool for generating hypotheses on the function of the proteins in question, to be tested empirically.

Positive selective pressure resulting in amino acid substitutions has been definitively linked to changes in protein function (Levasseur et al. 2006). Hence, prediction of positively selected amino acids gives an important insight into potential functional changes in the proteins coded for in this study. Positive selection in the retrogene lineage alone could indicate neofunctionalization. We were able to differentiate between the parent genes and retrogenes by identifying the emergence of each retrogene individually using phylogenetics. Then using site-specific, lineage-specific, and combined site and lineage-specific models of codon evolution, we examined the evolutionary rate heterogeneity of these proteins.

## Materials and Methods

### SEQUENCE RETRIEVAL AND INVESTIGATION OF RETROGENE AGE

To identify retrogene orthologs from as many species as possible, the mouse retrogene was compared in a sequence similarity search to the host gene intron in the species of interest using blat (Kent 2002). For the following species, this was done using the UCSC genome browser, (UCSC). Chimp (*Pan troglodytes)* = panTro2, orangutan (*Pongo pygmaeus abelii)* = ponAbe2, rhesus (*Macaca mulatta*) = rheMac2, marmoset (*Callithrix jacchus*) = calJac1, rat (*Rattus norvegicus*) = rn4, guinea pig (*Cavia porcellus*) = cavPor3, cat (*Felis catus*) = felCat3, dog (*Canis lupus familiaris*) = canFam2, horse (*Equus caballus*) = equCab2, cow (*Bos taurus*) = bosTau4, opossum (*Monodelphis domestica*) = monDom5, platypus (*Ornithorhynchus anatinus*) = ornAna1, and chicken (*Gallus gallus*) = galGal3.

For the sloth (*Choloepus hoffmanni*) and the lesser hedgehog tenrec (*Echinops telfairi*), chained alignments are not available. In the ensembl genome browser, gene scaffolds were identified containing the host gene and were then compared using blat to the mouse genome to identify a retrogene ortholog. For the elephant, *Loxodonta africana*, armadillo, *Dasypus novemcinctus*, and the opossum species, *Monodelphis domesticus*, the gene in question was located in the human genome, and then identified in the species of interest using the pre-existing BLASTZ alignments from the Ensembl website (Ensembl). BLASTN searches of the NCBI trace archive (NCBITraceArchive), or the dbEST database (dbEST) in the case of opossum (*Trichosurus vulpecula*) was used when data were not available in Ensembl.

To identify parent gene orthologs, the UCSC chained alignments and Ensembl BLASTZ alignments were used, together with comparisons using blat (Kent 2002).

Homologous sequence alignments were checked for quality by eye and manually edited as necessary. See Supplementary File 1 for a full list of the nucleotide sequences used.

### PCR EXPRESSION STUDIES

RNA was extracted from frozen C57BL-6 tissues using the Qiagen (Crawley, UK) RNeasy Mini kit (cat. no. 74104) according to the manufacturer's instructions. RNA was quantified on an Agilent (Wokingham, UK) Bioanalyzer and 5 μg was used for each cDNA synthesis reaction. Invitrogen (Paisley, UK) SuperScript First-Strand kit (cat. no. 12371-019) was used to generate cDNA, according to the manufacturer's instructions. cDNA was diluted 1 in 4 for PCR. PCR was carried out with 1 μl diluted cDNA template, 0.5 μl 20 μM primer (Supplementary File 2), and 1.1X Abgene (Epsom, UK) ReddyMix PCR Master Mix (cat. no. ab-0575/LD/B), using 28 PCR cycles at an annealing temperature of 60°C. PCRs were visualized with UV light on 1% agarose gel stained with ethidium bromide. Primers used are listed in Supplementary File 2. To ensure primers were amplifying only the required gene (no cross-contamination), bands were sequenced using Applied Biosystems (California, USA) BigDye Terminator version 3.1 Cycle Sequencing kit.

### MULTIPLE SEQUENCE ALIGNMENT (MSA) AND PHYLOGENETIC RECONSTRUCTION

Protein-coding sequences were translated using in-house software and aligned using ClustalW (Thompson et al. 1994). Alignments were inspected using Se-Al (Rambaut 1996) and JalView 12.2.0 (Clamp et al. 2004).

Phylogenetic reconstruction was carried out using MrBayes 3.1.2 (Ronquist and Huelsenbeck 2003). The model for amino acid substitution to be used, JTT + G for all MSAs, was determined using ModelGenerator 0.85 (Jones et al. 1992).

### SHIMODAIRA–HASEGAWA (SH) TEST

As the gene phylogenies did not map precisely onto the pruned species phylogeny for each MSA, we performed the SH test to determine if these phylogenies were significantly different. The pruned species phylogeny was generated by simply removing taxa from the canonical species phylogeny as resolved by Murphy et al. (2001). This comparison was carried out using the SH test (Shimodaira and Hasegawa 2001) implemented in the TreePuzzle 5.2 (Schmidt et al. 2002). The results are given in Supplementary File 3. There is no significant difference between the topologies for the Inpp5f_v2-Vam21, Nap1l, and U2af1-rs families. In the Mcts family, the gene tree was a better fit to the data and so this was used for all further analysis.

### MODELS OF CODON EVOLUTION

We estimated ω across the four alignments individually using both site-specific and lineage-specific models implemented in PAML version 4.2a, these models are described in Yang (1997), Yang and Nielsen (2002), and updated in Zhang et al. (2005). ω is an estimate of the ratio of nonsynonymous to synonymous substitutions at each site in the MSA, normalized by the number of possible substitutions of each type. ω provides an estimate at each codon of the type of selection pressure (positive, neutral or purifying) that has occurred during evolution.

Nine different models of codon evolution were tested, along with two “null” models that are essential for statistical validation. For full descriptions of all models used in this study and parameters therein please see (Yang 1997; Yang and Nielsen 2002; Zhang et al. 2005). A brief description is given in Supplementary File 4. For each gene family, the model that fit the data best following statistical tests was chosen. In some cases, this was a site-specific model that provided estimates on evolutionary rates in specific regions of the protein. In other cases, a lineage-specific model was chosen. When a lineage-specific model fits the data best, this indicates asymmetry in evolutionary rates between the phylogenetic branches in question, or between parent gene and retrogene lineages as is the case in this study.

In all four gene families, either Model 3 (*K* = 2) or Model B was the best fit to the data. Model 3 (*K* = 2) is a site-specific model, where each site is only allowed one of two values of ω. No constraint is placed on the value of ω, which can be larger than 1, so positively selected sites are allowed. If Model 3 (*K* = 2) is the best fit to the data, there is no evidence that the foreground (retrogene lineage) has evolved differently from the background (parent gene lineage). Model B is the lineage-specific extension of Model 3 (*K* = 2). Sites are allowed to have different values of ω simultaneously, so the foreground lineage can be shown to have evolved differently from the background. Four possible values of ω are allowed, which can be greater than 1 (positive selection). A summary of all models tested is in Supplementary File 4.

### PROTEIN THREE-DIMENSIONAL (3D) STRUCTURE MODELLING

The modeled structures of the U2af1-rs proteins were obtained by homology modeling from the crystal structure of the U2AF35 central domain (chain A, pdb code = 1JMT) (Kielkopf et al. 2001).

The sequence alignment used to build the models was generated with the program PRALINE with the homology-extended alignment strategy (Simossis et al. 2005). Three-dimensional models were generated using the MODELLER package (Marti-Renom et al. 2000). The selected model was chosen on the basis of the MODELLER objective function's score. Images were produced with visual molecular dynamics (VMD) 1.8.5 software (Humphrey et al. 1996).

The VSL2 package was used for disorder prediction (Peng et al. 2006). The software provides a disorder probability for each residue. To achieve the most accurate results, we have used VSL2 with four features sets; amino acid composition, two independent secondary structure predictions, and PSI-BLAST profiles as described in Peng et al. (2006).

## Results

### IMPRINTED RETROGENES HAVE BEEN ACCURATELY DATED

**Figure 1:**
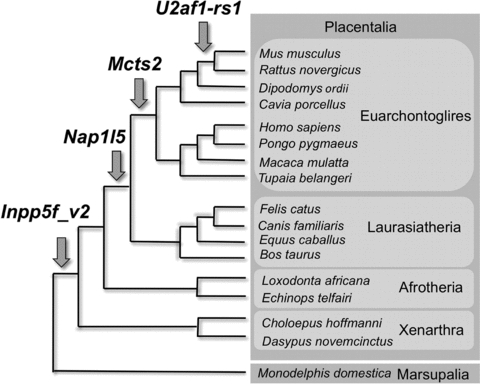
Timing of retroposition events within mammalian evolution. Arrows indicate the time of insertion of the retrogene indicated, given the sequence data available to date.

We have previously shown estimates of the ages of the four retrogene insertions in question (Wood et al. 2007). We have been able to refine these estimations for *Inpp5f_v2, Nap1l5*, and *U2af1-rs1* orthologs (Fig. 1). *Inpp5f_v2* orthologs were known not to be present in the opossum (Wood et al. 2007), but we have found orthologs in the elephant and armadillo (Supplementary File 5). Retroposition of *Inpp5f_v2* must have occurred after the Marsupalia/Placentalia split, but before the split of the Xenarthra and Afrotheria.

The retroposition event that formed *Nap1l5* occurred after Xenarthra and Afrotheria clades diverged, but before the Laurasiatheria/Euarchontoglires divergence. We were unable to refine the time of emergence of *Mcts2* compared with information in Wood et al. (2007).

*U2af1-rs1* was formed from the most recent retroposition event of the four. We have examined the relevant intron of the host gene *Commd1* in the kangaroo rat (*Dipodomys ordii*) and guinea pig (*C. porcellus*) and found no similarity with mouse *U2af1-rs1* We also searched the deer mouse (*Peromyscus maniculatus*) for sequences with similarity to mouse *U2af1-rs1*. We discovered an incomplete sequence, but with no flanking host exons it is impossible to verify as an *U2af1-rs1* ortholog. Thus at this time the *U2af1-rs1* retroposition event is confined to mouse and rat, and may in future be confirmed in the deer mouse.

### EXPRESSION OF ALL GENES IS WIDESPREAD AND IS ROUGHLY SIMILAR WITHIN FAMILIES

**Figure 2:**
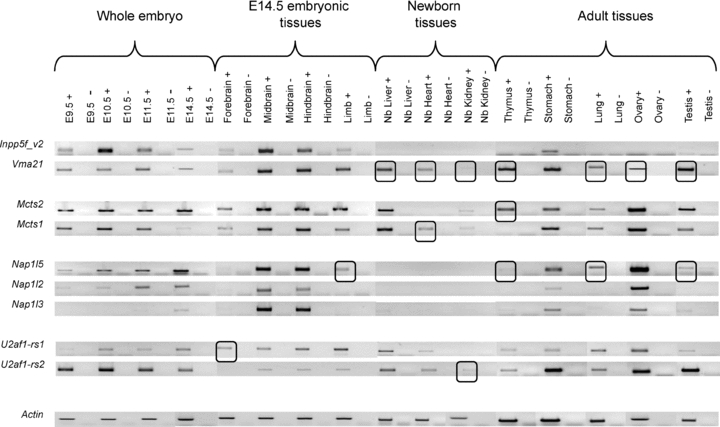
Expression of the retrogene and parent gene transcripts in multiple tissues and developmental stages. Expression was assayed by reverse-transcriptase PCR with Actin as a control for each different tissue sample (bottom row). Tissues where only one member of a gene family is expressed are highlighted with black squares.

We were interested in whether the retrogenes might be different from their parents in terms of their expression patterns. We discovered that all the genes in question are expressed in numerous tissues and at many stages of embryonic development in the mouse (Fig. 2). There are specific tissues where only one member of a gene family is expressed. These are highlighted in Figure 2. *Vma21* is ubiquitously expressed (although the expression in newborn kidney is difficult to see but present at low levels). This is expected as it is an essential assembly chaperone, thought to be required in all mammalian cells (Ramachandran et al. 2009). Although the retrogenes all have a methylated promoter in oocytes and an unmethylated promoter in sperm, this does not necessarily correlate with their expression in ovary and testis, which contain somatic tissues as well as germ cells. Indeed, transcription through the retrogene locus in oocytes could be required for methylation establishment (Chotalia et al. 2009).

### MAJOR CHANGES IN THE NAP1L GENE FAMILY

We performed an MSA of the amino acid sequences for the Nap1l family across 11 mammalian species. We focused on the three retrogenes *Nap1l5, Nap1l2*, and *Nap1l3*. The multiexonic genes *Nap1l1* and *Nap1l4* sequences were also included for some species (Supplementary File 6). *Nap1l5* orthologs are the youngest family members, as they lack the region of homology shared by all other family members at residue 432 to 540 in the alignment. The alignment shows that large changes have taken place since the retroposition events that produced *Nap1l2, 3* and *5* orthologs. For example, *Nap1l5* orthologs are truncated compared to *Nap1l2* and *Nap1l3*, mouse *Nap1l5* having 158 amino acids compared with 546 amino acids for mouse *Nap1l3* and 462 for mouse *Nap1l2*. The *Nap1l3* orthologs have a protein region composed almost entirely of serine residues from residue 38 to 82 of the alignment that is unique among the Nap1l family. The Nap1l gene family members have undergone major structural changes during or after the duplication events that produced the gene family members, and these likely impacted protein function.

### PHYLOGENY RECONSTRUCTION SUPPORTS THE SEPARATION OF RETROGENES AND PARENT GENES INTO DISTINCT LINEAGES

**Figure 3:**
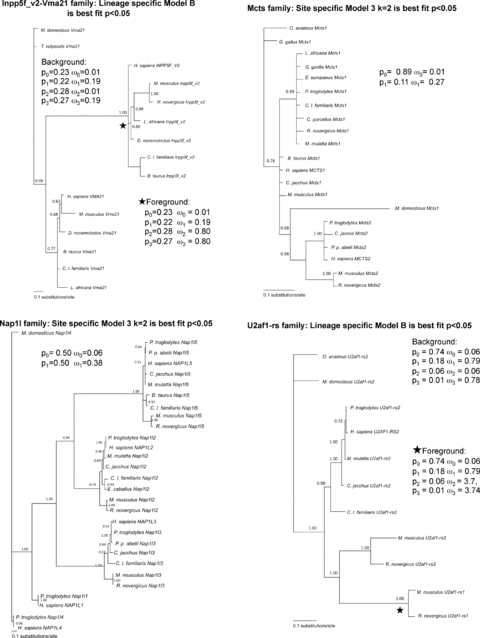
Phylogenetic reconstruction for each gene family. Posterior probabilities for each branch are shown. ω values for the best-fit site-specific model (Mcts and Nap1l families) and foreground and background for best-fit lineage-specific model (Inpp5f_v2-Vma21 and U2af1-rs families) are shown.

For every gene family, the parent genes and retrogenes were separated into distinct lineages with high posterior probabilities (Fig. 3). The Nap1l family phylogeny is in agreement with previously published data (Wood et al. 2007), with *Nap1l2* predicted to be the closest relative to *Nap1l5*.

Within each lineage (parent gene or retrogene) the correct species phylogeny is not always preserved. It is not unusual for a gene tree to be discordant with the species tree, and these have previously been used for codon evolution analysis ([Bibr b58][Bibr b50]; Sassi et al. 2007; Chapman et al. 2008). Indeed, there may be systematic reasons for gene tree–species tree discordance. Other factors apart from the ancestry of the gene sequences in question, can have an impact on topology such as the presence of strong negative or positive selection (Massey et al. 2008). For each of the four datasets, we have also constructed a pruned canonical species phylogeny. These species phylogenies were compared to the gene phylogenies using a statistical framework implemented in the SH test (Shimodaira and Hasegawa 2001). The results of the comparisons are given in Supplementary File 3.

In summary, the gene and species phylogenies did not vary significantly, for all gene families but Mcts. Following SH test analysis of the Mcts gene tree and species tree, the gene tree was a statistically better fit to the data, and so all further analyses for Mcts were carried out using the gene tree. For the remaining three gene families, Nap1l, U2af1-rs, and Inpp5f_v2-Vma21, we have applied the species phylogenies to the codon evolution analyses. The results from the codon evolution analyses did not differ significantly based on the gene or species phylogeny, see Supplementary File 7. This is consistent with the results from the SH test where there was no significant difference between the gene and species trees for Nap1l, U2afs, and Inpp5f. The results described below for codon evolution analyses are from the inferred gene family phylogenies for all genes.

### EACH GENE FAMILY HAS EVOLVED DIFFERENTLY

We investigated the evolution of the coding DNA sequence of each gene family. For lineage-specific models, the imprinted retrogene lineages were each in turn designated as foreground, as shown by the positions of the stars in Figure 3.

Table 2 shows the models determined to fit the data best following LRT analysis for each gene. See Supplementary File 8 for full codon evolution results for each gene family from the gene tree analyses. What follows is a brief synopsis of these results on a gene-by-gene basis.

**Table 2:** Summary of codon evolution model that fits each gene family best following likelihood ratio test analysis

Model^1^	Estimates of parameters	Chi-squared test result	Positively selected sites^2^
Inpp5f_v2-Vma21 family			
Site specific model: Model 3: Discrete(K=2)	p_0_=0.49, p_1_=0.51 ω_0_=0.02, ω_1_=0.19	M0 v M3k2 2(2039.75–2024.97)=29.56* Critical value≥5.99	No positive selection
Lineage specific model: Model B	p_0_=0.23, p_1_=0.22, p_2_=0.28, p_3_=0.27 Parent Lineages (Background): ω_0_=0.01, ω_1_=0.19, ω_2_=0.15, ω_3_=0.19	M3k2 v Model B 2(2024.97–2020.32)=9.3* Critical value≥5.99	No positive selection
	Retrogene Lineages (Foreground): ω_0_=0.01, ω_1_=0.19, ω_2_=0.80, ω_3_=0.80		
Mcts family			
Site specific model: Model 3: Discrete(K=2)	p_0_=0.89, p_1_=0.11 ω_0_=0.01, ω_1_=0.27	M0 v M3k2 2(2554.31–2528.13)=52.36* Critical value≥5.99	No positive selection
Nap1l family			
Site specific model: Model 3: Discrete(K=2)	p_0_=0.50, p_1_=0.50 ω_0_=0.06, ω_1_=0.38	M0 v M3k2 2(12832.55–12667.28)=330.54* Critical value≥5.99	No positive selection
U2af1-rs family			
Site specific model: Model 3: Discrete(K=3)	p_0_=0.58, p_1_=0.31, p_2_=0.10 ω_0_=0.03, ω_1_=0.24, ω_2_=1.30	M3k2 v M3k3 1(7302.16–7293.06)=9.1* Critical value≥1.00	36 sites, p.p.>0.5 15 sites, p.p.>0.95 4 sites, p.p>0.99
Lineage specific model: Model B	p_0_=0.74, p_1_=0.18, p_2_=0.06, p_3_=0.01 Parent gene lineages (Background): ω_0_=0.06, ω_1_=0.79, ω_2_=0.06, ω_3_=0.78	M3k2 v Model B 2(7302.16–7284.63)=107.06* Critical value≥5.99	Foreground: 10 sites, p.p.>0.5 2 sites, p.p.>0.95 3 sites, p.p.>0.99
	Retrogene lineages (Foreground): ω_0_=0.06, ω_1_=0.79, ω_2_=3.74, ω_3_=3.74		

1 Model 3 categorizes each site in the alignment into either two (*K* =2) or three (*K* =3) categories of ω, the values for ω are estimated based on the data. The proportion of sites with these ω values is given as “p” with the corresponding subscript for the ω value. Model B allows a specific branch of the phylogenetic tree to be marked as foreground and categorizes the sites into four proportions, p_0, 1, 2_, and _3,_ with four different values of ω estimated for the foreground and background independently.

2 Where models predicted categories of sites with ω>1, indicating positive selection, the estimated numbers of sites with posterior probabilities >0.5, >0.95, and >0.99 of belonging to this category are listed. Codons are estimated as belonging to the category of positively selected using Naïve Empirical Bayes analysis only if Bayes Empirical Bayes is not available.

For the Inpp5f_v2-Vma21 gene family, the best site-specific model was Model 3 (*K* = 2) with all codons in the MSA under purifying selection (ω < 0.5). The lineage-specific model that fits the data significantly better (*P* < 0.05) than this is Model B, which allows differences in the retrogene (foreground) compared with the parent gene (background) lineages. Model B indicates that 23% of the sites have a predicted ω of 0.01 (purifying selection), 22% have ω predicted at 0.19 (slightly more relaxed purifying selection). This means that approximately 45% of the alignment is under purifying selection, regardless of lineage. A further 28% of the sites have ω predicted at 0.15 in the parent gene lineages and 0.80 in the retrogene lineage, and the remaining 27% have ω at 0.19 in the parent gene and 0.80 in the retrogene lineages. So, at 55% of the sites, the *Vma21* parent lineage is under purifying selection, whereas the *Inpp5f_v2* lineage is evolving neutrally (ω close to 1) at these sites. This means that natural selection is acting to preserve the protein sequence in the parent gene lineage, but 55% of the retrogene lineage codons are unconstrained. Of course, it is possible that the value of 0.80 may represent a signal for purifying selection averaged together with one for positive selection. In our analyses, we have tried to account for this in so far as current models permit, by using models that allow for both site- and lineage-specific evolution simultaneously.

For the Mcts family, lineage-specific models were not a significantly better fit to the data than site-specific models. Although the genes are still resolved into distinct lineages by the phylogenetic reconstruction, there was no evidence to support adaptive evolution of the *Mcts2* gene. The best model was determined to be Model 3 with two discrete ω values (*K* = 2). Overall this model predicts purifying selective pressure on the Mcts family with 89% of the codons estimated to have ω= 0.01, and 11% of the codons with ω= 0.27. All Mcts gene family members are under purifying selection. There is a strong evolutionary pressure on all these gene family members to retain the same amino acid sequence.

The Nap1l family shows similarity, in terms of selective pressures, to the Mcts family, although purifying selection is generally less strict in this case. Like the Mcts family, the lineage-specific models of evolution do not fit the data significantly better than the site-specific models. Again this indicates that differences in codon evolution between the *Nap1l5* retrogene lineage and the putative parent genes *Nap1l3* and *Nap1l2* were not detected by codon evolution analysis. The best site-specific model is Model 3 (K = 2) with 50% of the codons in the Nap1l family under stringent purifying selection with ω= 0.06, and the other 50% under slightly less-stringent purifying selection with ω= 0.38. Taking the codon evolution results alone, the Nap1l family would seem to have evolved in a similar way to the Mcts family. However, the MSA for the Nap1l family shows major changes in the open reading frames of the various family members as the family grew due to multiple transposition events. (See Supplementary File 6 for alignments). These changes could very well have profoundly affected protein function, although further examinations of the Nap1l protein structure and function are needed to verify this.

Interestingly, the U2af1-rs gene family does show evidence of positive selection (ω= 3.74). Model 3 (*K* = 3) is the best site-specific model. This model detects positive selection across 10% of sites, but these may only be positively selected in a particular lineage, and a site-specific model cannot address this, also site-specific models in general do not fit these data well. Using lineage-specific models, we have investigated this possibility and have found that Model B fits the data significantly better (*P* < 0.05). According to this model, 74% of the sites are evolving under purifying selection with ω= 0.06, and 18% of the sites have ω= 0.79, regardless of the lineage. We found that 6% of sites are predicted to be under purifying selection in the parent gene *U2af1-rs2* lineages (ω= 0.06), whereas these exact sites are under positive selection in the retrogene *U2af1-rs1* lineage (ω= 3.74). A further 1% of sites were evolving neutrally (ω= 0.78) in the parent gene *U2af1-rs2* lineages, but showed positive selection in the retrogene *U2af1-rs1* lineages (ω= 3.74). In summary, after the retroposition event that created ancestral *U2af1-rs1* from its parent gene, certain amino acids in the *U2af1-rs1* protein have been under evolutionary pressure to change (i.e., adaptive Darwinian selection), whereas the corresponding codons in the parent lineage are either under purifying selection or are evolving neutrally. This is suggestive of neofunctionalization unique to the retrogene *U2af1-rs1* lineage following the retroposition event. These pressures are absent from its parent gene lineage.

There were a total of 15 positively selected amino acid changes in the U2af1-rs1 retrogene lineage compared (Table 3). Very similar results were obtained when the pruned species phylogeny is used (Supplementary File 9). As the retrogene and parent proteins are dissimilar at their extreme C terminus, it is not surprising that examination of the U2af1-rs family protein alignment (Supplementary File 6) revealed that the last four positively selected residues (numbers 480, 485, 491, and 493 in the MSA) are in a poorly aligned region, are likely to be false positives and were subsequently disregarded. Apart from this region, all positively selected residues fall into regions of the alignment with high conservation between the different proteins, indicating a functional importance for these regions. We have examined the sites under positive selection for the U2af1 protein using both gene and species phylogenies. The sites estimated using both topologies were similar, see Supplementary File 9. We examined the sites from the gene phylogeny in further detail at the 3D structure level.

**Table 3:** Positions of positively selected codons in the U2af1-rs1 retrogene lineage

Position in alignment^1^	Amino acid in retrogene lineage	Amino acid in parent lineage	*P* value^2^ in parent lineage	Position in retrogene protein *U2af1-rs1* (*M. musculus*)	Position in parent protein *U2af1-rs2* (*M. musculus*)
38	M	L	0.659	33	38
46	A	L	0.57	41	46
63	L	E	0.996	55	62
154	E	G	0.576	142	154
206	V	I	0.745	192	206
313	V	M	0.678	300	313
355	P	D	0.997	342	355
361	S	F	0.501	348	361
	Y(mouse)				
384	H(rat)	R	0.875	371	384
385	H	R	0.965	372	385
388	S	P	0.528	373	388
480^3^	E	S	0.993	415	475
485	G	R	0.593	420	480
491	H	R	0.942	426	486
493	T	R	0.802	428	488

1 The position differs from alignment to protein as the alignment file contains sequence gaps.

2 Our confidence in each of these sites being positively selected is calculated using the posterior probability and summarized in the *P* values shown. *P* values vary from 0.00 (no evidence for belonging in the positively selected category) to 1.00 (100% confidence of belonging in the positively selected category).

3 Dark gray area refers to residues deemed to be false positives due to poor alignment of the *U2af1-rs* sequences.

### THREE-DIMENSIONAL STRUCTURE OF U2af1-rs PROTEINS

**Figure 4:**
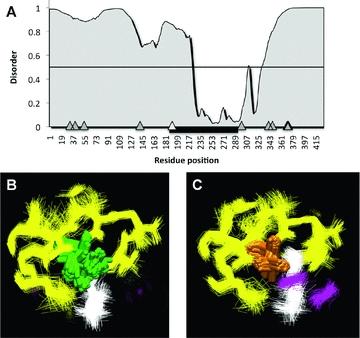
Three-dimensional structure of U2af1-rs proteins. (A) Disorder prediction. Positions of all the positively selected residues along the protein are shown as gray triangles. Position of U2af35-homologous domain shown as thick black line. Positively selected residue within this domain shown as white triangle. Thin black line denotes disorder probability 0.5. Values above this predict disorder. (B and C) Closeup view of the neighboring residues to the positively selected residue. (D) U2AF1-RS2 isoelucine. (E) U2AF1-RS1 valine. Residues within 6 Angstrom cut-off from the isoleucine residue or valine residues are colored by secondary structure: beta-sheets yellow, alpha helix purple, and coil white.

The 3-D structure of the U2af1-rs proteins was investigated to see what effects the positively selected sites might have on the overall fold stability of the protein. We carried out disorder prediction for all the U2af1-rs proteins to identify areas of the proteins that are predicted to be disordered, and areas that might have secondary and tertiary ordered structure. Predictions consistently showed high levels of disorder at the beginning and at the end of the protein, with an ordered area toward the centre (disorder probability of >0.5 is considered disordered). Figure 4A shows the predicted level of disorder across the protein for mouse *U2af1-rs1*. The positively selected amino acid changes are shown as triangles, and are observed to cluster particularly in the disordered regions. The same pattern is seen in all the U2af1-rs proteins (data not shown).

Within the nondisordered central region of the U2af1-rs proteins, a region homologous to the human U2af35 RNA binding domain was found. The crystal structure of the human U2af35 domain has been solved (Kielkopf et al. 2001). This structure was used as a template to model the structures of the homologous domains in mouse *U2af1-rs1* and *U2af1-rs2*

Only one of the positively selected amino acid changes was found to fall within the ordered region of U2af35 homology, this was the codon at position 206 in the MSA, corresponding to isoleucine in the all parent gene sequences in all species (residue position 206 in the mouse U2AF1-RS2), and a valine residue in all the retrogene sequences in all species (position 192 in mouse U2AF1-RS1). We analyzed the difference between the two sets of models and we focused on the immediate neighboring residues of the two mutations (Figure 4B and C). Many iterations of the modeling procedure are depicted; hence each residue has multiple representations of its position. Any atom within 6 Angstroms of the residue of interest is colored. In the U2AF1-RS1 structure, two residues (atoms belonging to Phenylalanine 238 and Phenylalanine 279, both magenta) are on average closer to the positively selected valine residue, compared to the isoleucine in the U2AF1-RS2 structure. The more bulky isoleucine residue of U2AF1-RS2 induces a larger perturbation in neighboring residues, pushing them away.

## Discussion

### DISPARATE MODES OF EVOLUTION FOR DIFFERENT IMPRINTED RETROGENE FAMILIES

Although studies of large gene cohorts (Cusack and Wolfe 2007) can be informative on the general trends in evolutionary rates of parent genes and retrogenes, analysis of individual retrogene-parent pairs, as in this study, can reveal much heterogeneity in evolutionary rate among retrogenes. Indeed, the four retrogenes examined here have many features in common, other than their origins as retroposition products, such as their imprinted regulation and X-chromosome derivation. However, each gene family examined showed very different evolutionary trajectories.

The *Inpp5f_v2* retrogene is evolving under a more relaxed selective constraint than its parent gene *Vma21* The Nap1l gene family has evolved under a strict regime, with a high constraint on codon evolution. However, major deletions to the *Napl15* open reading frame may have impacted on this protein's function. In the case of the Mcts gene family, selective pressure analyses results show that all gene sequences from all lineages (both parent and retrogene), are under purifying selection suggestive of evolutionary pressure to maintain the same protein function in the parent and retrogene. The *U2af1-rs1* retrogene has been under positive Darwinian selection, in contrast to its parent gene, which has been under a mixture of purifying and neutral evolutionary pressures.

### NONUNIFORM EVOLUTIONARY INNOVATION ALONG THE U2af1-rs1 PROTEIN

Regions of the U2af1-rs genes are homologous to the U2af35 RNA-binding domain. After the emergence of the ancestral *U2af1-rs1* retrogene, one residue in the homologous RNA-binding domain showed evidence of positive selection/adaptive evolution, changing from an isoleucine in the parent gene sequence to a valine in the retrogene. Our models show that this valine residue produces fewer perturbations within the core of the protein structure, compared to isoleucine. Although both residues are tolerated within the core of the modeled protein structure, the U2AF1-RS1 protein may therefore have enhanced stability compared with its parent protein.

Most of the positively selected residue changes are focused in the disordered regions of the *U2af1-rs1* retrogene. Here, constraints to maintain a particular structure may be relaxed, and so the plasticity of these disordered regions might allow the protein to “experiment” with new residues. Investigations into the possible structures of these disordered protein regions are required to ascertain the effects of these residue changes, but this is beyond the scope of this study. *U2af1-rs1* has some parallels in the imprinted plant gene *MEDEA* (Spillane et al. 2007). *MEDEA* was formed through a whole-genome duplication in plants, and subsequently underwent neofunctionalization by means of positive selection.

### *Mcts2* IN SPERMATOGENESIS

From previous studies, we know that all the parent genes in this study map to the X chromosome. It has been proposed that the significant excess of functional retrogenes produced from parent genes on the X chromosome is attributable to gene function during spermatogenesis (Emerson et al. 2004). X-linked genes are more likely to show a testis-specific expression pattern than would be expected by chance (Wang et al. 2001). However, during spermatogenesis, genes on the sex chromosomes are subject to epigenetic silencing during a process termed meiotic sex chromosome inactivation (MSCI). Many X-linked genes are downregulated in their expression, particularly at the pachytene stages, whereas autosomal genes are not (Wang et al. 2005). Autosomal copies of X-linked genes, such as *Mcts2*, might compensate for their parent's downregulation during the pachytene stages of spermatogenesis.

**Figure 5:**
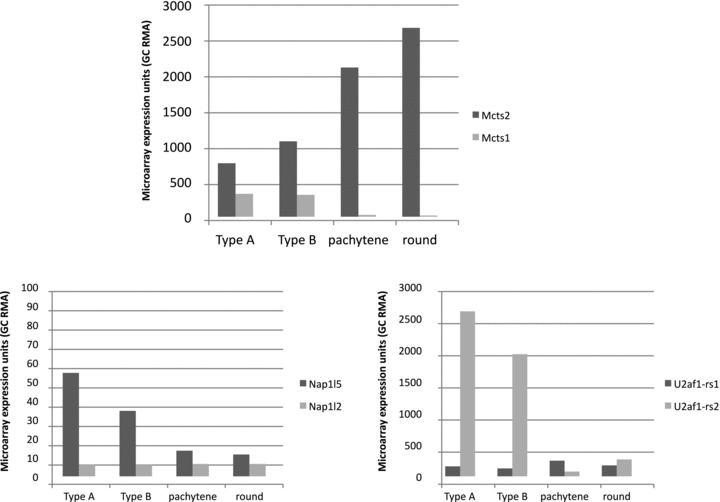
Expression for mouse retrogenes and their parent genes during spermatogenesis. GC RMA values for two biological replicates were averaged. Data were extracted from GEO dataset GDS2930 from Namekawa et al 2006. Probe identifiers were 1425018_at for Mcts1, 1451058_at for Mcts2, 1417411_at for Nap1l5, 1418046_at for Nap1l2, 1449354_at for U2af1-rs1, and 1455727_at for U2af1-rs2. There was no specific probe for Inpp5f_v2, so this gene could not be included.

Using microarray data (Namekawa et al. 2006), we tested the hypothesis that the imprinted genes in question can compensate for their parent genes. Figure 5 shows that expression of *Mcts2* in mouse increases during the pachytene stage when MSCI takes place, with *Mcts1* dropping dramatically. The strong purifying selection seen upon both genes might be acting to maintain the same protein function, with *Mcts2* substituting for *Mcts1* as it is inactivated during the later stages of spermatogenesis.

The two other gene families for which microarray probes were present (Nap1l and U2af1-rs) do not exhibit expression patterns consistent with MSCI compensation (Fig. 5). The X-linked parent genes behave as expected; *Nap1l2* remains at very low levels of expression as spermatogenesis progresses and *U2af1-rs2* shows decreasing expression. However the corresponding retrogenes do not show increased expression levels as MSCI sets in, unlike *Mcts2* This suggests that not all X-to-autosome retrogenes compensate for their parents during MSCI.

### EXPRESSION PROFILES OF RETROGENES ARE ATYPICAL

It has been suggested that retrogenes tend to show an expression bias toward the testes (Shiao et al. 2007), reviewed in Wang et al. (2001), for both evolutionary and mechanistic reasons. Mechanistically, the testes provide a “permissive” environment for transcription (Schmidt 1996; Kleene 2001), and so retrogenes that have appeared de novo, and might not possess a strong promoter, still have a chance at expression. As discussed above, evolutionary pressures also act to confer male-specific functions upon many retrogenes, particularly those that originated on the X-chromosome. However, the retrogenes discussed here show a wide expression pattern. Indeed, the Nap1l genes and *Inpp5f_v2* show very low or no expression in the testes. We compared our expression data with Potrzebowski et al. (2008), which contains expression data on the *U2af1-rs1* retrogene. Potrzebowski et al. found *U2af1-rs1* not to have a testis-specific expression pattern, but to be expressed in 14 of 14 somatic tissues examined. This finding complements the results presented here nicely.

Retrogenes are also said to emerge “out of the testes” (Vinckenbosch et al. 2006), as this tissue exhibits very high levels of transcription. Retrogenes might then go on to evolve a more broad expression pattern. Surprisingly, although the four imprinted retrogenes discussed here are of quite different ages, with *U2af1-rs1* the youngest, and *Mcts2* and *Nap1l5* older, all retrogenes display a very broad expression pattern, which in each case is similar to their parent gene. Hence, the retrogenes studied here do not seem to have emerged “out of the testis.” This is perhaps not surprising, as these retrogenes already differ from most by their imprinted status, and their position within the introns of other “host” genes. Perhaps the presence of the host gene confers a wide expression pattern on the retrogenes via access to a transcriptionally active genomic environment (L. Potrzebowski, pers. comm.).

### *Mcts2* AS A POTENTIAL ONCOGENE

In humans, the *Mcts1* (*Mct-1*) gene has been established as having oncogenic properties (Hsu et al. 2005, 2007). Like the U2af1-rs proteins, *Mcts1* is an RNA-binding protein. *Mcts1* binds RNA via a PUA domain and appears to alter cellular phenotype by interacting with mRNA and affecting translation (Reinert et al. 2006; Mazan-Mamczarz et al. 2009). Given the similarity of all the Mcts family genes, as shown by the MSA, and the high level of purifying selection present across all residues of these genes across multiple species, it seems likely that *Mcts2* and its orthologs may share similar functions and may also have oncogenic properties. There is a strong link between the phenomenon of imprinting and cancer etiology (Feinberg 2007). Considering that *Mcts2* is subject to genomic imprinting, as is *Mcts2*, potential disruption of the imprinting mechanism at the *Mcts2* locus could have major consequences for cancer development if *Mcts2* is shown to be an oncogene.

## Conclusions

The four retrogenes examined here share a number of sequence features and properties: (1) all are retrogenes, derived from a parent gene on the X chromosome, (2) all have a maternally methylated CpG island at their promoter and are surrounded by a “host” gene, (3) all are imprinted in mouse, and in human if present. However, evolution acts upon genes at the protein level, and we have shown here that from this perspective each retrogene has followed a distinct evolutionary path.

The *Mcts2* gene/protein has been maintained under strong selective pressure just like its parent gene/protein. Selective pressure upon the *Inpp5f_v2* lineage was relaxed compared with its parent lineage. The *Nap1l5* lineage has undergone a major truncation of its coding sequence. The *U2af1-rs1* lineage shows evidence for positive selection, which is synonymous with protein functional shift. Genome-wide modeling of evolutionary properties of retrogenes would doubtless have missed this individuality. Similarly, the expression patterns of these gene families do not follow the “classical” trend for a more widely expressed parent gene and retrogene expression is mostly confined to the testes.

Our case-by-case evolutionary analysis of four imprinted retrogenes has revealed their evolutionary trajectories. This information can direct further studies, particularly into the potential oncogenic properties of the *Mcts2* retrogene, and the changes in protein function predicted for *U2af1-rs1*.

## Supporting Information

The following supporting information is available for this article:

**Appendix S1.** DNA sequences.

**Table S1.** Primer sequences used.

**Table S2.** SH Test results.

**Table S3.** Description of coon evolution models used.

**Table S4.** Genomes examined for estimation of the age of retrotransposition events.

**Appendix S2.** Results of codeml analysis for all four gene families, using the gene-based phylogenies.

**Table S5.** Codon evolution results using species phylogenies for gene families where the species phylogeny fit the data equally well as the gene-based phylogeny.

**Table S6.** Alignments for each gene family.

**Table S7.** Positively selected residues for the U2af1-rs gene family using both gene-based and species-based phylogenies.
