# Supplementary material for: A Case-by-Case Evolutionary Analysis of Four Imprinted Retrogenes
Source: Evolution. 2011 May;65(5):1413–27. doi: 10.1111/j.1558-5646.2010.01213.x (PMC3107425; doi:10.1111/j.1558-5646.2010.01213.x)
Supplement: Supplementary file 1 [file evo0065-1413-SD1.doc]

**Additional File 1. Nucleotide sequences used**

**Inpp5f_v2-Vma21 family**

>*H. sapiens INPP5F_V2*

ATGCCGCGCTCTCACAGGCCTCCGCCTACCGCGCAGCGGGCTGCCGAGCCCAAGTCTGAG

GGCGTTGTGGCTATGACTTTCAAGATTTTTCTCCTTTTTGCAGGGCTTATGGTTAAAGTT

CCTGTGGGGTTATATTTTTCCTGCAAATTACTTCTTTTCCAAAGTCTGATGTTGATGTCC

CCTGAAGACAGCGGCTTTTATGCGACCATTGTTGCAGTGGTCGGGCTTCATGTGGTGCTG

GCAATTTTCGTCTTCATAGTGTGGAAGGAGGGCTTGCCCCAGTGGCGGGAAAACAAAAAC

GAATAG

>*M. musculus Inpp5f_v2*

ATGCCGCGCTCCCATCGGCAGCCGCCACCTGCGCCTCGCGCTCCAGAACCTAAGTCCGAT

GGCGTTCTCGCCATGACTTTTAAGATTTTCCTTCTTTTTGCAGGACTTATGGTTAAAGTG

CCAGTGGGGCTGTATTTTTCCTGTAAATTACTTCTTTTCCAAAGCTTGATGTTAATGTCC

CCGGAAGACAGCGCTTTTTATGCGACCATTGTCTCCGTGGTTGGGCTGCATGTAGTTTTG

GCAATTTTTGTTTTTATAGTATGGAAGGAAGGTTTGCCACAGTGGCGGGAAAACAAAAAT

GATTAG

>*R. novergicus Inpp5f_v2*

ATGCCGTGCTCCCATCGGCAGCCGCCCCCTGTGCCTCGCGCTCCAGAACCTAAGTCCGAT

GGTGTTCTCGCTATGACTTTTAAGATTTTCCTCCTTTTTGCAGGACTTATGGTTAAAGTA

CCGGTGGGGCTGTATTTTTCCTGTAAATTACTTCTTTTCCAAAGCTTGATGTTAATGTCC

CCGGAAGACAGCGCTTTTTATGCGACCATTGTCTCCGTGGTTGGGCTGCATGTCGTTTTG

GCAACTTTTGTTTTTATAGTATGGGAGGAAGGTTTGCCACAGTGGCGGGAAAACAAAAAT

GATTAG

>*C. l. familaris Inpp5f_v2*

ATGCCGCGCTCCCACCGGCCTCCCCCGACCGCCCAGCGGCCCGCCGAGCCTAAGCCCGAA

GGCAGCCTGGCCACGACTTTCAAGATTTTTCTCCTTTTCGCAGGACTCATGGTTAAAGTT

CCTGTGGGCTTGTATTTTTCCTGCAAATTGCTCCTTTTCCAGAGTCTCCTGTTAATGTCT

CCTGACGACAGTGGCTTTTACGCGACCATTGTCGCGGTGGTGGGGCTGCATGTGGTGCTG

GCGGTGTTCGTGTTCATCGTGTGGAAGGAGGGCATGCCTGACTGGCAGGAGAACAAGAGC

GAGTAG

>*B. taurus Inpp5f_v2*

ATGCCGCGCTCCCACCGGCCTCCTCCAACCGCTCAGCGGCAGGCGGAGCCCAAGCCCGAA

GGCAGCCTGGCGACCACTTTCAAGATTTTTCTCCTTTTTGCAGGACTGATGGTTAAAGTT

CCTGTGGGGTTGTATTTTTCCTGCAAATTGCTGCTTTTCCAAAGCCTCCTGTTAATGTCC

CCCGATGACAGTGGCTTTTACGCAACCATTGTCGCGGTGGTGGGGCTGCATGTGGTGCTG

GCGGTGTTTGTGTTCATCGTGTGGAAGGAGGGCATGCCTGACTGGCGGGAAGAGAAAAAC

GAGTAG

>*L. africana Inpp5f_v2*

ATGCCGCGCTCCCACAGGCATCCGCCTGCGGCCCCGCGCACCGAGGAGCCCAAGTCCGAC

GGCGTGCTGCCTATGACTTTCAAGATTTTCCTCCTTTTTGCAGGACTTATGGTCAAAGTT

CCTGTGGGGCTATATTTTTCCTGTAAATTACTCCTTTTCCAAAGTCTCATGTTAATGTCC

CCCGAAGACAGCGCCTTTTACGCGACCATCGTCGCAGTGGTGGGGCTTCATGTGGAGCTG

GCGTTTTTTTTCATCATAGTGTGGAAGGAAGGCTTGCCCCACTGGCGGGAAAACAAAAAC

GAGTAG

>*D. novemcinctus Inpp5f_v2*

ATGCCGCGCTCCCACAGGCCTCCGCCTGTCGCTCAGCGGCCCGCTGAGCCCAAGTCTGAT

GGCGTTCTGGCAATGACTTTCAAGATTTTTCTCCTTTTCGCAGGACTTATGGTCAAAGTT

CCTGTGGGGTTATATTTTTCCTGTAAATTACTCCTTTTCCAAAGTCTCATGTTAATGTCC

CCTGAAGATAGCGCCTTTTATGCGACCATCGTCGCAGTGGTTGGGCTTCATGTGGTGCTG

GCAATTTTTGTCTTCATAGTATGGAAGGAGGGCTTGCTCCAGTGGCGGGAAAACAAAAAC

GATTAG

>*H. sapiens VMA21*

ATGGAGCGCCCGGATAAGGCGGCGCTGAACGCACTGCAGCCTCCTGAGTTCAGAAATGAA

AGCTCATTAGCATCTACACTGAAGACGCTCCTGTTCTTCACAGCTTTAATGATCACTGTT

CCTATTGGGTTATATTTCACAACTAAATCTTACATATTTGAAGGCGCCCTTGGGATGTCC

AATAGGGACAGCTATTTTTACGCTGCTATTGTTGCAGTGGTCGCCGTCCATGTGGTGCTG

GCCCTCTTTGTGTATGTGGCCTGGAATGAAGGCTCACGACAGTGGCGTGAAGGCAAACAG

GATTAA

>*M. musculus Vma21*

ATGGAGCGTCTTGATAAAGCAGCGCTCAATGCGCTGCAGCCTCCAGAGTTCAGAAATGAA

AACTCTTTAGCTGCAACCCTGAAGACACTGCTGTTCTTCACAGCTTTAATGATCACGGTT

CCTATCGGCTTGTATTTTACAACGAAAGCTTATATATTTGAAGGTGCCCTTGGAATGTCT

AATAGAGACAGCTATTTTTATGCCGCAATTGTTGCTGTTGTTGCTGTTCATGTGGTTCTG

GCCCTGTTTGTCTATGTGGCCTGGAATGAAGGCTCACGACAGTGGCGTGAAGGCAAACAG

GATTAA

>*B. taurus Vma21*

ATGGAGCGCCTTGATAAAGCGGCGCTGAACGCGCTGCAGCCGTCCGACTTCAGAAATGAA

AGTTCATTAGCATCCACTCTGAAGACGCTCCTGTTCTTCACAGCTTTAATGATCACTGTA

CCTATTGGGTTATATTTCACAACTAAATCTTACGTTTTTGAAGGCGCCTTTGGGATGTCC

AATAGGGACAGCTATTTTTATGCTGCTATTGTTGCAGTGGTCGCCGTCCACGTGGTGCTG

GCCCTCTTTGTTTATGTGGCCTGGAATGAAGGCTCACGGCAGTGGCGTGAAGGCAAACAG

GATTAA

>*C. l. familiaris Vma21*

ATGGAACGCCTCGATAAAGCGGCGCTGAATGCGCTGCAGCCGCCCGACTTCAGAAATGAA

AGTTCATTAGCATCTACCCTGAAGACGCTCCTGTTCTTCACAGCTTTAATGATCACTGTA

CCTATTGGGTTATATTTCACGACTAAGTCTTATGTTTTTGAAGGCGCCTTTGGGATGTCC

AATAGGGACAGCTATTTTTATGCTGCTATTGTTGCGGTGGTCGCCGCCCACGTGGTGTTG

GCCCTCTTTGTTTATGTGGCCTGGAATGAAGGCTCACGACAGTGGCGTGAAGGCAAACAG

GATTAA

>*L. africana Vma21*

ATGGAGCGCTTGGACAAAGCGGCGCTGAATGCCCTGCAGCCGCCCGACTTCAGGAGTGAA

AGTTCATTAGCATCTACCCTGAAGACGCTCCTGTTCTTCACCGCCTTGATGATTACAGTT

CCTATCGGGTTATATTTCACGACTAAATCATACGTTTTTGAAGGCGCCTTGGGCATGTCC

AGTCGGGACAGCTATTTTTATGCCGCTATTGTGGCAGTGGTTGCTGTCCACGTGGTGCTG

GCCCTGTTCCTGTACGTGGCGTGGAACGAAGGCTCACGACAGTGGCGTGAAGGCAAACAA

GATTAA

>*D. novemcinctus Vma21*

ATGGAGCGTTTTGATAAGGCGGCCTTGAACGCGTTGCAGCCGCCCGAATTCAGAACTGAA

AGTTCATTAGCATCTACACTGAAGACGCTCCTGTTCTTCACCGCTTTAATGATCACTGTT

CCAATTGGGTTATATTTTACAACTAAATCTTACCTATTTGAAGGTGCCTTGGGGATGTCC

AATAGGGACAGCTATTTTTATGCTGCTATTGTTGCAGTGGTAGCCGTCCATGTGGTGCTG

GCCCTCTTTGTGTACGTGGCCTGGAATGAAGGCTCACGACAGTGGCGAGAAGGCAAACAG

GATTAA

>*M. domesticus Vma21*

ATGGAACGGCTCGAGAAGACGACGCAGAGCGCACTGCAGCCGCCCGAGCAGAGAAATGAAGGCTCATTAACATCGACTCTGAAGACTCTTCTGGTTTTCACAGCTTTAATGATCACCTTA

CCTATTGGATTATATTTCTCTTCTAAATCTTATGTGTTTGAAGGTTTCTTGGGGATGTCC

AATGGAGACAGCTATTTTTATGCTGCAATTGTTGCTGTTATTGCTGTTCACGTGGTACTT

GCGCTCTTTGTCTACGTCGCATGGAATGAAGGTTCACGCCAGTGGCGGGAAGGCAAACAA

GACTGA

>*T. vulpecula Vma21*

ATGGAACGGCTCGAGAAGACGACGCTGAGCGTGCTGCAGCCGCCCGAGCTGAGAAATGAAGGCTCATTAACATCGACTCTGAAGACTCTTCTGGTTTTTACAGCTTTAATGATCACCTTA

CCTATTGGGTTATATTTTTCTTCTAAATCTTATGTGTTTGAAGGTACTTTGGGGTTGTCC

AACCGAGACAGCTATTTTTATGCCGCGATTGTTGCTGTTGTTGCCGTTCACGTGGTACTT

GCGCTCTTCGTCTACGTCGCATGGAATGAAGGCTCACGCCAGTGGCGGGAAGGCAAACAA

GACTGA

**Mcts family**

>*L. africana Mcts1*

ATGGGCAAAGGAAGATTTGATGAAAAAGAAAATGTGTCCAACTGCATCCAGTTGAAAACCTCAGTTATTAAGGGTATTAAGAATCAATTGGTAGAGCAATTTCCAGGTATTGAACCATGGCTTAATCAAATCATGCCTAAGAAAGATCCTGTCAAAATAGTACGATGCCATGAACATATAGAAATCCTTACAGTAAATGGAGAGTTACTATTTTTTAGACAGAGAGAAGGTCCTTTTTATCCAACCCTAAGGTTACTTCACAAATATCCTTTTATCCTGCCACATCAGCAGGTTGATAAAGGAGCCATCAAATTTGTACTCAGTGGAGCAAATATCATGTGTCCAGGCTTAACTTCTCCTGGAGCTAAGCTTTACCCAGCTGCAGTAGACACGATTGTTGCAATAATGGCAGAAGGAAAACAGCATGCTCTGTGTGTTGGAGTCATGAAGATGTCTGCAGAAGATATTGAGAAAGTCAACAAAGGAATTGGCATTGAAAATATCCATTATTTAAATGATGGGCTGTGGCATATGAAAACATATAAATGA

>*G. gorilla Mcts1*

AAAGGAAGATTTGATGAAAAAGAAAATGTGTCCAACTGCATCCAGTTGAAAACTTCAGTTATTAAGGGTATTAAGAATCAATTGATAGAGCAATTTCCAGGTATTGAACCATGGCTTAATCAAATCATGCCTAAGAAAGATCCTGTCAAAATAGTCCGATGCCATGAACATATAGAAATCCTTACAGTAAATGGAGAATTACTCTTTTTTAGACAAAGAGAAGGGCCTTTTTATCCAACCCTAAGATTACTTCACAAATATCCTTTTATCCTGCCACACCAGCAGGTTGATAAAGGAGCCATCAAATTTGTACTCAGTGGAGCAAATATCATGTGTCCAGGCTTAACTTCTCCTGGAGCTAAGCTTTACCCTGCTGCAGTAGATACCATTGTTGCTATCATGGCAGAAGGAAAACAGCATGCTCTATGTGTTGGAGTCATGAAGATGTCTGCAGAAGACATTGAGAAAGTCAACAAAGGAATTGGCATTGAAAATATCCATTATTTAAATGATGGGCTGTGGCATATGAAGACATATAAATGA

>*E. europaeus Mcts1*

ATGGGCAAAGGAAGATTTGATGAAAAAGAAAATGTGTCCAACTGCATCCAGTTGAAAACATCAGTGATAAAGGGCATTAAAAATCAATTGATAGAGCAATTTCCAGGTATTGAACCATGGCTTAATCAGATCATGCCTAAGAAAGATCCTGTCAAAATTGTACGATGCCATGAACATATAGAAATCCTTACAGTAAATGGAGAATTGCTATTTTTTAGGCAAAGAGAAGGGCCTTTTTATCCAACCCTAAGGTTACTTCACAAATATCCTTTCATCCTGCCCCACCAGCAAGTTGATAAAGGAGCCATCAAGTTTGTACTTAGTGGAGCAAATATCATGTGTCCAGGTTTAACATCCCCTGGAGCTAAACTTTACCCTGCTGCAGTAGACACAGTTGTTGCAATCATGGCAGAAGGAAAACAACATGCCCTGTGTGTTGGAGTCATGAAGATGTCTGCGGAAGATATTGAGAAAGTCAACAAAGGAATCGGCATTGAAAATATCCATTATTTAAATGATGGGCTGTGGCATATGAAGACGTATAAATGA

>*O. anatinus Mcts1*

TCATTTGATAGATTTGATGAAAAGGAGAATGTATCCAACTGCATCCAGTTGAAAACTTCAGTTATTAAGGGCATTAAGAATCAACTGATAGACCAATTTCCTGGAATTGAACCATGGCTCAACCAAATCATGCCAAAGAAAGATCCTGTCAAAATTGTAAGATGCCATGAACATATAGAAATCCTTACAGTAAATGGAGAGTTACTATTTTTCAGGCAAAGAGAAGGAACTTTTTATCCAACGCTAAGATTACTTCATAAATATCCATTTATCCTACCACATCAACAAGTTGATAAAGGAGCCATTAAATTCGTACTTAGTGGAGCTAATATCATGTGTCCGGGCTTGACATCACCAGGAGCCAAACTTTACCCTGCTGCATCTGATACAGTTGTCGCCATAATGGCAGAGGGGAAACAACATGCATTGTGTGTGGGAGTCATGAAGATGTCTGCAGATGACATTGAGAAAGTCAATAAAGGGATCGGCATTGAAAACATTCACTATTTAAATGATGGCCTTTGGCACATGAAGACCTATAAGTGA

>*G. gallus Mcts1*

ATGTTTAAAAAATTTGATGAAAAGGAGAATGTATCGAACTGTATCCAGCTGAAGACTTCAGTTATTAAAGGTATTAAGAACCAACTGATAGACCAATTTCCTGTTATTGAACCATGGCTAAACCAAATTATGCCAAAGAAAGACCCTGTCAAAATAGTAAGATGTCATGAACATATAGAGATCCTCACTGTGAATGGAGAATTGCTGTTCTTTAGACAAAGAGAAGGGATTTTTTACCCAACTCTAAGATTACTTCACAAATATCCATTTATTCTACCACATCAGCAGGTTGATAAAGGAGCCATTAAATTTGTACTAAGTGGAGCTAATATAATGTGTCCTGGCCTGACGTCTCCTGGAGCAAAACTTTACCCTGCTGCCGTTGATACTGTTGTTGCAATAATGGCAGAGGGAAAACAACATGCATTATGTGTGGGAGTCATGAAGATGTCAGCTGATGACATTGAGAAGGTCAACAAAGGGATTGGTATTGAAAATATCCACTATTTAAATGATGGTCTTTGGCATATGAAGACATACAAGTGA

>*P. troglodytes Mcts1*

ATGGGCAAAGGAAGATTTGATGAAAAAGAAAATGTGTCCAACTGCATCCAGTTGAAAACTTCAGTTATTAAGGGTATTAAGAATCAATTGATAGAGCAATTTCCAGGTATTGAACCATGGCTTAATCAAATCATGCCTAAGAAAGATCCTGTCAAAATAGTCCGATGCCATGAACATATAGAAATCCTTACAGTAAATGGAGAATTACTCTTTTTTAGACAAAGAGAAGGGCCTTTTTATCCAACCCTAAGATTACTTCACAAATATCCTTTTATCCTGCCACACCAGCAGGTTGATAAAGGAGCCATCAAATTTGTACTCAGTGGAGCAAATATCATGTGTCCAGGCTTAACTTCTCCTGGAGCTAAGCTTTACCCTGCTGCAGTAGATACCATTGTTGCTATCATGGCAGAAGGAAAACAGCATGCTCTATGTGTTGGAGTCATGAAGATGTCTGCAGAAGACATTGAGAAAGTCAACAAAGGAATTGGCATTGAAAATATCCATTATTTAAATGATGGGCTGTGGCATATGAAGACATATAAATGA

>*B. taurus Mcts1*

ATGTTTAAGAAATTTGATGAAAAAGAAAATGTGTCCAATTGCATCCAGTTGAAAACCTCAGTTATAAAGGGTATTAAGAATCAATTGATAGAACAGTTTCCAGGTATTGAACCATGGCTTAATCAAATCATGCCTAAGAAAGATCCTGTCAAAATAGTGCGATGCCATGAACATATAGAAATCCTTACAGTAAATGGAGAGTTACTATTTTTTAGACAAAGAGAAGGGCCTTTTTATCCAACCCTAAGATTACTTCACAAATATCCTTTTATCCTGCCACATCAGCAGGTTGACAAAGGAGCCATCAAATTTGTACTCAGTGGAGCAAATATAATGTGTCCAGGTTTAACTTCTCCCGGAGCTAAACTTTACCCTGCTGCAGTAGATACGATTGTTGCAATCATGGCAGAAGGAAAACAGCATGCTCTGTGTGTTGGAGTCATGAAGATGTCTGCAGAAGATATTGAGAAAGTCAACAAAGGAATTGGCATTGAAAATATCCATTATTTAAATGATGGGCTGTGGCATATGAAGACATATAAATGA

>*C.l. familiaris Mcts1*

ATGGGCAAAGGAAGATTTGATGAAAAAGAAAATGTGTCCAACTGCATCCAGTTGAAAACCTCAGTTATAAAAGGTATTAAGAACCAATTGATAGAACAATTTCCAGGTATTGAACCATGGCTTAATCAAATCATGCCTAAGAAAGATCCTGTCAAAATAGTGCGATGCCATGAACATATAGAAATCCTTACAGTAAATGGAGAATTACTATTTTTTAGACAAAGAGAAGGGCCTTTTTATCCAACCCTAAGGTTACTTCACAAATATCCTTTTATCCTGCCACACCAGCAAGTTGATAAAGGAGCCATCAAATTTGTACTCAGTGGAGCAAATATCATGTGTCCAGGCTTAACTTCTCCTGGAGCTAAACTTTACCCTGCTGCAGTAGATACAATTGTTGCAATTATGGCAGAAGGAAAACAGCATGCTCTGTGTGTTGGAGTCATGAAGATGTCTGCAGAAGATATTGAGAAAGTCAACAAAGGAATTGGCATTGAAAATATCCATTATTTAAATGATGGGCTGTGGCATATGAAGACATATAAATGA

>*C. porcellus Mcts1*

ATGGGCAAAGGAAGATTTGATGAAAAAGAAAATGTGTCCAACTGCATCCAGTTGAAAACCTCAGTTATTAAGGGTATTAAGAATCAATTGATAGAGCAATTTCCAGGTATTGAACCATGGCTTAATCAAATCATGCCTAAGAAAGATCCTGTCAAAATAGTCCGATGCCATGAACATATAGAAATCCTTACAGTAAATGGAGAATTACTATTTTTTAGACAAAGAGAAGGGCCTTTTTATCCAACTCTAAGGTTACTTCACAAATATCCTTTTATCCTGCCACATCAGCAGGTTGATAAAGGAGCCATCAAATTTGTACTCAGTGGAGCAAATATCATGTGTCCTGGCTTAACTTCTCCAGGAGCCAAGCTTTATCCTGCTGCAGCAGATACCATTGTTGCAATCATGGCAGAAGGAAAACAGCATGCTCTATGTGTTGGAGTCATGAAGATGTCTGCAGAAGATATTGAGAAAGTCAACAAAGGAATTGGCATTGAAAATATCCATTATTTAAATGATGGGCTGTGGCACATGAAGACATATAAATGA

>*H. sapiens MCTS1*

ATGTTCAAGAAATTTGATGAAAAAGAAAATGTGTCCAACTGCATCCAGTTGAAAACTTCAGTTATTAAGGGTATTAAGAATCAATTGATAGAGCAATTTCCAGGTATTGAACCATGGCTTAATCAAATCATGCCTAAGAAAGATCCTGTCAAAATAGTCCGATGCCATGAACATATAGAAATCCTTACAGTAAATGGAGAATTACTCTTTTTTAGACAAAGAGAAGGGCCTTTTTATCCAACCCTAAGATTACTTCACAAATATCCTTTTATCCTGCCACACCAGCAGGTTGATAAAGGAGCCATCAAATTTGTACTCAGTGGAGCAAATATCATGTGTCCAGGCTTAACTTCTCCTGGAGCTAAGCTTTACCCTGCTGCAGTAGATACCATTGTTGCTATCATGGCAGAAGGAAAACAGCATGCTCTATGTGTTGGAGTCATGAAGATGTCTGCAGAAGACATTGAGAAAGTCAACAAAGGAATTGGCATTGAAAATATCCATTATTTAAATGATGGGCTGTGGCATATGAAGACATATAAATGA

>*C. jacchus Mcts1*

ATGTTCAAGAAATTTGATGAAAAAGAAAATGTGTCCAACTGCATCCAGTTGAAAACCTCAGTTATTAAGGGTATTAAGAATCAGTTGATAGAGCAATTTCCAGGTATTGAACCATGGCTTAATCAGATCATGCCTAAGAAAGATCCTGTCAAAATAGTCCGATGCCATGAACATATAGAAATCCTTACTGTAAATGGAGAATTACTATTTTTTAGACAAAGAGAAGGGCCTTTTTATCCAACCCTAAGGTTACTTCACAAATATCCTTTTATCTTGCCACACCAGCAGGTTGATAAAGGAGCCATCAAATTTGTACTCAGTGGAGCAAATATCATGTGTCCAGGCTTAACTTCTCCTGGAGCTAAGCTTTATCCTGCTGCAGTAGATACCATTGTTGCTATCATGGCAGAAGGAAAACAACATGCTCTATGTGTTGGAGTCATGAAGATGTCTGCAGAAGATATTGAGAAAGTCAACAAAGGAATTGGCATTGAAAATATCCATTATTTAAATGATGGGCTGTGGCATATGAAGACATATAAATGA

>*M. musculus Mcts1*

ATGTTCAAGAAATTTGATGAAAAAGAAAATGTGTCCAACTGCATCCAGTTGAAAACCTCGGTTATTAAGGGTATTAAAAATCAATTGCTAGAGCAATTTCCAGGTATTGAACCATGGCTTAATCAAATCATGCCTAAGAAAGACCCTGTGAAAATTGTCCGATGCCATGAACACATAGAAATCCTTACAGTAAATGGAGAATTACTGTTTTTTAGACAAAGAGAAGGGCCTTTTTATCCAACTTTAAGATTACTTCATAAATATCCTTTTATCTTGCCACATCAGCAGGTTGATAAAGGAGCCATCAAATTTGTACTCAGTGGAGCAAATATCATGTGTCCTGGCTTAACTTCTCCCGGAGCTAAGCTTTATCCTGCTGCAGTAGATACTATTGTTGCAATCATGGCAGAAGGAAAACAACATGCTTTATGTGTGGGTGTCATGAAGATGTCTGCAGAAGATATTGAGAAAGTAAACAAAGGAATTGGCATTGAAAATATCCATTATCTAAATGATGGTCTGTGGCATATGAAGACATATAAATGA

>*M. domesticus Mcts1*

ATGTTCAAGAAATTTGATGAAAAGGAGAGTGTAATAAACTGTATCCAGCTGAAAAACTCAGTTATCAAGGGCATTAAGAACCAACTGACAAGTCTCTTTCCAGAGATTAAACCATGGCTTAACCAAATTATTCCAAAGAAAGATCTCATCAAAATTGTCCGCTGCCATGAACATATTGAAATCCTCACTGTAAATGGAGAATTATTGTTTTTCAGGCAAAGGGAAGGACCTTTTCTTCCAACACTAAGATTACTCCATAAATATCCTTTTATTCTTCCACACCAGCAAGTTGATAAAGGAGCTATCAAATATGTACTAAGTGGAGCAAACATTATGTGTCCAGGCTTAACTTCTCCAGGAGCCAAGCTTTCCCCAGCTGAAGCTGACACTATTGTTGCCGTAATGGCAGAAGGAAAACAACATGCACTCTCTGTTGGTATCATGAAGATGTCTTCAGAAGAAATTAAGAAAGTCAACAAAGGGATTGGTATTGAAAATGTCCATTATTTAAATGATGGCCTTTGGCACATGAAGACATATAAGTGA

>*R. novergicus Mcts1*

ATGGGCAAAGGAAGATTTGATGAAAAAGAAAATGTGTCCAACTGCATCCAGTTGAAAACCTCGGTTATTAAGGGTATTAAAAATCAATTGCTAGAGCAATTTCCAGGTATTGAACCATGGCTTAATCAAATCATGCCTAAGAAAGATCCTGTGAAAATTGTCCGATGCCATGAACACATAGAAATCCTTACAGTAAATGGAGAGTTACTGTTTTTTAGACAAAGAGAAGGGCCTTTTTATCCAACCTTAAGATTACTTCATAAATATCCTTTTATCTTGCCACATCAGCAGGTTGATAAAGGAGCCATCAAATTTGTACTCAGTGGAGCAAATATCATGTGTCCCGGCTTAACGTCTCCTGGAGCTAAGCTTTATCCTGCTGCAGTAGATACTATTGTTGCAATCATGGCAGAAGGAAAACAACATGCTTTATGTGTGGGCGTCATGAAGATGTCTGCAGAAGATATTGAGAAAGTCAACAAAGGAATTGGCATTGAAAATATCCATTATCTAAATGATGGGCTGTGGCATATGAAGACATATAAATGA

>*M. mulatta Mcts1*

ATGGGCAAAGGAAGATTTGATGAAAAAGAAAATGTGTCCAACTGCATCCAGTTGAAAACTTCTGTTATTAAGGGTATTAAGAATCAATTGATAGAGCAATTTCCAGGTATTGAACCATGGCTTAATCAAATCATGCCTAAGAAAGATCCTGTCAAAATAGTCCGATGCCATGAACATATAGAAATTCTTACAGTAAATGGAGAATTACTCTTTTTTAGACAAAGAGAAGGGCCTTTTTATCCAACCCTAAGATTACTTCACAAATATCCTTTTATCCTGCCACACCAGCAAGTTGATAAAGGAGCCATCAAATTTGTACTCAGTGGAGCAAATATCATGTGTCCAGGCTTAACTTCTCCTGGAGCTAAGCTTTACCCTGCTGCAGTAGATACCATTGTTGCTATCATGGCAGAAGGAAAACAGCATGCTCTATGTGTTGGAGTCATGAAGATGTCTGCAGAAGACATTGAGAAAGTCAACAAAGGAATTGGCATTGAAAATATCCATTATTTAAATGATGGGCTGTGGCATATGAAGACATATAAATGA

>*P. troglodytes Mcts2*

ATGTTCAAGAAGTTTGATGAAAAGGAAAGTGTGTCCAACTGCATCCAGTTGAAAACGTCAGTTATTAAGGGCATTAAGAGCCAACTGGTAGAGCAATTTCCAGGTATTGAACCATGGCTTAATCAAATCATGCCTAAGAAAGATCCTGTCAAAATAGTCCGATGCCACGAACATACAGAAATCCTTACCGTAAGTGGGGAATTACTGTTTTTTAGACAAAGAAAGGGGCCTTTTTGTCCAACTCTAAGGTTGCTTCACAAATACCCTTTTATCCTGCCACACCAGCAGGTTGATAAAGGAGCTATCAAATTTGTACTCAGTGGCGCAAATATTATGTGTCCAGGTTTAACTTCTCCTGGAGCTAAGCTGTACCCTGCTGCGGTAGATACGATTGTAGCAGTCACAGCGGAAGGAAAACAGCATGCTCTGTGTGTTGGGGTCATGAAGATGTCTGCAGAAGATATTGAGAAAGTCAACAAAGGAATTGGCATTGAAAATATCCATTATTTAAATGATGGGCTGTGGCACATGAAGACATATAAATGA

>*C. jacchus Mcts2*

ATGTTCAAGAAGTTTGATGAAAAGGAAAGTGTGTCCAACTGCATCCAGCTGAAAACGTCAGTTATTAAGGGCATTAAGAGCCAACTGATAGAGCAGTTTCCAGGTATTGAACCATGGCTTAATCAAATCATGCCTAAGAAAGATCCTGTCAAAATAGTCCGATGCCACGAACATACAGAAATCCTTACCGTAAGTGGGGAATTACTGTTTTTTAGACAAAGAAAGGGGCCTTTTTGTCCAACTTTAAGGTTGCTTCACAAATACCCTTTTATCCTGCCACACCAGCAGGTTGATAAAGGCGCTATCAAATTTGTACTCAGCGGAGCAAATATTATGTGTCCAGGTTTAACTTCTCCTGGAGCTAAGCTGTTCCCTGCTGCAGTAGATACGATTGTTGCAGTCACAGCGGAAGGAAAACAGCATGCTCTGTGTGTTGGGGTCATGAAGATGTCTGCAGAAGACATTGAGAAAGTCAACAAAGGAATCGGCATTGAAAACATCCATTATTTAAATGATGGGCTGTGGCACATGAAGACATATAAATGA

>*M. musculus Mcts2*

ATGTTCAAGAAATTTGACGAGAAGGAAAGTGTGTCCAACTGCATCCAACTGAAAACTTCCGTTATTAAGGGTATTAAGAGCCAACTGACTGAGCAGTTTCCAGGTATCGAGCCGTGGCTTAATCAAATCATGCCTAAGAAAGATCCCGTCAAAATAGTGAGATGCCATGAACACATGGAAATCCTTACAGTCAACGGAGAATTACTGTTTTTCAGGCAGAGAAAAGGACCTTTTTATCCAACGCTAAGACTACTTCACAAATACCCGTTTATCCTGCCACACCAGCAGGTCGACAAAGGAGCCATCAAATTTGTGCTCAGTGGTGCAAATATCATGTGCCCGGGTTTAACGTCTCCTGGAGCGAAGCTCTACACTGCTGCAGTAGATACCATCGTGGCGGTCATGGCAGAGGGGAAAGAGCATGCCCTGTGTGTCGGAGTCATGAAGATGGCTGCAGCAGACATTGAGAAAATCAACAAGGGGATCGGCATTGAGAATATCCATTATCTAAATGACGGGCTGTGGCACATGAAGACATATAAGTGA

>*P. p. abelii Mcts2*

ATGTTCAAGAAGTTTGATGAAAAGGAAAGTGTGTCCAACTGCATCCAGTTGAAAACGTCAGTTATTAAGGGCATTAAGAGCCAACTGGTAGAGCAATTTCCAGGTATTGAACCATGGCTTAATCAAATCATGCCTAAGAAAGATCCTGTCAAAATAGTCCGATGCCACGAACATACAGAAATCCTTACCGTAAGTGGGGAATTACTGTTTTTTAGACAAAGAAAGGGGCCTTTTTGTCCAACTCTAAGGTTGCTTCACAAATACCCTTTTATCCTGCCACACCAGCAGGTTGATAAAGGAGCTATCAAATTTGTACTCAGCGGCGCAAATATTATGTGTCCAGGTTTAACTTCTCCTGGAGCTAAGCTGTACCCTGCTGCGGTAGATACGATTGTAGCAGTCACGGCGGAAGGAAAACAGCATGCTCTGTGTGTTGGGGTCATGAAGATGTCTGCAGAAGATATTGAGAAAGTCAACAAAGGAATCGGCATTGAAAACATCCATTATTTAAATGATGGGCTGTGGCACATGAAGACATATAAATGA

>*R. novergicus Mcts2*

ATGTTCAAGAAATTTGACGAGAAAGAAAGTGTGTCCAACTGCATCCAACTGAAAACTTCCGTTATTAAGGGTATTAAGAGCCAACTGACTGAGCAGTTTCCAGGTATCGAGCCGTGGCTTAATCAAATCATGCCTAAGAAAGATCCCGTCAAAATAGTGAGATGCCATGAACACATGGAAATCCTTACAGTCAACGGAGAATTACTGTTTTTCAGGCAGAGAAAAGGACCTTTTTATCCAACGCTAAGATTACTTCACAAATACCCGTTTATCCTGCCACACCAGCAGGTCGACAAAGGAGCCATCAAATTTGTGCTCAGTGGTGCAAATATCATGTGTCCGGGTTTAACATCTCCTGGAGCGAAGCTCTACACTGCTGCAGTAGATACCATCGTGGCGGTCATGGCAGAAGGGAAAGAGCATGCCCTGTGTGTCGGAGTCATGAAGATGGCAGCAGCAGACATTGAGAAGATCAACAAGGGGATCGGCATTGAGAATATCCATTATCTAAATGACGGGCTGTGGCACATGAAGACATATAAGTGA

>*H. sapiens MCTS2*

atgttcaagaagtttgatgaaaaggaaagtgtgtccaactgcatccagttgaaaacgtcagttattaagggcattaagagccaactggtagagcaatttccaggtattgaaccatggcttaatcaaatcatgcctaagaaagatcctgtcaaaatagtccgatgccacgaacatacagaaatccttaccgtaagtggggaattattgttttttagacaaagaaaggggcctttttgtccaactctaaggttgcttcacaaatacccttttatcctgccacaccagcaggttgataaaggagctatcaaatttgtactcagtggcgcaaatattatgtgtccaggtttaacttctcctggagctaagctgtaccctgctgcagtagatacgattgtagcagtcacagcggaaggaaaacagcatgctctgtgtgttggggtcatgaagatgtctgcagaagatattgagaaagtcaacaaaggaattggcattgaaaatatccattatttaaatgatgggctgtggcacatgaagacatataaatga

**Nap1l family**

>*P. troglodytes Nap1l*5

ATGGCCGACTCGGAAAACCAGGGGCCTGCGGAGCCTAGCCAGGCGGCGGCAGCGGCGGAGGCAGCGGCGGAGGAGGTAATGGCGGAAGGCGGTGCGCAGGGTGGAGACTGTGACAGCGCGGCTGGTGACCCTGACAGCGCGGCTGGTGAGATGGCTGAGGAGCCCCAGACCCCTGCAGAGAATGCCCCAAAGCCGAAAAATGACTTTATCGAGAACCTGCCTAATTTGGTGAAACGCCCAATAATGGCCCTCAAAAAGCTGCAGAAGCGATGCGATAAGATAGAAGCCAAATTTGATAAGGAATTTCAGGCTCTGGAAAAAAAGTATAATGACATCTATAAGCCCCTACTCGCCAAGATCCAAGAGCTCACCGGCGAGATGGAGGGGTGTGCATGGACCTTGGAGGGGGAGGAGGAGGAGGAAGAGGAGTACGAGGATGACGAGGAGGAGGGGGAAGACGAGGAGGAGGAGGAGGCTGCGGCAGAGGCTGCCGCGGGGGCCAAACATGACGATGCCCACGCCGAGATGCCTGATGACGCCAAGAAGTAA

>*B. taurus Nap1l5*

ATGGCTGACTCTCAGAACCAGGGCTCTGCGGAGCCGAGCCAGGCAGCGGCGGCGGCAGCAGCAGCAGATGCGGCCGCGGCAGCGGAGGAGGTAATGGCGGAAGGTGGTGCACAGGGGGGAGATTCTGACAGCGCGTCCAGCGACTCCGACGGTGTGGTCGGTCAGATGGCTGAGGAGCCCCAGACCCCTGCAGAGAATGCACCAAAGCCTAGAAATGACTTTATCGAGAGCCTGCCTAATTCGGTAAAATGCCGAGTCCTGGCCCTCAAAAAGCTGCAGAAGCGATGCGATAAGATAGAAGCCAAATTTGACAAGGAATTCCAGGCTCTGGAGAAAAAGTATAACGATATCTATAAGCCCTTACTTGCTAAGATCCAAGAGCTCACCGGTGAGATGGAGGGGTGTGCATGGACCTTAGAGGGTGATGAGGAGGATGATGATGATGACGAGTACGAGGATGAGGAGGAGGGAGAGGAGGAGGACGAGGAGGAAGAAGAGCCTGCAGCAGAGGCTGCGGGGACCGCTGCCGCCAAAGATGAGGGTCCCCACTCTGCAGTGCCTGATGACGCCAAGAAATAA

>*C. l. familaris Nap1l5*

ATGGCTGACTCGGAAAACCAGGGGCCTGCGGAGCCGAGCCAGGCGGCGGCGGAGGCGGCGGAGGAGGTAATGGCGGAAGGCGGCGCGCAGGGGGGAGACTCGGACAGCGCGGCCGGTCCGACGGCCGAGGAGCCCCAGACCCCCGCGGACAACGCGCCGAAGCCCAAAAATGACTTTATCGAGAGCCTGCCCAACTCGGTGAAGTGCCGAGTCCTGGCCCTCAAAAAGCTGCAGAAGCGCTGCGATAAGATAGAAGCCAAATTTGATAAGGAATTTCAGGCTCTGGAAAAAAAGTATAACGACATCTATAAGCCCCTACTTGCCAAGATCCAAGAGCTCACCGGTGAGATGGAGGGGTGTGCATGGACCTTAGAGGGGGAGGAGGACGACGACGACGAGGAAGAGTACGAGGATGAGGAGGAGGGGGAGGAGGAGGAGGAGGAGGAGGAGGAAGCTGCGGCGGAGGCTGCTGTGGAGGCGGCGGCTGCCAAAGATGAGGGTCCCCACTCTGCAGTGCCTGATGACGCCAAGAAA

>*H. sapiens NAP1L5*

ATGGCCGACTCGGAAAACCAGGGGCCTGCGGAGCCTAGCCAGGCGGCGGCAGCGGCGGAGGCAGCGGCAGAGGAGGTAATGGCGGAAGGCGGTGCGCAGGGTGGAGACTGTGACAGCGCGGCTGGTGACCCTGACAGCGCGGCTGGTCAGATGGCTGAGGAGCCCCAGACCCCTGCAGAGAATGCCCCAAAGCCGAAAAATGACTTTATCGAGAGCCTGCCTAATTCGGTGAAATGCCGAGTCCTGGCCCTCAAAAAGCTGCAGAAGCGATGCGATAAGATAGAAGCCAAATTTGATAAGGAATTTCAGGCTCTGGAAAAAAAGTATAATGACATCTATAAGCCCCTACTCGCCAAGATCCAAGAGCTCACCGGCGAGATGGAGGGGTGTGCATGGACCTTGGAGGGGGAGGAGGAGGAGGAAGAGGAGTACGAGGATGACGAGGAGGAGGGGGAAGACGAGGAGGAGGAGGAGGCTGCGGCAGAGGCTGCCGCGGGGGCCAAACATGACGATGCCCACGCCGAGATGCCTGATGACGCCAAGAAGTAA

>*M. mulatta Nap1l5*

ATGGCCGACTCGGAAAACCAGGGGCCTGCGGAGCCTAGCCAGGCGGCGGCAGCGGCGGAGGCAGCGGCTGGTCAGATGGCTGAGGAGCCCCAGACCCCTGCAGAGAATGCCCCAAAGCCGAAAAATGACTTTATCGAGAGCCTGCCTAATTCGGTGAAATGCCGAGTCCTGGCCCTCAAAAAGCTGCAGAAGCGATGCGATAAGATAGAAGCCAAATTTGATAAGGAATTTCAGGCTCTGGAAAGAAAGTATAATGACATATATAAGCCCCTACTCGCCAAGATCCAAGAGCTCACCGGCGAGATGGAGGGGTGTGCATGGACCTTGGAGGGGGAGGAGGAGGGAGGAAGAGGAGTACGAGGATGA

>*C. jacchus Nap1l5*

ATGGCCGACTCGGAAAACCAGGGGCCCGCGGAGCCCAGCCAGGCAGCGGCGGCAGCTGCGGAGGCAGCGGCGGAGGAGGTAATGGCGGAAGGCGGTGCACAGGGTGGAGACTGTGACAGCGCGGCTGGTGACTCTGACGGCGCGGCTGGTCAGATGGCTGAGGAGCCCCAGACCGCTACGGAGAATGCCACAAAGCCGAAAAATGACTTTATCGAGAGCCTGCCTAATTCGGTGAAATGCCGAGTCCTGGCCCTCAAAAAGCTGCAGAAGCGATGCGATAAGATAGAAGCCAAATTTGATAAGGAATTTCAGGCTCTGGAAAGAAAGTATAATGAAATCTATAAGCCACTGCTCGCCAAGATCCAAGAGCTCACCGGCGAGATGGAGGGGTGTGCATGGACCTTGGAGGGGGAGGAGGAGGAGGAGGAAGAGTACGAGGATGAGGAGGAGGGGGAAGAGGAGGAGGAGGAGGAGGCTGCGGCAGAAGCTGCCGCGGGGTCCAAACATGACGATGCCAACGCCGAGATGCCTGATGACGCCAAGAAGTAA

>*M. musculus Nap1l5*

ATGGCCGACCCCGAGAAGCAGGGACCCGCTGAGAGCCGCGCCGAGGACGAGGTAATGGAAGGCGCCCAGGGTGGCGAAGATGCCGCGACCGGTGACAGTGCCGCTGCTCCCGCGGCCGAGGAGCCCCAGGCCCCCGCGGAGAACGCGCCCAAGCCCAAAAAAGACTTTATGGAGAGCTTGCCCAATTCCGTGAAATGCCGGGTTCTGGCGCTCAAAAAGCTGCAGAAGCGCTGCGATAAGATCGAGGCCAAGTTTGACAAGGAATTCCAGGCTCTGGAGAAGAAGTACAACGATATCTACAAGCCCCTGCTCGCCAAGATCCAGGAGCTCACCGGAGAGATGGAGGGCTGCGCGTGGACCCTGGAGGGAGAGGATGATGAAGATGACGAGGAAGAGGACGACGAGGAGGAGGAGGAGGAAGAAGAGGCTGCAGCTGGCGCAACTGGGGGTCCCAACTTCGCCAAGAAGTGA

>*P. p. abelii Nap1l5*

atggccgactcggaaaaccaggggcctgcggagcctagccaggcggcggcggcagcggaggcagcggcggaggaggtaatggcggaaggcggtgcgcagggtggagactgtgacagcgcggctggtgaccctgacagcgcggctggtgagatggctgaggaaccccagacccctgcagagaatgccccaaagccgaaaaatgactttatcgagagcctgcctaattcggtgaaatgccgagtcctggccctcaaaaagctgcagaagcgatgcgataagatagaagccaaatttgataaggaatttcaggctctggaaaaaaagtataatgacatctataagcccctactcgccaagatccaagagctcaccggcgagatggaggggtgtgcatggaccttggagggggaggaggaggaggaagaggagtacgaggatgacgaggaggagggggaagaagaggaggaggaggaggaggctgcggcagaggctgccgcgggggccaaacatgacgatgcccacgccgagatgcctgatgacgccaagaagtaa

>*R. novergicus Nap1l5*

ATGGCCGACCCCGAGAAGCAGGGACCCGCTGAGAGCCGCGCCGAGGACGAGGTAATGGAGGGCGCTCAGGGTGGCGAGGATGCAGCAACCGGTGACAGTGCCACTGCACCCGCGGCCGAGGAGCCCCAGGCCCCCGCGGAGAATGCGCCCAAGCCCAAAAATGACTTTATCGAGAGCTTGCCCAATCCCGTCAAGTGCCGGGTTCTGGCGCTCAAAAAGCTGCAGAAGCGCTGCGATAAGATCGAGGCGAAATTTGACAAGGAATTCCAGGCTCTGGAGAAGAAGTACAATGATATCTACAAGCCCCTACTCGCCAAGATCCAGGAACTCACCGGAGAGATGGAGGGCTGCGCGTGGACCCTGGAGGGAGAGGATGATGAAGACGACGAGGAAGAAGAAGATGAGGAGGAGGAAGAAGAGGAGGCTGCAGCTGGCGCAACTGGGGGTCCCGACTCTGCCGAGAAGTGA

>*H. sapiens NAP1L3*

ATGGCAGAAGCAGATTTTAAAATGGTCTCGGAACCTGTCGCCCATGGGGTTGCCGAAGAGGAGATGGCTAGCTCGACTAGTGATTCTGGGGAAGAATCTGACAGCAGTAGCTCTAGCAGCAGCACTAGTGACAGCAGCAGCAGCAGCAGCACTAGTGGCAGCAGCAGCGGCAGCGGCAGCAGCAGCAGCAGCAGCGGCAGCACTAGCAGCCGCAGCCGCTTGTATAGAAAGAAGAGGGTACCTGAGCCTTCCAGAAGGGCGCGGCGGGCCCCGTTGGGAACAAATTTCGTGGATAGGCTGCCTCAGGCAGTTAGAAATCGTGTGCAAGCGCTTAGAAACATTCAAGATGAATGTGACAAGGTAGATACCCTGTTCTTAAAAGCAATTCATGATCTTGAAAGAAAATATGCTGAACTCAACAAGCCTCTGTATGATAGGCGGTTTCAAATCATCAATGCAGAATACGAGCCTACAGAAGAAGAATGTGAATGGAATTCAGAGGATGAGGAGTTCAGCAGTGATGAGGAGGTGCAGGATAACACCCCTAGTGAAATGCCTCCCTTAGAGGGTGAGGAAGAAGAAAACCCTAAAGAAAACCCAGAGGTGAAAGCTGAAGAGAAGGAAGTTCCTAAAGAAATTCCTGAGGTGAAGGATGAAGAAAAGGAAGTTCCTAAAGAAATTCCTGAGGTAAAGGCTGAAGAAAAAGCAGATTCTAAAGACTGTATGGAGGCAACCCCTGAAGTAAAAGAAGATCCTAAAGAAGTCCCCCAGGTAAAGGCAGATGATAAAGAACAGCCTAAAGCAACAGAGGCTAAGGCAAGGGCTGCAGTAAGAGAGACTCATAAAAGAGTTCCTGAGGAAAGGCTTCAGGACAGTGTAGATCTTAAAAGAGCTAGGAAGGGAAAGCCTAAAAGAGAAGACCCTAAAGGCATTCCTGACTATTGGCTGATTGTTTTAAAGAATGTTGACAAGCTCGGGCCTATGATTCAGAAGTATGATGAGCCCATTCTGAAGTTCTTGTCGGATGTTAGCCTGAAGTTCTCAAAACCTGGCCAGCCTGTAAGTTACACCTTTGAATTTCATTTTCTACCCAACCCATACTTCAGAAATGAGGTGCTGGTGAAGACATATATAATAAAGGCAAAACCAGATCACAATGATCCCTTCTTTTCTTGGGGATGGGAAATTGAAGATTGCAAAGGCTGCAAGATAGACTGGAGAAGAGGAAAAGATGTTACTGTGACAACTACCCAGAGTCGCACAACTGCTACTGGAGAAATTGAAATCCAGCCAAGAGTGGTTCCTAATGCATCATTCTTCAACTTCTTTAGTCCTCCTGAGATTCCTATGATTGGGAAGCTGGAACCACGAGAAGATGCTATCCTGGATGAGGACTTTGAAATTGGGCAGATTTTACATGATAATGTCATCCTGAAATCAATCTATTACTATACTGGAGAAGTCAATGGTACCTACTATCAATTTGGCAAACATTATGGAAACAAGAAATACAGAAAATAA

>*C. jacchus Nap1l3*

ATGGCAGAAGCAGATTTTAAAGAGGTCTCGGAACCTGTCGCCCAAAGTGTTGCCGAAGAGGAGATGGCTAGCTCGGCTAGTGATTCTGGGGAAGAATCTGACAGCAGTAGCTCTAGCAGCAGCACTAGTAGCAGTAGCAGCAGCAGCAGTAGCAGCAGCAGCAGCAGCAGCAGCGGCAGTAGCAGCACTAGCAGCGGCAGCGGCTTATATAGAAAGAAGAGGGTACCTGAGCCTTCCAGAAGGGCGCGGCGGGCTCCGTGGAGAACAGATTTCGTGATTAAGCTGCCTCAGGCTGTTAGAAATCGTGTGCAAGCGCTTAGAAACATTCAAGATGAATGTGACAAGGTAGACACGATGTTCTTAAAAGCAATTCATGATCTTGAAAGAAAATATGCTGAACTCAACAAGCCTCTGTATGATAGGCGGTTTCAAATCATCAATGCAGAATACGAGCCTACAGAAGAAGAGTGTGAATGGAATTCAGAGGATGAGGAGTTCAGCAGTGATGAGGAGGTGCAGGATGACACCCTTAGTGAAATGCCTCCCTTAGAGGGTGAGGAAGAAGAAAAGCCTAAAGAAAACCCGGAGGTGAAGGCTGAAGAGAAGGAAGTTCCTAAAGAAATTCCTGAGGTAAAGGATGAAGAAAAGGAAGTTCCTAAAGAAATTCCTGAGGTAAAGGATGAGGAAAAGGAAGTTCCTAAAGAAATTCCTGAGGTAAAGGATGAGGAAAAAGAAGTTCCTAAAGAAATTGCTGAGGTAATGGATGAAGAAAAGGTAATTCCTAAAGAAATTGCTGAGGTAATGGATGAAGAAAAGGTAATTCCTAAAGAAATTGCTGAGGTAATGGATGAAGAAAAGGTAGTTCCTAAAGAAATTGCTGAGGTAAAGGCTGAAGAAAAAGCAGATTCTGAAGACGGTATGGAGGCAATCCCTGAAGTCAAAGAAGATCCTAAAGAAGCCCCCCAGGCAAAGGCCGAAGATAAAGAACAGCCTAAAGCAACAGAGGCTAAGCCAAGGGCTGGAGTAAGAGAGGCTCATAAAAGAGTTCCTGAGGAAAGGCTTCCGGAAAGAGTAAGTCTTAAAAGAGCTAGGAGGGGAAAGCCTAAAAGAGAAGACCCTAAAGGCATTCCTGACTATTGGCTGATTGTTTTAAAGAATGTTGACAAGCTGGGGCCTATGATTCAGAAGTATGATGAGCCGATTCTGAAGTTCTTGTCGGATGTTAACCTGAAGTTCTCAAAACCTGGCCAGCCTGTAAGTTACACCTTTGAATTTCATTTTCTACCCAATCCATATTTCAGAAATGAGGTGCTGGTAAAGACATATATAATAAAGTCAAAACCAGATCACCATGATCCCTTCTTTTCTTGGGGATGGGAAATTGAAGATTGCAAAGGCTGCAAGATAGACTGGAGAAGAGGAAAAGATGTTACGGTGAAAACTATCCAGAGTCGCACAACTGCTACTGGAGAAATCCAGCCAAGAGTGGTTCCTAATTCATCATTCTTCAATTTCTTTAGCCCTCCTGAGATTCCTATGATTGGAAAGCTGGAACCACAAGAAGATGCTATCCTTGATGAGGACTTTGAAATTGGGCAGATTTTACATGATAATGTCATCCTGAAGTCAATCTATTACTATACTGGAGAAGTCAATGAATACGGTTCCTACTATGGAGTTGGCAAAGATTACGGAAACAGGAAGTATAGAAAATAA

>*M. musculus Nap1l3*

ATGGCAGAAGCGGATCCTAAAATGGTCACAGAACCTGGTGCCCATGGGGTTGCTGAAGAGGCGATGGCTAGCACAGCTTGTGATTCTGGGGATGAATCTGACAGCAATAGCTCTAGCAGTACCAATAGTTGCAGCAGCAGCGGCAGCAGCAGCAGCGGCAGCAGCAGTAGCAGCAGCAGCAGTAGCAGCAGCAGCAGCAGCAGCAGCAGTAGCAGTAGCGGTAGCAGTGGCAGCAGCAGCAATGGCAGTCATTTGAACCGAAAGAAGAGGGTACCTGAGCCTTCCAGAAGGGCCCAGCGACGTCCCTCCGGGAAACTTTTCTTGGATAAGCTGCCCCAAGCCGTAAGAAATCGGGTGCAGGCACTCAGAAATATTCAGAATGAGTGTGACAAGGTAGACACCTTGTTCTTAAGGGCAATTCATGATCTTGAAAGAAAGTATGCTGAACTCAATAAGCCTCTATATGATAAGCGTTTTCAGATCATAAATGCAGAATATGAGCCTACAGAGGAAGAATGTGAATGGAATTCAGAAGAAGAGTTCAGTGGTGATGAAGAAATGCAGGATGACACACCTAATGAAATGCCACCCTTAGAGGGTGAGGAGGAAGAAGAAAGCTGTAATGAAAAAGCTGAAGTGAAGGAAGAAGGAACACATGTTCCAGAAGAAGTTCCTGAGGCAAAAGTTGAAGAAGAGGAGGCTCCCAAAGAAACTCCTGAGGTGAAAACTGAAGAAAAAGACATTCCAAAAGAAGGTGCTGAAGAAAAAGCTGAAGAACAGGAATCCTCTAAAGAAATTCCTGAGGTAAAAGGTGAAGAAAAAGCAGACTCTACGGATTGTATAGATATAGCTCCTGAAGAAAAAGAAGACGTCAAAGAAGTTACCCAGGCAAATACAGAAAATAAGGATCAACCTACAGAAGAATTTACACCAAGGGCTCCAGCAAGAGAGGCTCAAAAAAGGGTCCCTGAGACAAGGCCTGAAGAAGGAGTCAATATTAAAAGGGCTCGAAAGGGAAAACCTAAGAAAGAAGATCCTAAAGGTATTCCTGACTACTGGCTGACTGTTTTAAAGAATGTTGATAAGCTTGGGCCTATGATTCAAAAGTGTGATGAACCCATTTTGAAGTTCTTATCTGATGTGAGCCTGAAGTTCTCAAACCCTGGCCAGCCTATTGGTTACACTTTTGAATTTCATTTCCTACCTAACCCATACTTCAGAAATGAGCTCCTGATGAAGACATACATAATAAGGTCAAAACCAGATCACTACGACCCGTTCTTCGCGTGGGGATGGGAAATTGAAGAGTGTAAAGGCTGCAAAATAGACTGGAGACGAGGAAAAGATGTTACGGTGACAACCACCCGGAGTCGCCCTGGTATTACAGGGGAAATTGAAGTCCAGCCAAGAGTGGTTCCTAATGCATCCTTCTTCAATTTCTTCAGTCCTCCTGAGATTCCTTTGATTGGGAAGCTGGAACCAAGAGAAGATGCTATCCTTGATGAGGACTTTGAGATTGGTCAAATTTTGCATGATAATGTCATCTTGAAGTCAATCTATTACTTCACAGGAGAAATCAATGATCCCTACTACCATGACTTCAGGGATTATGGAAATAGGAAGTACTACAAGTAG

>*P. p. abelii Nap1l3*

ATGGCAGAAGCAGATTTTAAAATGGTCTCGGAACCTGTCGCCCATGGGGTTGCCGAAGAGGAGATGGCTAGCTCGACTAGTGATTCTGGGGAAGAATCTGACAGCAGTAGCTCTAGCAGCAGCACTAGTGGCAGCAGCAGCAGCAGCAGCACTAGTGGCAGCAGCAGCAGCAGCGGCAGCGGCAGCAGCAGCAGCAGCAGCGGCAGCGGCAGCACTAGCAGTCGCAGCCGCTTGTATAGAAAGAAGAGGGTACCTGAGCCTTCCAGAAGGGCGCGGCGGGCCCCGTTGGGAACAAATTTCGTGGATAGGCTGCCTCAGGCAGTTAGAAATCGTGTGCAAGCGCTTAGAAACATTCAAGATGAATGTGACAAGGTAGATACCCTGTTCTTAAAAGCAATTCATGATCTTGAAAGAAAATATGCTGAACTCAACAAGCCTCTGTATGATAGGCGGTTTCAAATCATCAATGCAGAATACGAGCCTACAGAAGAAGAATGTGAATGGAATTCAGAGGATGAGGAGTTCAGCAGTGATGAGGAGGTGCAGGATAACACCCCTAGTGAAATGCCTCCCTTAGAGGGTGAGGAAGAAGAAAACCCTAAAGAAAACCCAGAGGTGAAAGCTGAAGAGAAGGAAGTTCCTAAAGAAATTCCTGAGGTGAAGGATGAAGAAAAGGAAGTTCCTAAAGAAATTCCTCAGGTAAAGGCTGAAGAAAAAGCAGATTCTAAAGACTGTATGGAGGCAACCCCTGAAGTAAACGAAGATCCTAAAGAAGCGCCCCAGGTAAAGGCAGATGATAAAGAACAGCCTAAAGCAACAGAGGCTAAGGCAAGGGCTGCGGTAAGAGAGGCTCATAAAAGAGTTCCTGAGGAAAGGCTTCAGGACAGTGTAGATCTTAAAAGAGCTAGGAAGGGAAAGCCTAAAAGAGAAGACCCTAAGGGCATTCCTGACTATTGGCTGATTGTTCTAAAGAATGTTGACAAGCTCGGGCCTATGATTCAGAAGTATGATGAGCCCATTCTGAAGTTCTTGTCGGATGTTAGCCTGAAGTTCTCAAAACCTGGCCAGCCTGTAAGTTACACCTTTGAATTTCATTTTCTACCCAATCCATACTTCAGAAATGAGGTGCTGGTGAAGACATATATAATAAAGTCAAAACCAGATCACAATGATCCCTTTTTTTCTTGGGGATGGGAAATTGAAGATTGCGAAGGCTGCAAGATAGACTGGAGAAGAGGAAAAGATGTTACTGTGACAACTACCCAGAGTCGCACAACTGCTACTGGAGAAATTGAAATCCAGCCAAGAGTGGTTCCTAATGCATCATTCTTCAACTTCTTTAGTCCTCCTGAGATTCCTATGATTGGGAAGCTGGAACCACGAGAAGATGCTATCCTGGATGAGGACTTTGAAATTGGGCAGATTTTACATGATAATGTCATCCTGAAATCAGTCTATTACTATACTGGAGAAGTCAATGGTACCTACTATCAATTTGGCAAACATTATGGAAACAAGAAATACAGAAAATAA

>*R. novergicus Nap1l3*

ATGGCAGAAGCGGATCCTAAAATGGTCACAGAACCTGGTGCCCATGGGGTTGCTGAAGAGGCGATGGCTAGCACAGCTTGTGATTCTGGGGATGAATCTGACAGCAATAGCTCTAGCAGTACCAATAGTTGCAGCAGCAGCGGCAGCAGCAGCAGCGGCAGCAGCAGTAGCAGCAGCAGCAGTAGCAGCAGCAGCAGCAGCAGCAGCAGTAGCAGTAGCGGTAGCAGTGGCAGCAGCAGCAATGGCAGTCATTTGAACCGAAAGAAGAGGGTACCTGAGCCTTCCAGAAGGGCCCAGCGACGTCCCTCCGGGAAACTTTTCTTGGATAAGCTGCCCCAAGCCGTAAGAAATCGGGTGCAGGCACTCAGAAATATTCAGAATGAGTGTGACAAGGTAGACACCTTGTTCTTAAGGGCAATTCATGATCTTGAAAGAAAGTATGCTGAACTCAATAAGCCTCTATATGATAAGCGTTTTCAGATCATAAATGCAGAATATGAGCCTACAGAGGAAGAATGTGAATGGAATTCAGAAGAAGAGTTCAGTGGTGATGAAGAAATGCAGGATGACACACCTAATGAAATGCCACCCTTAGAGGGTGAGGAGGAAGAAGAAAGCTGTAATGAAAAAGCTGAAGTGAAGGAAGAAGGAACACATGTTCCAGAAGAAGTTCCTGAGGCAAAAGTTGAAGAAGAGGAGGCTCCCAAAGAAACTCCTGAGGTGAAAACTGAAGAAAAAGACATTCCAAAAGAAGGTGCTGAAGAAAAAGCTGAAGAACAGGAATCCTCTAAAGAAATTCCTGAGGTAAAAGGTGAAGAAAAAGCAGACTCTACGGATTGTATAGATATAGCTCCTGAAGAAAAAGAAGACGTCAAAGAAGTTACCCAGGCAAATACAGAAAATAAGGATCAACCTACAGAAGAATTTACACCAAGGGCTCCAGCAAGAGAGGCTCAAAAAAGGGTCCCTGAGACAAGGCCTGAAGAAGGAGTCAATATTAAAAGGGCTCGAAAGGGAAAACCTAAGAAAGAAGATCCTAAAGGTATTCCTGACTACTGGCTGACTGTTTTAAAGAATGTTGATAAGCTTGGGCCTATGATTCAAAAGTGTGATGAACCCATTTTGAAGTTCTTATCTGATGTGAGCCTGAAGTTCTCAAACCCTGGCCAGCCTATTGGTTACACTTTTGAATTTCATTTCCTACCTAACCCATACTTCAGAAATGAGCTCCTGATGAAGACATACATAATAAGGTCAAAACCAGATCACTACGACCCGTTCTTCGCGTGGGGATGGGAAATTGAAGAGTGTAAAGGCTGCAAAATAGACTGGAGACGAGGAAAAGATGTTACGGTGACAACCACCCGGAGTCGCCCTGGTATTACAGGGGAAATTGAAGTCCAGCCAAGAGTGGTTCCTAATGCATCCTTCTTCAATTTCTTCAGTCCTCCTGAGATTCCTTTGATTGGGAAGCTGGAACCAAGAGAAGATGCTATCCTTGATGAGGACTTTGAGATTGGTCAAATTTTGCATGATAATGTCATCTTGAAGTCAATCTATTACTTCACAGGAGAAATCAATGATCCCTACTACCATGACTTCAGGGATTATGGAAATAGGAAGTACTACAAGTAG

>*P. troglodytes Napl13*

ATGGCAGAAGCAGATTTTAAAATGGTCTCGGAACCTGTCGCCCGTGGGGTTGCCGAAGAGGAGATGGCTAGCTCGACTAGTGATTCTGGGGAAGAATCTGACAGCAGTAGCTCTAGCAGCAGCACTAGTGACAGCAGCAGCAGCAGCAGCACTAGTGGCAGCAGCAGCGGCAGCGGCAGCAGCAGCAGCAGCAGCGGCAGCACTAGCAGCCGCAGCCGCTTGTATAGAAAGAAGAGGGTACCTGAGCCTTCCGGAAGGGCGCGGCGGGCTCCGTTGGGAACAAATTTCGTGGATAGGCTGCCTCAGGCAGTTAGAAATCGTGTGCAAGCGCTTAGAAACATTCAAGATGAATGTGACAAGGTAGATACCCTGTTCTTAAAAGCAATTCATGATCTTGAAAGAAAATATGCTGAACTCAACAAGCCTCTGTATGATAGGCGGTTTCAAATCATCAATGCAGAATACGAGCCTACAGAAGAAGAATGTGAATGGAATTCAGAGGATGAGGAGTTCAGCAGTGATGAGGAGGTGCAGGATAACACCCCTAGTGAAATGCCTCCCTTAGAGGGTGAGGAAGAAGAAAACCCTAAAGAAAACCCAGAGGTGAAAGCTGAAGAGAAGGAAGTTCCTAAAGAAATTCCTGAGGTGAAGGATGAAGAAAAGGAAGTTCCTAAAGAAATTCCTGAGGTAAAGGCTGAAGAAAAAGCAGATTCTAAAGACTGTATGGAGGCAACCCCTGAAGTAAAAGAAGATCCTAAAGAAGTCCCCCAGGTAAAGGCAGATGATAAAGAACAGCCTAAAGCAACAGAGGCTAAGGCAAGGGCTGCAGTAAGAGAGGCTCATAAAAGAGTTCCTGAGGAAAGGCTTCAGGACAGTGTAGATCTTAAAAGAGCTAGGAAGGGAAAGCCTAAAAGAGAAGACCCTAAAGGCATTCCTGACTATTGGCTGATTGTTTTAAAGAATGTTGACAAGCTCGGGCCTATGATTCAGAAGTATGATGAGCCCATTCTGAAGTTCTTGTCGGATGTTAGCCTGAAGTTCTCAAAACCTGGCCAGCCTGTAAGTTACACCTTTGAATTTCATTTTCTACCCAACCCATACTTCAGAAATGAGGTGCTGGTGAAGACATATATAATAAAGTCAAAACCAGATCACAATGATCCCTTCTTTTCTTGGGGATGGGAAATTGAAGATTGCAAAGGCTGCAAGATAGACTGGAGAAGAGGAAAAGATGTTACTGTGACAACTACCCAGAGTCGCACAACTGCTACTGGAGAAATTGAAATCCAGCCAAGAGTGGTTCCTAATGCATCATTCTTCAACTTCTTTAGTCCTCCTGAGATTCCTATGATTGGGAAGCTGGAACCACGAGAAGATGCTATCCTGGATGAGGACTTTGAAATTGGGCAGATTTTACATGATAATGTCATCCTGAAATCAATCTATTACTATACTGGAGAAGTCAATGGTACCTACTATCAATTTGGCAAGCATTATGGAAACAAGAAATACAGAAAATAA

>*C. l. familiaris Napl13*

ATGGCAGAAGAGGATCTTAACATGGTCTCGGAACCTGCCGCCCAAAGGGTTACTGAAGAGAAGATGGCTAGCTCGTCTAGTGATTCTGGGGAAGAATCGGACAGCAATAGCTCTAGCAGCAGCACTAGTTGCAGCAGCAGCAGCGGCAGTGGCCGCAGCCGCTTATATAGAAAGAAGAGGGTATCTGGGCCTTCCAGAGGAGCACGGGGGGCTCCGCTGGGTAAAAGTTTTGTGGATCGGCTGCCTCAGGCAGTTAGAAATCGTGTGCAGGCTCTCAGAAATATTCAAGATGAATGTGACAAGGTTGACATCCTGTTCTTAAAGGCAATTCACGATCTCGAAAGAAAATATGCCGAACTCAATAAGCCTCTATACGATCGGCGATTTCAAATAATCAATGCAGAATATGAACCTACAGAAGAAGAATATGAATGGAATTCAGAGGATGAGGTGTTCAGCAGTGATGAGGAGGTGCAGGAAGACAGCCCTATTGAAATGCCTGCCTTAGAAAGTGAGCAGGAAGATGATAACCCTAAGGAAAAACCTCAGGTAAAGGCTGAAGAAAACGAGGTCCCAAAAGAAATTCCTGAGGCAAAGACTGAAGAAAACGCAGAATCTAAAGATTTTCTGGGTATAAAGCCTGAAGTGAAAGAAGAGCCTGAAGTAGTCCCCCAGCAAAATGCAGAAGATCAAAAACAGCCTAAAGCAGCAGAGGCTAAGGCAAAGGCTGCTGTAATAGAGGCTCCTAAAAGAATTTCTGAGGTCAGGCCTAAAGAAAGAGTAAATCTTAAAAGAGCTCGTAAGGGAAAGCCTAAAAAAGAAGATCCTAAAGGCATTCCTGACTATTGGCTGACTGTTTTAAAGAATGTCGACAAGCTTGGGCCCATGATTCAGAAGTATGATGCCCCCATTTTGAAGTTCTTGTCAGATATTAGCCTAAAGTTCTCAAAACCTGGCCAGCCTATAAGCTACACGTTTGAATTTTATTTTCTACCTAATCCATACTTCAGAAATGAGATGCTGACCAAGACATATATAATAAAGTCAAAACCAGATCACAACGATCCCTTCTTCTCTTGGGGATGGGAAATCCAAGATTGCAAAGGCTGTAAAATCGATTGGAGAAGAGGAAAGGATGTTACCGTGACAACCACCCAGAGCCGTACAACTGCTAGTGGAGAAATTGAAATTCAGCCAAGAGTGGTTGCTAATGCATCATTCTTCAATTTCTTTAGCCCTCCTGAGATTCCTAAGATTGGAAAGCTAGAACCACGAGAAGATGCCATCCTGGATGAAGATTTTGAAATTGGTCAAATTTTACATGATAACGTCATCCTGAAATCAATCTATTACTATACAGGAGAAGTCAAAGGTACCTATGATGATGGTAAAGATTATGGAAACAGGAAGTATCGAAAATAA

>*P. troglodytes Nap1l3*

atggccgagtcagagaaccacaaggagctgtcagaatccagtcaagaagaggctggtaatcagataatggtggaagggctcggggaacatctggagcgcggtgaagatgccgctgctgggcttggagacgatgggaagtgcggtgaagaagccgccgctgggcttggggaagaaggggaaaacggtgaagatactgctgctgggtccggggaagatgggaaaaaaggtggcgatactgatgaggactcagaggcagaccgtccaaaaggacttatcggttatgttttagatacagactttgttgaaagtctacctgtgaaagttaagtaccgtgtgttagcccttaaaaagcttcaaactagagcggccaatttagaatccaaattcctgagggaatttcatgacattgaaagaaagtttgctgaaatgtaccaacccttactggaaaaaagacgtcagatcatcaatgcaatctatgaacctacagaagaggaatgtgaatataaatcagactctgaggactgtgatgatgaggaaatgtgtcatgaagagatgtatggtaatgaggagggtatggtacatgaatatgtggatgaggacgatggttatgaggactattattatgattatgctgtggaagaggaggaggaggaggaggaggaggaggacattgaggctactggagaagagaataaagaagaggaggatcctaagggaattcctgatttttggctgactgttttaaaaaacgttgatacactcactcctttgattaagaaatatgatgagcctattctgaagctcctgacagatattaaagttaagctttcagatcctggcgagcccctcagtttcacactagaatttcacttcaaacccaatgaatatttcaaaaatgagttgttgacaaagacctatgtgctgaagtcaaagctagcatattatgatccccatccctataggggaactgcgattgagtattccacaggctgtgagatagattggaatgaaggaaagaatgtcactttgaaaaccatcaagaagaaacagaaacatcggatctggggaacaatccgaactgtaactgaagattttcccaaggattcatttttcaattttttctctcctcatggaatcacctcaaatggaagggatggaaatgatgattttttacttggtcacaatttacgtacttacataattccaagatcagtattatttttctcaggtgatgcactggaatctcagcaggagggggtagttagagaagttaatgatgcaatttatgacaaaattatttatgataattggatggctgcaattgaggaagttaaagcttgttgcaaaaaccttgaggcattagtagaagacattgatcgttag

>*C .l. familiaris Nap1l2*

ATGGCCGAGTCAGCCGACCACAAGAAACTGTTAGAATTTAGTCAAGAAGAGGCTGATAATAAGGTAATTATGGAGGGACCCGGGGAACAGCCGGAGCAGAGTGAAGATGTCGCAGCTGGGCCTGGAGATGATAAGGAGCGCGGTGAAGAAGCCGCCGTTGGGCCTGGGAAAGAAGGGGGAAAAGGAGAAGATGCTGCTGCTGGGTCCGGGGAAGGTGGGGTAAAAGATGAAGATATTGATGAGGATTCAGATCGTCCAAAAGGACTTATTGGTTATCTTTTAGATACAGACTTTGTTGAAAGTCTACCTTTGAAAGTTAAGTACCGTGTGTTAGCCCTCAAAAAGCTTCAAACTAGAGTGGCCAATCTAGAATCCAAATTTATGAGGGAATTTCATGGCATTGAAAGAAAGTTTGCTGAAATGTATCAACCCTTATTGGAAAAAAGACGTCAGATTATAAATGCAATCTATGAACCTACAAAAGAGGAATGTGAATATAAATCAGACTCAGAGGACTATGATGATGAGATGTATGATGAGGAAGAGATGTATGGTAATGAGGAGGGTCTGGTGCATGAGTATATGGATGAGGATGATGGTTATGAGGGAGATTATTATGATTATGCTGTTGAGGAAGATGATGGCGATGATGATGATGATGATAATGGTGATGACATTGAGGCTACTGGAAAAGAGAATAAAGAAGAACAGGATCCTAAAGGAATTCCTGATTTTTGGCTGACTGTCTTAAAAAATGTTGACACACTCACTCCTTTGATTAAGAAATATGATGAGCCTATTCTGAAGCTCCTGACAGATATTAAAGTGAAACTTTCAGGTCCTGGTGAGCCTCTCAGTTTCACACTAGAATTTCACTTCAAGCCCAATGAATATTTCAAAAATGAGCTGTTAACAAAGACTTATGTGCTGAAGTCAAGGCTGGCATATTATGATCCCCATCCCTATAGGGGAACTGCAATTGAGTATTGCACAGGCTGTGAGATAGATTGGAATGAAGGAAAGAATGTCACTTTGAAAACCATCAGGAAGAAGCAGAAGCATCGGATTTGGGGAACAGTACGAACTGTAACTGAAGATTTTCCCAAGGATTCATTCTTCAATTTCTTTACTCCTCAGGGAATCAGCTCAAATGGAAAAGATGGAAATGATGATTTTTTACTTGGTCACAATTTACGTACTTATATAATTCCAAGATCAGTGTTGTTTTTCTCAGGTGATGCCCTTGAGTCTCAGCAGGAGGGGGTAGTTAGGGAAGTTAATGATGCAATTTATGACAAAATTATTTATGATAATTGGATGGCTGCAATTGAGGAGGTTAAAGCCTGTTGCAAAAATCTTGAGACAATAGTAGAAGACATTGATCGCTAA

>*E. caballus Nap1l2*

ATGGCCGAGTCAGCCGACTACAAGGAACTGTTAGAATCCAGTCAAGAAGAGGCTGGTAATAAGGTAATGATGGAGGGACCCAGGGAACAGCCAGAGCGCGGTGAAGAAGCCGCCGCTGGGCCTGGGGAAGAAGGGGAAAGAGGTGAAGATGCTGCTGCTGGGTCCGGGGAAGGCGGGGAAAAAGGTGAAGATACTGATGAAGACTCAGACCCAGACCGTCCAAAAGGACTTACCGGTTATCTTTTAGATACTGACTTTGTTGAAAGTCTACCTGTGAAAGTTAAGTACCGTGTGTTAGCCCTCAAAAAGCTTCAGACTAGAGTGGCCAATCTAGAATCCAAATTCCTAAGGGAATTTCATGACATTGAAAGAAAGTTTGCTGAACTGTATCAACCCTTATTGGAAAAAAGACGTCAGATTATCAATGCAATCTATGAACCTACAAAAGAGGAATGTGCATATAAATCAGACTCTGAGGATTATGATGAGGAAGAGATGTATGATGAGGAAGAGATGTATGGTAATGAGGAAAGTCTTGTACATGAGTACATGGATGAGGGTGATGGTTATGAGGAAGATTATTATGATTATGATGTTGAGGAGGAGGAGGATGACGAAGACGATGACGATGATGATGATGATGACATCGAGGCTACCAGAGAAGAGAATAAAGAAGAGGATCCTAAAGGAATTCCTGATTTTTGGCTGACTGTCTTAAAAAACGTTGACACACTCACTCCTTTGATTAAGAAATATGATGAGCCTATTCTGAAGCTCCTGACAGATATTAAAGTGAAGCTTTCAGATCCTGGTGAGCCTCTCAGTTTCACTCTAGAATTTCACTTCAAGCCCAATGAATATTTCAAAAATGAGCTGTTGACAAAGACCTATGTGCTGAAGTCACGGCTAGCATTTTATGATCCCCATCCCTATAGAGGAACCGCAATTGAGTATTGCACAGGCTGTGAGATAGACTGGAATGAGGGAAAGAACGTCACTTTGAAAACCATCAAGAAGAAGCAGAAACACCGGATCTGGGGAACAATCCGAACTGTGACTGAAGATTTTCCCAAGGATTCATTCTTCAATTTCTTTACTCCTCACGGAATCAGCTCAAATGGAAAGGATGGAAATGATGATTTTTTACTTGGTCACAATTTACGTACTTACATAATCCCAAGATCAGTATTATTTTTCTCAGGTGATGCACTGGAATCTCAGCAGGAGGGAGTAGTTAGGGAAGTTAATGATGCAATTTATGACAAAATTATTTACGATAATTGGATGGCTGCAATTGAGGAGGTTAAAGCCTGTTGCAAAAATCTTGAGACAATAGTAGAAGACATTGATCGCTAA

>*H. sapiens NAP1L2*

ATGGCCGAGTCAGAGAACCGCAAGGAGCTGTCAGAATCCAGTCAAGAAGAGGCTGGTAATCAGATAATGGTGGAAGGGCTCGGGGAACATCTGGAGCGCGGTGAAGATGCCGCTGCTGGGCTTGGAGACGATGGGAAGTGCGGTGAAGAAGCTGCCGCTGGGCTTGGGGAAGAAGGGGAAAACGGTGAAGATACTGCTGCTGGGTCCGGGGAAGATGGGAAAAAAGGTGGCGATACTGATGAGGACTCAGAGGCAGACCGTCCAAAAGGACTTATCGGTTATGTTTTAGATACAGACTTTGTTGAAAGTCTACCTGTGAAAGTTAAGTACCGTGTGTTAGCCCTTAAAAAGCTTCAAACTAGAGCGGCCAATTTAGAATCCAAATTCCTGAGGGAATTTCATGACATTGAAAGAAAGTTTGCTGAAATGTACCAACCCTTACTGGAAAAAAGACGTCAGATCATCAATGCAATCTATGAACCTACAGAAGAGGAATGTGAATATAAATCAGACTCTGAGGACTGTGATGATGAGGAAATGTGTCATGAAGAGATGTATGGTAATGAGGAGGGTATGGTACATGAATATGTGGATGAGGACGATGGTTATGAGGACTATTATTATGATTATGCTGTGGAAGAGGAGGAGGAGGAGGAGGAGGAGGACGACATTGAGGCTACTGGAGAAGAGAATAAAGAAGAGGAGGATCCTAAGGGAATTCCTGATTTTTGGCTAACTGTTTTAAAAAACGTTGATACACTCACTCCTTTGATTAAGAAATATGATGAGCCTATTCTGAAGCTCCTGACAGATATTAAAGTTAAGCTTTCAGATCCTGGCGAGCCCCTCAGTTTCACACTAGAATTTCACTTCAAACCCAATGAATATTTCAAAAATGAGTTGTTGACAAAGACCTATGTGCTGAAGTCAAAGCTAGCATATTATGATCCCCATCCCTATAGGGGAACTGCGATTGAGTATTCCACAGGCTGTGAGATAGATTGGAATGAAGGAAAGAATGTCACTTTGAAAACCATCAAGAAGAAACAGAAACATCGGATCTGGGGAACAATCCGAACTGTAACTGAAGATTTTCCCAAGGATTCATTTTTCAATTTTTTCTCTCCTCATGGAATCACCTCAAATGGAAGGGATGGAAATGATGATTTTTTACTTGGTCACAATTTACGTACTTACATAATTCCAAGATCAGTATTATTTTTCTCAGGTGATGCACTGGAATCTCAGCAGGAGGGGGTAGTTAGAGAAGTTAATGATGCAATTTATGACAAAATTATTTATGATAATTGGATGGCTGCAATTGAGGAAGTTAAAGCTTGTTGCAAAAACCTTGAGGCATTAGTAGAAGACATTGATCGTTAG

>*C. jacchus Nap1l2*

ATGGCCGAGTCAGCGGACCATAAGGAACTGTCAGAATCCAGTCAAGAAGAGGCTGGTAATCAGATAATGATGGAAGGGCCCGGGGAACAACCGGAGCGCGGTGAAGATGCCGCTGCTGGGCTTGGAGATGATGGGAAGTGCGGCGATGAAGCCGCCGCTGGGCTCGGGGAAGAAGGGGAAAAAGGTGAAGATACTGCTGCTGGGTCTGGGGAAGATGGGAAAAAAGGTGGAGATACTGATGAGGACTTAGACCCAGACCGTCCAAAAGGACTTATCGGTTATGTTTTAGATACAGACTTTGTTGAAAGTCTGCCTGTGAAAGTTAAGTACCGTGTGTTGGCCCTTAAAAAGCTTCAAACTAGAGCGGCCAATTTAGAATCCAAATTCCTGAGGGAATTTCATGACATTGAAAGAAAGTTTGCTGAAATGTATCAGCCCTTACTGGAAAAAAGACGTCAGATCATCAATGCAATCTATGAACCTACAGAAGAGGAATGTGAATATAAATCAGACTCTGAGGACTATGACGATGAGGAAATGTATGATGAAGAGATGTATGGTAATGAGGAGGGAATGGTACATGAGTATGTGGATGAGGACGATGGCTATGAGGACTATTATTATGATTATGCTGTGGAGGAGGAGGAGGATGACGATGATGATGATGATGATGACATTGAGGCTACTGGAGAAGAGAATAAGGAAGAGGAGGATCCGAAGGGAATTCCTGATTTTTGGCTGACTGTTTTAAAAAACGTTGATGCACTCACTCCTTTGATTAAGAAATATGATGAGCCTATTCTGAAGCTCCTGACAGATATTAAAGTTAAGCTTTCAGATCCTGGCGAGCCTCTCAGTTTCACACTAGAATTTCACTTCAAACCCAATGAATATTTCAAAAATGAGTTGTTGACAAAGACCTATGTGCTGAAGTCAAAGCTAGCATATTATGATCCCCATCCCTATAGGGGAACTGCGATTGAGTATTCCACAGGCTGTGAGATAGATTGGAATGAAGGAAAGAATGTCACTTTAAAAACGATAAAGAAGAGACAGAAACATCGGATCTGGGGAACAATCCGAACTGTAACTGAAGATTTTCCCAAGGATTCATTTTTCAATTTTTTCTCTCCTCATGGATTCACCTCAAATGGAAGGGATGGAAATGATGATTTTCTACTTGGTCACAATTTACGTACTTACATAATTCCAAGATCAGTATTATTTTTCTCAGGTGATGCACTTGAATCACAGCAGGAGGGGGTAGTTAGAGAAGTTAATGATGAAATTTATGACAAAATTATTTATGATAATTGGATGGCTGCAATTGAGGAAGTTAAAGCTTGTTGCAAAAACCTTGAGGCATTAGTAGAAGACATTGACCGTTAA

>*M. musculus Nap1l2*

ATGGCTGAATCAGTCGACCATAAAGAACTGTCTGAATCCAACCAAGAAGAGCTTGGCAGCCAGGTAATGGCGGAGGGGCCCGGGGAAAGTCAGGACCGCAGTGAAGGTGTCTCCATTGAGCCTGGAGATGGCGGGCAACATGGTGAAGAAACCGTGGCTGCTGGAGTAGGGGAAGAGGGAAAAGGTGAAGAAGCTGCTGCAGGGTCTGGGGAAGATGCTGGGAAGTGCGGAGGCACTGATGAGGACTCAGACTCAGACCGTCCAAAAGGACTTATCGGTTATCTTTTAGATACCGATTTCGTTGAAAGTCTCCCAGTGAAAGTTAAGTGCCGAGTGCTAGCTCTTAAAAAGCTTCAAACAAGAGCTGCCCATTTGGAATCCAAATTCCTGAGGGAATTTCATGACATTGAAAGGAAGTTTGCTGAAATGTACCAACCCTTACTAGAAAAAAGACGACAGATCATCAATGCAGTCTATGAGCCCACAGAAGAGGAATGTGAGTATAAATCGGACTGTGAGGACTATTTTGAGGAGGAGATGGATGAGGAGGAAGAGACTAACGGCAACGAAGACGGTATGGTGCATGAATACGTGGATGAAGATGATGGTTATGAGGACTGTTATTATGATTATGATGACGAGGAAGAAGAGGAGGAGGAAGATGACAGCGCTGGGGCCACCGGAGGAGAAGAGGTTAACGAAGAGGATCCTAAGGGGATTCCGGATTTTTGGTTGACTGTTTTAAAAAATGTTGAAGCACTCACTCCTATGATTAAGAAATATGATGAGCCTATTCTGAAGCTGCTGACAGATATTAAAGTGAAGCTTTCGGATCCCGGGGAGCCTCTCAGCTTCACACTCGAATTTCACTTCAAGCCCAATGAATATTTTAAAAATGAGCTGTTGACAAAGACTTATGTGCTGAAGTCAAAGCTTGCATGCTACGATCCCCACCCTTATAGGGGAACTGCCATTGAGTACGCCACTGGCTGCGACATAGATTGGAACGAAGGGAAGAATGTCACTTTGAGAACCATCAAGAAGAAGCAGAGACATCGCGTCTGGGGAACTGTCCGAACTGTGACTGAAGATTTTCCCAAGGACTCTTTCTTCAATTTCTTCTCTCCTCATGGGATCAGCTTAAATGGAGGGGATGAAAATGATGATTTTTTACTTGGTCATAATCTGCGTACTTACATAATTCCAAGATCAGTGTTATTTTTCTCAGGAGATGCACTTGAATCTCAGCAGGAGGGTGTAGTTAGGGAAGTTAATGACGAAATATATGACAAAATTATTTATGATGATTGGATGGCTGCAATTGAAGAGGTTAAAGCCTGTTGCAAAAATCTTGAGGCATTAGTAGAAGATATTGATCGTTAA

>*R. novergicus Nap1l2*

ATGGCTGAGTCGGCCGAGCATAAGGAACTGTCTGACTCCAACCAAGAGGAGCTTGGTAGCCAGGTAATGGCGGAGGGGCCTGGGGGAAGTCAGGACTGCAGTGAAGGTGTCTCCACTGAGCCCGGAGATGGCGGGCAACAGGGTGAAGAAACCGTGGCTGCTGGAGTCGAGGAGGAGGGAAAAGGTGAAGAAGCTGCTGCAGGGTCTGGGGAAGATGCAGGCAAACGCGGAGGTGCTGCTGAGGACTCAGACTCAGACCGTCCAAAAGGACTTATCGGTTATCTTTTAGATACCGACTTTGTTGAAAGTCTCCCAGTGAAAGTTAAGTACCGAGTGCTGGCTCTTAAAAAGCTTCAAACAAGAGCTGCCCATTTGGAATCCAAATTCCTGAGGGAATTTCATGACATTGAAAGGAAGTTTGCTGAAATGTATCAACCCTTACTAGAAAAAAGACGACAGATCATCAATGCAGTCTATGAACCCACAGAAGAGGAATGTGAGTATAAATCAGACTGTGAGGACTATTTTGAAGAGGAGATGGATGAGGAGGAAGAGACTAACGGCAATGAGGAGGGCATGGTGCATGAATATGTGGATGAAGATGATGGTTATGAGGACTGTTATTATGATTATGATGACGAAGAAGAAGAGGAGGAGGAGGAAGATGAGAGCGCTGGGGCCACTGGAGGAGAAGCGGTTAACGAAGAGGATCCTAAGGGAATTCCTGATTTTTGGCTGACTGTTTTAAAAAATGTTGAAGCACTCACTCCTATGATTAAGAAGTATGATGAGCCTATTCTGAAGCTGCTGACAGATATTAAAGTGAAGCTTTCGGATCCTGGTGAGCCTCTCAGCTTTACACTCGAATTTCACTTCAAGCCCAATGAATATTTTAAAAATGAGCTGTTGACAAAGACTTATGTGCTGAAGTCAAAGCTTGCATGCTACGATCCCCACCCTTATAGGGGAACTGCCATCGAGTATGCCACTGGCTGCCACATAGAATGGAACGAAGGGAAGAATGTCACTTTGAGAACCATCAAGAAGAAACAGAGACATCGCGTCTGGGGAACTGTCCGAACCGTGACTGAAGATTTTCCCAAGGACTCTTTCTTCAATTTCTTCTCTCCTCATGGGATCAGCTTAAATGGAGGGGATGAAAATGATGATTTTTTACTTGGTCATAATTTGCGTACTTACATAATTCCAAGATCGGTGTTATTTTTCTCAGGAGATGCCCTTGAATCTCAGCAGGAGGGTGTAGTTAGGGAAGTTAATGATGAAATTTATGACAAAATTATTTATGATGATTGGATGGCTGCAATTGAGGAGGTTAAAGCCTGTTGCAAAAATCTTGAGGCATTAGTAGAAGATATTGATCGTTAA

>*M. mulatta Nap1l2*

ATGGCCGAGTCAGCGAACCACAAGGAGCTGTCAGAATCCAGTCAGCAAGAGGCTGGTAATCAGATAATGGTGGAAGGGCCCGGGGAACATCCGGAGCGCGGTGAAGATGCCGCTGCTGGGCTTGGAGATGATGGGAAGTGCGGTGAAGAAGCCGCAGCTGGGCTCGGGGAAGAAGGGGAAAATGGTGAAGATACTGCGGCTGGGTCCGGGGAAGATGGGAAAAAAGGTGAAGATACTGATAAGGACTCAGACCCAGACCGTCCAAAAGGACTTATCGGTTATGTTTTAGATACAGACTTTGTTGAAAGTCTACCTGTGAAAGTTAAGTACCGTGTGTTAGCCCTTAAAAAGCTTCAAACTAGAGCGGCCAATTTAGAATCCAAATTCCTGAGGGAATTTCATGACATTGAAAGAAAGTTTGCTGAAATGTACCAACCCTTACTGGAAAAAAGACGTCAGATCATCAATGCAATCTATGAACCTACAGAAGAGGAATGTGAATATAAATCAGACTCTGAGGACTATGATGATGAGGAAATGTGTGATGAAGAGATGTATGGTAATGAGGAGGGTATGGTACATGAGTATGTGGATGAGGATGATGGTTATGAGGACTATTATTATGATTATGCTGTTGAGGAGGAGGAGGAGGAGGAGGATGAGGATGAGGATGACATTGAGGCTACTGGAGAAGAGAATAAAGAAGAGGAGGATCCTAAGGGAATTCCTGATTTTTGGCTGACTGTTTTAAAAAACGTTGATACACTCACTCCTTTGATTAAGAAATATGATGAGCCTATTCTGAAGCTCCTGACAGATATTAAAGTTAAGTTTTCAGATCCTGGCGAGCCCCTCAGTTTCACACTAGAATTTCACTTCAAACCCAATGAATATTTCAAAAATGAGTTGTTGACAAAGACCTATGTACTGAAGTCAAAGCTAGCATATTATGATCCCCATCCCTATAGGGGAACTGCGATTGAGTATTCCACAGGCTGTGAGATAGATTGGAATGAAGGAAAGAATGTCACTTTGAAAACCATCAAGAAGAAACAGAAACATCGAATCTGGGGAACGATCCGAACTGTAACTGAAGATTTTCCCAAGGATTCATTTTTCAATTTTTTCTCTCCTCATGGAATCACCTCAAATGGAAGGGATGGAAATGATGATTTTTTACTTGGTCACAATTTACGTACTTACATAATTCCAAGATCAGTATTATTTTTCTCAGGTGATGCACTTGAATCTCAGCAGGAGGGGGTAGTTAGAGAAGTTAATGATGCAATTTATGACAAAATTATTTATGATAATTGGATGGCTGCAATTGAGGAAGTTAAAGCTTGTTGCAAAAACCTTGAGGCATTAGTTGAAGACATTGATCGTTAG

>*P. troglodytes Nap1l1*

ATGGCAGACATTGACAACAAAGAACAGTCTGAACTTGATCAAGATTTGGATGATGTTGAAGAAGTAGAAGAAGAGGAAACTGGTGAAGAAACAAAACTCAAAGCACGTCAGCTAACTGTTCAGATGATGCAAAATCCTCAGATTCTTGCAGCCCTTCAAGAAAGACTTGATGGTCTGGTAGAAACACCAACAGGATACATTGAAAGCCTGCCTAGGGTAGTTAAAAGACGAGTGAATGCTCTCAAAAACCTGCAAGTTAAATGTGCACAGATAGAAGCCAAATTCTATGAGGAAGTTCATGATCTTGAAAGGAAGTATGCTGTTCTCTATCAGCCTCTATTTGATAAGCGATTTGAAATTATTAATGCAATTTATGAACCTACGGAAGAAGAATGTGAATGGAAACCAGATGAAGAAGATGAGATTTCGGAGGAATTGAAAGAAAAGGCCAAGATTGAAGATGAGAAAAAGGATGAAGAAAAAGAAGACCCCAAAGGAATTCCTGAATTTTGGTTAACTGTTTTTAAGAATGTTGACTTGCTCAGTGATATGGTTCAGGAACACGATGAACCTATTCTGAAGCACTTGAAAGATATTAAAGTGAAGTTCTCAGATGCTGGCCAGCCTATGAGTTTTGTCTTAGAATTTCACTTTGAACCCAATGAATATTTTACAAATGAAGTGCTGACAAAGACATACAGGATGAGGTCAGAACCAGATGATTCTGATCCCTTTTCTTTTGATGGACCAGAAATTATGGGTTGTACAGGGTGCCAGATAGATTGGAAAAAAGGAAAGAATGTCACTTTGAAAACTATTAAGAAGAAGCAGAAACACAAGGGACGTGGGACAGTTCGTACTGTGACTAAAACAGTTTCCAATGACTCTTTCTTTAACTTTTTTGCCCCTCCTGAAGTTCCTGAGAGTGGAGATCTGGATGATGATGCTGAAGCTATCCTTGCTGCAGACTTCGAAATTGGTCACTTTTTACGTGAGCGTATAATCCCAAGATCAGTGTTATATTTTACTGGAGAAGCTATTGAAGATGATGATGATGATTATGATGAAGAAGGTGAAGAAGCGGATGAGGAAGGGGAAGAAGAAGGAGATGAGGAAAATGATCCAGACTATGACCCAAAGAAGGATCAAAACCCAGCAGAGTGCAAGCAGCAGTGA

>*H. sapiens NAP1L1*

ATGGCAGACATTGACAACAAAGAACAGTCTGAACTTGATCAAGATTTGGATGATGTTGAAGAAGTAGAAGAAGAGGAAACTGGTGAAGAAACAAAACTCAAAGCACGTCAGCTAACTGTTCAGATGATGCAAAATCCTCAGATTCTTGCAGCCCTTCAAGAAAGACTTGATGGTCTGGTAGAAACACCAACAGGATACATTGAAAGCCTGCCTAGGGTAGTTAAAAGACGAGTGAATGCTCTCAAAAACCTGCAAGTTAAATGTGCACAGATAGAAGCCAAATTCTATGAGGAAGTTCACGATCTTGAAAGGAAGTATGCTGTTCTCTATCAGCCTCTATTTGATAAGCGATTTGAAATTATTAATGCAATTTATGAACCTACGGAAGAAGAATGTGAATGGAAACCAGATGAAGAAGATGAGATTTCGGAGGAATTGAAAGAAAAGGCCAAGATTGAAGATGAGAAAAAAGATGAAGAAAAAGAAGACCCCAAAGGAATTCCTGAATTTTGGTTAACTGTTTTTAAGAATGTTGACTTGCTCAGTGATATGGTTCAGGAACACGATGAACCTATTCTGAAGCACTTGAAAGATATTAAAGTGAAGTTCTCAGATGCTGGCCAGCCTATGAGTTTTGTCTTAGAATTTCACTTTGAACCCAATGAATATTTTACAAATGAAGTGCTGACAAAGACATACAGGATGAGGTCAGAACCAGATGATTCTGATCCCTTTTCTTTTGATGGACCAGAAATTATGGGTTGTACAGGGTGCCAGATAGATTGGAAAAAAGGAAAGAATGTCACTTTGAAAACTATTAAGAAGAAGCAGAAACACAAGGGACGTGGGACAGTTCGTACTGTGACTAAAACAGTTTCCAATGACTCTTTCTTTAACTTTTTTGCCCCTCCTGAAGTTCCTGAGAGTGGAGATCTGGATGATGATGCTGAAGCTATCCTTGCTGCAGACTTCGAAATTGGTCACTTTTTACGTGAGCGTATAATCCCAAGATCAGTGTTATATTTTACTGGAGAAGCTATTGAAGATGATGATGATGATTATGATGAAGAAGGTGAAGAAGCGGATGAGGAAGGGGAAGAAGAAGGAGATGAGGAAAATGATCCAGACTATGACCCAAAGAAGGATCAAAACCCAGCAGAGTGCAAGCAGCAGTGA

>*P. troglodytes Nap1l4*

ATGGCAGATCACAGTTTTTCAGATGGGGTTCCTTCAGATTCCGTGGAAGCTGCTAAAAATGCAAGTAACACAGAAAAGCTCACAGATCAGGTGATGCAGAATCCTCGAGTTCTGGCAGCTTTACAGGAGCGACTTGACAATGTCCCTCACACCCCTTCCAGCTACATCGAAACTTTACCTAAAGCAGTAAAAAGAAGAATTAATGCATTGAAACAACTTCAGGTGAGATGTGCTCACATAGAAGCCAAGTTCTATGAAGAGGTACATGACTTGGAAAGAAAGTATGCAGCGCTATACCAGCCTCTCTTTGACAAGAGAAGAGAATTTATCACCGGCGATGTTGAACCAACAGATGCGGAATCGGAATGGCACAGTGAAAATGAAGAGGAAGAGAAATTGGCTGGAGACATGAAAAATAAAGTAGTCGTAACAGAAAAAGAAGCAGCAACAGCTGAAGAGCCAAATCCCAAAGGAATTCCAGAGTTCTGGTTTACCATCTTTAGAAATGTAGATATGCTAAGTGAATTAGTCCAGGAATATGATGAACCAATCTTGAAACACCTGCAGGATATTAAAGTGAAATTTTCTGACCCTGGACAGCCTATGTCTTTTGTGTTAGAGTTCCACTTTGAACCCAACGACTACTTTACCAACTCAGTCCTGACAAAAACCTACAAGATGAAATCAGAACCAGATAAGGCTGATCCCTTTTCCTTTGAAGGTCCTGAGATTGTGGACTGTGACGGGTGTACTATTGACTGGAAGAAAGGAAAGAATGTTACTGTCAAAACCATCAAGAAAAAGCAGAAGCATAAGGGTCGAGGCACTGTTAGAACAATTACGAAACAAGTACCCAATGAGTCCTTTTTCAACTTCTTCAATCCATTGAAAGGCAATTCATCTGGGGATGGAGAATCACTGGATGAAGATTCTGAATTCACATTAGCCTCTGATTTTGAAATTGGACACTTTTTCCGTGAGCGGATAGTCCCGCGGGCTGTGCTGTACTTCACTGGGGAGGCCATAGAAGATGATGACAATTTTGAAGAAGGTGAAGAAGGAGAAGAGGAGGAATTAGAAGGTGACGAGGAGGGAGAAGACGAGGATGATGCGGAAATTAACCCCAAGGTG

>*H. sapiens NAP1L4*

atggcagatcacagtttttcagatggggttccttcagattccgtggaagctgctaaaaatgcaagtaacacagaaaagctcacagatcaggtgatgcagaatcctcgagttctggcagctttacaggagcgacttgacaatgtccctcacaccccttccagctacatcgaaactttacctaaagcagtaaaaagaagaattaatgcattgaaacaacttcaggtgagatgtgctcacatagaagccaagttctatgaagaggtacatgacttggaaagaaagtatgcagcgctataccagcctctctttgacaagagaagagaatttatcaccggcgatgttgaaccaacagatgcggaatcggaatggcacagtgaaaatgaagaggaagagaaattggctggagacatgaaaagtaaagtagtcgtcacagaaaaagcagcggcaacggctgaagagccagatcccaaaggaattccagagttctggtttaccatcttcagaaatgtggacatgctgagtgaattagtccaggaatatgatgaaccaatcttgaaacacctgcaggatattaaagtgaaattttctgaccctggacagcctatgtcttttgtgttagagttccactttgaacccaacgactactttaccaactcagtcctgacaaaaacctacaagatgaaatcagaaccagataaggctgatcccttttcctttgaaggtcctgagattgtggactgtgacgggtgtactattgactggaagaaaggaaagaatgttactgtcaaaaccatcaagaaaaagcagaagcataagggtcgaggcactgttagaacaattacgaaacaagtacccaatgagtcctttttcaacttcttcaatccattgaaagcatccggggatggagaatcactggatgaagattctgaattcacattagcctctgattttgaaattggacactttttccgtgagcggatagtcccgcgggctgtgctgtacttcactggggaggccatagaagatgatgacaattttgaagaaggtgaagaaggagaagaggaggaattagaaggtgacgaggagggagaagacgaggatgatgcggaaattaaccccaaggtg

**U2af1-rs Family**

>*P. troglodytes U2af1-rs2*

ATGACGTTTCCCGAGAAACCAAGCCACAAAAAGTACAGGGCCGCCCTGAAGAAGGAGAAACGAAAGAAACGTCGGCAGGAACTTGCTCGACTGAGAGACTCAGGACTCTCACAGAAGGAGGAAGAGGAGGACACTTTTATTGAAGAACAACAACTAGAAGAAGAGAAGCTATTGGAAAGAGAGAGGCAAAGATTACATGAGGAGTGGTTGCTAAGAGAGCAGAAGGCACAAGAAGAATTCAGAATAAAGAAGGAGAAGGAAGAGGCGGCTAAAAAACGGCAAGAAGAACAAGAGAGAAAGTTAAAGGAACAATGGGAAGAACAGCAGAGGAAAGAGAGAGAAGAGGAGGAGCAGAAACAACAGGAGAAGAAAGAAAAAGAGGAAGCTTTGCAGAAGATGCTGGATCAGGCTGAGAATGAGTTGGAAAATGGTACCACATGGCAAAACCCAGAACCACCCGTGGATTTCAGAGTAATGGAGAAGGATCGAGCTAATTGTCCCTTCTACAGTAAAACAGGAGCTTGCAGATTTGGAGATAGATGTTCACGTAAACATAATTTCCCAACATCCAGTCCTACCCTTCTTATTAAGAGCATGTTTACGACGTTTGGAATGGAGCAGTGCAGGAGGGATGACTATGACCCTGACGCAAGCCTGGAGTACAGCGAGGAAGAAACCTACCAACAGTTCCTAGACTTCTATGAGGATGTGTTGCCCGAGTTCAAGAACGTGGGGAAAGTGATTCAGTTCAAGGTCAGCTGCAATTTGGAACCTCACTTGAGGGGCAATGTATATGTTCAGTACCAGTCGGAAGAAGAATGCCAAGCAGCCCTTTCTCTGTTTAACGGACGATGGTATGCAGGACGACAGCTGCAGTGTGAATTCTGCCCCGTGACCCGGTGGAAAATGGCGATTTGTGGTTTATTTGAAATACAACAATGTCCAAGAGGAAAGCACTGCAACTTTCTTCATGTGTTCAGAAATCCCAACAATGAATTCTGGGAAGCTAATAGAGACATCTACTTGTCTCCAGATCGGACTGGCTCCTCCTTTGGGAAGAACTCCGAAAGGAGGGAGAGGATGGGCCACCACGACGACTACTACAGCAGGCTGCGGGGAAGGAGAAACCCTAGTCCAGACCACTCCTACAAAAGAAATGGGGAATCCGAGAGGAAAAGGAGTAGTCACAGGGGGAAGAAATCTCACAAACGCACATCAAAGAGTCGGGAGAGGCACAATTCACGAAGCAGAGGAAGAAATAGGGACCGCAGCAGGGACCGCAGCCGGGGCCGGGGCAGCCGGAGCCGGAGCCGCAGGAGCCGCCGCAGCCGGAGCCAAAGTTCCTCTAGGTCCCGAAGTCGTGGCAGGAGGAGGTCGGGTAATAGAGACAGAACTGTTCAGAGTCCCAAATCCAAATAA

>*C. l. familiaris U2af1-rs2*

ATGGCGGCTCCCGAGAAGATGATGTTTCCGGAGAAACCAAGCCACAAAAAGTACAGGGCTGCCCTGAAAAAGGCGAAACGAAAGAAACGACGGCAGGAACTGGCTCGATTGAGAGACTCAGGACTCTTACAGAAGGAGGAAGAGGATGCTTTTATTGAAGAACAACGCCTAGAAGAAGAGAAGCTGTTGGAGATAGAGAGGCAAAAATTACATGAAGCGTGGTTGCTTCGGGAGCAGAAGGCACAAGAAGAATTCAGAAGAAAGAAGGAAAAGGAAGAGGCGGCTAGAAAACGGCAGGAAGAACAAGAGAGAAAGTTAAAGGAAGAGTGGGAAGAGCAGCAGAGAAGAGAGAGAGAAGAGGAGGAGCAGAAGCTGCAGGAGAAGAGAGAAAGAGAGTTGGAAAATGGTGCCACATGGCAAAACCCAGAACCACCCATCGACTTAAGGATAATGGAGAAAGATCGAGCTAATTGTCCATTCTACAGTAAAACAGGAGCATGCAGATTTGGAGATAGGTGTTCACGTAAACACAATTTCCCATCTTCGAGCCCCACACTTCTTATTAAAAGCATGTTTACAACGTTTGGGATGGAGCAGTGCAGAAGGGATGACTATGACCCAGACGCGAGCCTAGAGTACAGTGAAGAGGAAACCTACCAGCAGTTCCTGGACTTCTATGATGACGTGCTTCCGGAGTTCAAGAACGTGGGGAAAGTGATCCAGTTCAAGGTCAGCTGCAACTTGGAACCTCATCTGAGGGGCAATGTGTATGTTCAGTATCAATCGGAAGAAGAATGCCAGGCAGCCCTCTCTCTGTTTAACGGACGATGGTATGCAGGACGGCAGCTTCAGTGCGAATTCTGTCCAGTGACCCGATGGAAAATGGCAATTTGTGGTTTATTTGAAATACACCAGTGCCCAAGAGGAAAACACTGCAACTTTCTTCATGTGTTCAGAAATCCCAACAATGAGTTTTGGGAAGCTAACAGAGACATCTACCTGTCTCCCGATCGGACCGGCTCATCCTTTGGGAAGGGCTCGGAGAGGAGAGAGAGGACGGGTCACCACGATGAGTACTATGGGAGGCCACGGCGGCGAAGAAGCCCCAGCCCGGTCCACTCCTACAAGAGAAACGGGGAAGCCGAGAGGAAGAGGAGGAGCAGCCACCGGGGGAAGAAGTCTCACAAACACCTGTCGAGGAGCCGCGACGGGCCCAGTTCACGGAGCCGAGGCAGGAAGAGGGGCCGCAGCCGTGGCCGGGGCAGCCGGAGCCAGAGCTCCTCTAGGTCCAGGAGTCGAGGCAGGAGGAGGTCAGGCAGCAGAGACTGA

>*H. sapiens U2AF1-RS2*

ATGGCTGCGCCCGAGAAGATGACGTTTCCCGAGAAACCAAGCCACAAAAAGTACAGGGCCGCCCTGAAGAAGGAGAAACGAAAGAAACGTCGGCAGGAACTTGCTCGACTGAGAGACTCAGGACTCTCACAGAAGGAGGAAGAGGAGGACACTTTTATTGAAGAACAACAACTAGAAGAAGAGAAGCTATTGGAAAGAGAGAGGCAAAGATTACATGAGGAGTGGTTGCTAAGAGAGCAGAAGGCACAAGAAGAATTCAGAATAAAGAAGGAAAAGGAAGAGGCGGCTAAAAAACGGCAAGAAGAACAAGAGAGAAAGTTAAAGGAACAATGGGAAGAACAGCAGAGGAAAGAGAGAGAAGAGGAGGAGCAGAAACGACAGGAGAAGAAAGAAAAAGAGGAAGCTTTGCAGAAGATGCTGGATCAGGCTGAAAATGAGTTGGAAAATGGTACCACATGGCAAAACCCAGAACCACCCGTGGATTTCAGAGTAATGGAGAAGGATCGAGCTAATTGTCCCTTCTACAGTAAAACAGGAGCTTGCAGATTTGGAGATAGATGTTCACGTAAACATAATTTCCCAACATCCAGTCCTACCCTTCTTATTAAGAGCATGTTTACGACGTTTGGAATGGAGCAGTGCAGGAGGGATGACTATGACCCTGACGCAAGCCTGGAGTACAGCGAGGAAGAAACCTACCAACAGTTCCTAGACTTCTATGAGGATGTGTTGCCCGAGTTCAAGAACGTGGGGAAAGTGATTCAGTTCAAGGTCAGCTGCAATTTGGAACCTCACCTGAGGGGCAATGTATATGTTCAGTACCAGTCGGAAGAAGAATGCCAAGCAGCCCTTTCTCTGTTTAACGGACGATGGTATGCAGGACGACAGCTGCAGTGTGAATTCTGCCCCGTGACCCGGTGGAAAATGGCGATTTGTGGTTTATTTGAAATACAACAATGTCCAAGAGGAAAGCACTGCAACTTTCTTCATGTGTTCAGAAATCCCAACAATGAATTCTGGGAAGCTAATAGAGACATCTACTTGTCTCCAGATCGGACTGGCTCCTCCTTTGGGAAGAACTCCGAAAGGAGGGAGAGGATGGGCCACCACGACGACTACTACAGCAGGCTGCGGGGAAGGAGAAACCCTAGTCCAGACCACTCCTACAAAAGAAATGGGGAATCCGAGAGGAAAAGTAGTCGTCACAGGGGGAAGAAATCTCACAAACGCACATCAAAGAGTCGGGAGAGGCACAATTCACGAAGCAGAGGAAGAAATAGGGACCGCAGCAGGGACCGCAGCCGGGGCCGGGGCAGCCGGAGCCGGAGCCGGAGCCGGAGCCGCAGGAGCCGCCGCAGCCGGAGCCAAAGTTCCTCTAGGTCCCGAAGTCGTGGCAGGAGGAGGTCGGGTAATAGAGACAGAACTGTTCAGAGTCCCAAATCCAAATAA

>*C. jacchus U2af1-rs2*

ATGGCTGCGCCCGAGAAGATGACGTTTCCAGAGAAACCAAGCCACAAAAAGTACAGGGCCGCCCTGAAGAAGGAGAAACGAAAGAAACGTCGGCAGGAACTTGCTCGACTGAGAGACTCAGGACTCTCACAGAAGGAGGAGGAGGAGGACACTTTCATTGAAGAACAACAACTTGAAGAAGAGAAGCTATTGGAAAGAGAGAGGCAAAGATTACATGAAGAGTGGTTGCTGAGAGAGCAGAAGGCACAAGAAGAATTCAGAATAAAGAAGGAAAAGGAAGAGGCAGCTAGAAAACGGCAAGAAGAACAAGAGAGAAAGTTAAAGGAGCAATGGGAAGAACAACAAAGGAAAGAGAGAGAAGAGGAGGAGCAGAAACGGCAGGAGAAGAGAGAAAAAGAGGAAGCTGTGCAGAAGATGCTGGATCGGGCTGAAAATGAGTTGGAAAATGGTACCACATGGCAAAACCCAGAACCACCCGTGGATTTCAGAGTAATGGAGAAGGATCGAGCTAATTGTCCCTTCTACAGTAAAACAGGAGCTTGCAGATTTGGAGATAGATGTTCACGTAAACATAATTTCCCAACATCCAGTCCTACCCTTCTTATTAAGAGCATGTTTACAACATTTGGAATGGAGCAGTGCAGGAGGGATGACTATGACCCTGACGCAAGCCTGGAGTACAGCGAGGAAGAAACCTACCAACAGTTCCTAGATTTCTATGAGGATGTGCTGCCTGAGTTCAAGAACGTGGGGAAAGTGATTCAGTTCAAGGTCAGCTGCAATTTGGAACCTCACCTGAGGGGCAACGTATATGTTCAGTACCAGTCGGAAGAAGAATGCCAAGCAGCCCTTTCTCTGTTTAACGGACGATGGTATGCCGGACGACAGCTTCAGTGCGAATTCTGCCCCGTGACCCGGTGGAAAATGGCGATTTGTGGTTTATTTGAAATACAACAATGTCCAAGAGGAAAACACTGTAACTTTCTTCACGTGTTCAGAAATCCCAACAATGAATTCTGGGAAGCGAATAGAGACATCTACTTGTCTCCAGATCGGACTGGCTCCTCCTTTGGCAAGAACTCTGAGAGGAGGGAGAGGATGGGCCACCACGACGAATACTACAGCAGGCTGCGGGGAAGGAGAAACCCAAGTCCAGACCACTCCTACAAAAGAAATGGGGAATCCGAGAGGAAAAGGAGTAGTCACAGGGGGAAGAAATCTCACAAACGCACATCAAAGAGTCGGGAGAGGCATAGTTCACGCAGCAGAGGAAGAAATAGGGACCGCAGCCGGGGCCGGGGCAGCCGGAGTCAGAGCCGCAGAAGCCGCCGCAGCCGGAGCCAAAGTTCCTCTAGGTCCCGAAGTCGTGGGAGGAGGAGGTCAGGTAATAAAGACAGAACTGTTCAAAGTCCCAAATCCAAATAA

>*M. musculus U2af1-rs2*

ATGGAAACGGCGGGGGCTACGGCGGACGCTACGGCGGGGCCGCAGAAACTGAGCCGTAAGAAGTACTTGGCCCTCCGGAAGAAGGAGAGACGTAAGAGGCGACGGCAGGCGCTCGCTCGGCTCAGAGAGGCAGAATTGGCACAGAAAGAGGAGGAAGAAGATCCTCTTGCTGAAGAAAAACGACTAGAAGAAGAAAGGTTGTTAGAGGAAGAGAGGCAGAGACTGCATGAAGAATGGTTACTGAGGGAAGAAAAGGCACAAGAAGAATTCAGGGCAAAGAAGAAAAAAGAAGAGGAAGCTCGAAAACGGAAGGAAGAACTAGAGAGAAAGTTAAAGGCAGAATGGGAAGAACAGCAGAGAAAAGAGCGAGAGGAAGAGGAGCAGAAACGACAAGAGAAGAGAGAAAGAGAGGAAGCTGTGCAGAAGATGCTGGATCAGGCTGAAAATGAGCTGGAAAATGGTGGCACCTGGCAGAACCCAGAACCGCCCATGGATATAAGAGTACTGGAGAAAGATCGAGCCAACTGTCCGTTCTACAGCAAAACGGGAGCTTGCAGATTTGGAGATAGGTGTTCACGTAAACACAACTTCCCCACATCAAGTCCCACCTTGCTGATTAAGGGCATGTTTACAACATTTGGAATGGAGCAGTGTCGGAGGGATGACTATGACCCTGACTCAAGCCTAGAATTCAGTGAAGAAGAGATCTACCAACAGTTCCTAGACTTCTATTATGATGTCCTGCCTGAATTCAAGAGTGTGGGAAAAGTGATTCAGTTTAAGGTCAGCTGCAACCTGGAACCTCATCTGAGGGGCAATGTGTATGTTCAGTACCAGTCGGAAGAAGACTGTCAAGCAGCCTTTTCTGTTTTTAATGGACGATGGTATGCAGGACGACAGCTCCAGTGTGAATTCTGCCCAGTGACCCGGTGGAAAATGGCAATTTGTGGCTTATTTGAAGTCCAGCAGTGTCCAAGAGGAAAGCACTGCAACTTTCTTCACGTGTTCAGAAATCCCAACAATGAATATAGGGACGCTAATAGAGACCTCTACCCGTCTCCAGATTGGACTAGCTCCTCCTTCGGTAAGAATTCAGAGAGGAGGGAAAGGGCCAGTCACTATGATGAATACTATGGCAGGTCAAGGAGGAGGAGGAGGAGCCCGAGTCCCGACTTCTACAAGAGAAATGGCGAGTCTGACAGGAAGAGCAGTAGCAGACACAGGGTGAAGAAATCTCACAGATATGGAATGAAGAGTCGTGAGAGGCGCAGTTCACCCAGTAGAAGAAGAAAGGACCACAGTCCGGGCCCCTGGAGCCAGAGCAGGAGGAGCCACCGCAGCAGGAGTCGCAGTCGCAGCCGCAGCCGCAGCCGCAGTCGGACCCGTAGCCGAAGCCGGGGCCGGGGCCGGAGCAGGAGCCGGAGCCGGAGCAGGAGCAGGGGCCGGAGCAGGAGCAGGGGCCGGGGCAGCGGCAGGGGCAGGGGCAGGGGCAGGGGCAGGGGCAGGAACCAGAGCAGGAGTTGGAGTCAAAGCCGGAGCCGGAGTAGTAGCTCGTCAAGGTCCAGAAGTCGTGGCAGGAGGTCAGGTAGTAGAGACAAAACCACCCAGAGTCCCAAATCTAAATAA

>*M. domesticus U2af1-rs2*

ATGAAAGTTGATGATTTGCCTGAGAAAGATGAAGAAGAAGAAGAATCTATTGTTGAACTGGAAGAAGAGGAAAAACAGTTAGAAGCTGAAAGGCAAAAGTTGCATGAGGAGTGGTTGCTGAGGGAACAGAAGGCTCAAGAAGAATTTAAACTTAAGAAGGAAAAGGAAGAGGCAGCAAAAAGGCGTCAAGAAGAAGAAGAGAGAAAGATCAAAGAAGAATGGGAAGAACAGCAAAGGAAAGAGAAACAGGAAGAAGAACAGAGACAGCAGGAGAAGAGAGACAGAGAGGAAGCTGTGCAGAAGATGCTGGATCAAGCTGAAAGTCAGCTGGAAAATGGTGTCACATGGCATAATCCAGAACCACCAGTGGATTTAAGAGCAACAGAGAAAGATCAAGCTAATTGTCCATTCTACATTAAAACAGGATCTTGCCGATTTGGAGATAGGTGTTCACGTAAGCACAATTACCCAACATCCAGTCAAACTCTCCTTATTAGAAGTATGTTTATAACATTTGGAATGGAACAGTGCAGAAGAGATGACTACGATACTGATGCAAGTCTGGAGTACAGTGAGGAGGAAACCTATCAACAATTTTTAGATTTCTATGAAGATGTACTCCCAGAATTCAAGAATGTGGGGAAAGTTATTCAGTTCAAGGTCAGCTGTAACTTTGAACCTCATCTTAGAGGAAATGTATATGTGCAGTATCAGTCGGAGGAAGAATGTCAAGCAGCATTCTCTCTATTCAATGGACGGTGGTATGCAGGACGACAGCTTCAGTGTGAATTCTCACCAGTAACGCGGTGGAAAATGGCTATTTGTGGTTTATTTGGAAGACAAAAGTGTCCAAGGGGAAAGCACTGTAATTTTCTTCATGTATTCAGAAATCCCAATAATGAATTTTGGGAAGCTAATAGAGACATACACATGTCTCCAGATTGGGCTAATACATCATTTGGTAAAAGCTCAGAAAGGAGAGATAGGACAAGTTATCATGAAGAATATTACAGCAGATCAAGAAGGCGGCGAAGTCCCAGTCCAGATCATTCTTATAAAAGAAATGGGGAATCTGAGAGGAAGAAAAGTAATCATCACAGGAGTAAGAAAAAGTCCCATAAACATTCCTCCAAAAGTCACGAAAGGCGACGTTCACGAAGTAGAGGAAGAAAGAGAGATCGGAGCCGAAGAAGCCGGAGCCAGAGTAGAAGCCGAAGAAGCCAAAGCAAAAGTGCATCCAGGTCTAGGAGTCGGGACAGGAAGAGATCAACCAGCAGAGATCGAAATACTTAA

>*R. novergicus U2af1-rs2*

ATGGTGGCTGGAAGCCGTAAGAAGTACATGGCTCTCCGGAAGAAGGAGAAACGTAAGAAACGACGGCAGGAGCTTGCTCGTCTGAGAGATGCAGAACTGTCACAGAAAGAAGAGGAAGAAGATCCTCTTGGTGAAGAAAAACGATTAGAAGAAGAAAGGTTGTTGGAGGCAGAGAGGCAAAGATTACATGAAGAATGGTTGCTGAGGGAAGAGAAGGCACAAGAAGAATTCAGGGCAAAGAAGAGAAAAGAAGAGGCAGCTCGAAAACGGAAGGAAGAACTAGAGAGAAAGTTAAAGGCAGAATGGGAAGAACAACAGAGAAAAGAACGAGAGGAAGAGGAGCAGAAACAACAAGAGAAGAGAGAAAGAGAGTTGGAAAATGGTGGCACCTGGCAGAACCCAGAACCGCCCACGGATATAAGAGTACTGGAGAAAGATCGAGCCAACTGTCCATTCTACAGTAAAACAGGAGCTTGCAGATTTGGAGATAGGTGTTCACGTAAACACAACTTTCCCACATCAAGTCCCACCTTGCTGATTAAGAGCATGTTTACAACGTTTGGAATGGAGCAGTGCAGGAGGGATGACTATGACCCTGACTCAAGCCTAGAATACAGTGAAGAAGAGACCTACCAACAGTTCCTAGACTTCTATTATGATGTCCTGCCTGAGTTCAAGAGTGTGGGGAAAGTGATTCAGTTTAAGGTTAGCTGCAACTTGGAACCTCATCTGAGGGGCAATGTATATGTTCAGTACCAGTCGGAAGAAGACTGTCAAGCAGCTTTTTCTGTTTTTAATGGACGATGGTATGCAGGACGACAGCTTCAGTGTGAATTCTGCCCAGTGACCCGGTGGAAAATGGCAATTTGTGGCTTATTTGAAGTACAGCAGTGTCCAAGAGGAAAGCACTGCAACTTTCTTCACGTGTTCAGAAATCCCAACAATGAATATAGGGAAGCTAATAGAGACATCTACCTGTCTCCAGATTGGACCAGCTCCTCCTTTGCTAAGAGTTCAGAGAGGAGGGAAAGGGCCAGTCACTATGATGAATACTATGGCAGGTCAAGAAGAAGGAGGAGTCCGAGTCCGGGCCTCTCCTACAAGAGAAATGGTGAGTCTGACAGGAAGAGCAGTAGTAATCACAGGGTGAAGAAATCTCACAAACATGGAATGAAGAATCGTGAGAGGCACAGTTCTCGGAGTAGAAGAAGAAAAAGAGATCACAGTCTAGGCCCCTGGAGCCAGAGCAGGAGGAGCCGCCATAGCAGGAGCCGCAGTCACAGTCAGAGCCGCAGCAGGAGCAGCAGCCGGAGCCGCAGCTGTGGCCGTGGCCGTGGCTGGAGCCACAGCCGTAGCCACAGCAGAAGCCGGAAACGAAGCCGGAATCGGAGCCGGAGCCGGAGCCGGAACCGGAACCGGAACCGGAACCGGAAACGCAATCAGAGCAGGAGTTGGAGCCATAGCCATAGCCATAGCCGGAGCTCATCAAGGTCCAGAAGTCCTGGCAGGAGGAGGTCAGCTAACATGGACATGGGAGGCAGTGACCATCTACAGTGTGAAATGCCACAAGAAGTCATCAGCAAGGGAGGACAGTTATGTCAGTCCAATTTCTTCAAGAACACTACGTGGGGTTTCAGCTTCCTGTGGAGGGAAAATGTGAGACTCTGGTAA

>*M. mulatta U2af1-rs2*

ATGACGTTTCCAGAGAAACCAAGCCACAAAAAGTACAGGGCCGCCCTGAAGAAAGAGAAACGAAAGAAACGTCGGCAGGAACTTGCTCGACTGAGAGACTCAGGACTCTCACAGAAGGAGGAAGAGGAGGACACTTTTATTGAAGAACAACAACTAGAAGAGAAGCTATTGGAAAGAGAGAGGCAAAGATTACATGAGGAGTGGTTGCTAAGAGAGCAGAAGGCACAAGAAGAATTCAGAATAAAGAAGGAAAAGGAAGAGGCGGCTAAAAAACGGCAAGAAGAACAAGAGAGAAAGTTAAAGGAACAATGGGAAGAACAGCAGAGGAAAGAGAGAGAAGAGGAGGAGCAGAAACGACAGGAGAAGAAAGAAAAAGAGGAAGCTTTGCAGAAGATGCTGGATCAGGCTGAAAATGAGTTGGAAAATGGTACCACATGGCAAAACCCAGAACCACCCGTGGATTTCAGAGTCATGGAGAAGGATCGAGCCAACTGTCCCTTCTACAGTAAAACAGGAGCGTGCAGATTTGGAGATAGATGTTCACGTAAACATAATTTCCCAACATCCAGTCCTACCCTTCTTATTAAGAGCATGTTTACAACATTTGGAATGGAGCAGTGCAGAAGGGATGACTATGACCCTGATGCAAGCCTGGAGTACAGCGAGGAAGAAACCTACCAGCAGTTCCTAGATTTCTACGAGGATGTGTTGCCCGAGTTCAAGAACGTGGGGAAAGTGATTCAGTTCAAGGTCAGCTGCAATCTGGAGCCTCACCTGAGGGGCAATGTGTATGTTCAGTACCAGTCGGAAGAAGAATGCCAAGCAGCCCTTTCTCTGTTTAACGGGCGATGGTATGCGGGACGACAGCTGCAGTGTGAATTCTGCCCAGTGACCCGGTGGAAAATGGCGATTTGTGGGTTATTTGAAATACAACAGTGTCCAAGAGGAAAGCACTGCAACTTTCTTCATGTGTTCAGAAATCCCAACAATGAATTCTGGGAAGCTAATAGAGACATCTACTTGTCTCCAGATCGGACTGGCTCCTCCTTTGGTAAGAACTCCGAAAGGAGGGAGAGGATGGGTCACCACGACGACTACTACAGCAGGCTGCGGGGAAGGAGAAACCCTAGTCCAGACCACTCCTACAAAAGAAATGGGGAATCCGAGAGGAAAAGGAGTAGTCACAGGGGGAAGAAATCTCACAAACGCACGTCAAAGAGTCGGGAGAGGCACAATTCACGAAGTAGAGGAAGAAATAAGGACCGCAGCAGGGACCGCAGCCGCGGCCGGGGCAGCCGGAGCCGGAGCCGGAGCCGCAGGAGCCGCCGCAGCCGGAGCCAAAGTTCCTCTAGGTCCCGAAGTCGTGGCAGGAGGAGGTCGGGTAATAAAGACAGAACTGTTCAGAGTCCCAAATCTAAATAA

>*O. anatinus U2af1-rs2*

atggcggcgcccgcggcgagggacctggcggggaaactgagccacagacagtaccgagccgccctgaagaaggagaagcgcaagaaacggcgacaggaactcgctaggctcagggactcaggctttgcagaaaaagatgaggaaaattccgtggctgaagatgaagaactggaagaagagaaaaaattagaagctgaaaggcaaaaattacatgaggagtggttgctgagggaacaaaaggctcaagaggaattcagactgaagaaggagaaagaggaggcagcaaaaagacgtcaagaagaagaggagagaagaatcaaggaagaatgggaagaacaacaaagaaaagagagagaggaagaggaacagaaactacagaagaagagagacagagaggaagctgtgcagaagatgctggatcaggctgaaagtcagctggaaaacggagtcacgtggcataatccggaaccaccagcagatgtacggataacggagaaagatcgagctaattgtccattctacattaaaacaggatcttgcagatttggagacaggtgttcccgtaagcataactacccaaccctgagcccaacactcctcatcagaagtatgtttataacatttggaatggagcagtgcaggagggatgactatgacactgatgcaagtctggagtacagtgaggaagaaacgtatcaacaattcttagacttctatgaggatgtgcttcctgaattcaagaatgtgggaaaggttattcagttcaaggtcagctgcaactttgagcctcatctgcggggcaatgtgtatgttcaatatcagtcggaagaagagtgtcaggaagccttctctctgttcaatgggaggtggtatgcaggacgacagcttcagtgtgaattctgccccgtcacccgctggaaaatggctatttgtggtttatttgaaaggcaaaagtgccccagggggaagcactgtaactttcttcatgtgttcagaaatcccaacaacgagttctgggaagctaatagggacatccacatgtccccggaccggactagtcaatcctttggcagaagctcggagaggagggaccggccgagccactacgaagactatcacggaaggtcaaggagacgacgcagccccagtcccgattattcttataaaagaaatggggagtcggagagaaagaagcgaagcagccacaggagtagcaagtatcataagagctcctcaagaagcagtgacaagagaagttcgcacagcagagggagaaagagggatcgcagccgcagcagaagccgaaggagccagagcaggagcccatccaggtccaggagtcggggcaggaggaggtcagccagcagagagaggaacccgcagagtcccaaatccaagtaa

>*M. musculus U2af1-rs1*

ATGGCATCACGGCAGACCGCGATTCCTGAGAAACTCAGCCGAAAACAATACAAGGCGGCAATGAAGAAGGAGAAACGCAAGAAACGTCGGCAGAAAATGGCTCGGCTGAGAGCTCTGGAAGCCCCACCAGAGGAGGACGATGATGTTTCTGCTAACGAAGAACTTGCAGAGCGATTACTGGAGATAGAGCGGCAAAGATTACATGAAGAGTGGCTGCTGAGGGAGGAGAAGGCGCAAGAAGAATTCAGAATAAAGAAGAAAAAGGAAGAGGCCGCTAGAAAACAGAAGGAAGAACAGGAGAGACAAATAAAGGCCGAGTGGGAAGAACAACAGAAAAAACAGAGAGAGGAGGAGGAGCAGAAGCTACAGGAGAAGAGAGAGAGGGAGGAAGCGGTGCAGAAAATGCTGGACCAGGCTGAAAATGAGCGCATTTGGCAGAACCCGGAACCACCCAAGGATTTAAGGCTGGAGAAATATCGACCCAGTTGTCCCTTCTACAATAAAACGGGAGCGTGCAGATTTGGTAACAGATGTTCACGGAAACACGACTTTCCCACGTCAAGTCCCACCCTTCTTGTGAAGAGTATGTTTACAACGTTTGGAATGGAGCAGTGCAGAAGGGATGACTATGACTCAGACGCAAACCTGGAGTACAGTGAGGAGGAGACCTACCAGCAGTTCTTGGATTTCTACCATGACGTGCTGCCGGAGTTCAAGAACGTGGGAAAGGTGATTCAGTTCAAAGTAAGCTGCAACCTGGAACCTCATCTGCGGGGCAATGTGTATGTTCAGTACCAGTCGGAAGAAGAATGCCAAGCAGCCCTCTCTCTCTTTAATGGAAGATGGTACGCAGGACGACAGCTCCAGTGTGAATTCTGTCCAGTGACCCGGTGGAAGGTTGCAATTTGTGGTTTATTCGAAATGCAAAAGTGTCCAAAAGGAAAGCACTGCAACTTCCTTCATGTGTTCAGAAATCCCAACAACGAATTTAGAGAAGCTAACAGAGACATCTACATGTCTCCTCCGGCTTGGACTGGCTCCTCTGGTAAAAACTCAGACAGGAGGGAAAGGAAGGACCATCATGAGGAATACTATAGCAAGTCAAGAAGCTACCACTCTGGTTCATACCACTCCTCCAAGAGAAACAGGGAGTCCGAGAGGAAGAGTCCTCACAGGTGGAAGAAATCTCACAAACAGACAACGAAGAGTCATGAGAGGCACAGTTCAAGAAGAGGAAGAGAAGAGGACAGCAGTCCAGGTCCACAAAGCCAGAGCCACAGAACCTGA

>*R. novergicus U2af1-rs1*

ATGGCATCACAGCAGACCGCGTTTCCTGAGAAACTCAGCAGAAAACAATACAAGGCTGCAATGAAGAAGGAGAAACGCAAGAAACGTCGGCAGAAAATGGCTCGCTTGAGAGCTCTGGAAGCCCCACCAGAGGTGGAGGAGGAGGATGTTTCTGCCAATGAAGAACTTGCAGAGAGATTACTGGAGATAGAGAGGCAAAAATTACATGAAGAGTGGCTGCTGAGGGAGGAGAAGGCACAAGAAGAATTCAGAATAAAGAAGAAAAAGGAGGAGGCTGCTAGAAAACAGAAGGAAGAACAGGAGAGACAAATAAAGGCTGAATGGGAAGAACAACAGAAAAAACAGAGAGAGGAGGAGGAGCAGAAGCTACAGGAGAAGAAAGAGAGAGAGGAAGCGGTGCAGAAAATGCTGGACCAGGCTGAAAATGAAGGCACTTGGCAGAACCCAGAACCACCCAAAGATTTAAGGCTGGAGAAATATCGACCCAGTTGTCCCTTCTACAATAAGACGGGAGCCTGCAGATTTGGTAACAGGTGTTCACGGAAGCATGACTTTCCCACGTCAAGTCCCACACTTCTTGTGAAGAGTATGTTTACGACGTTTGGAATGGAGCAGTGCAGAAGGGATGACTACGACTCTGACGCAAACCTGGAGTACAGTGAGGAGGAGACCTACCAGCAGTTTTTGGATTTCTACCACGACGTGCTGCCCGAGTTCAAGAACGTGGGAAAGGTGATTCAGTTCAAAGTAAGCTGCAATCTGGAACCTCATTTGCGGGGCAATGTGTATGTTCAGTACCAGTCGGAAGAGGAATGCCAAGCAGCCCTCTCTCTCTTTAATGGAAGATGGTACGCAGGACGACAGCTGCAGTGTGAATTCTGTCCAGTGACCCGGTGGAAGGTTGCGATTTGCGGTTTGTTCGAAATGCAAAAGTGTCCGAAAGGAAAGCACTGCAACTTCCTCCATGTGTTCAGAAACCCCAACAACGAGTTCAGAGACGCGAACAGGGACATCTACCTGCCTCCGGCGAGCACTGGCTCCTCTGGTAAAAACTCAGACCGGGGGGACAGGAAGGATCACCGAGAGGAAAGCTACAGCAAGTCAAGAAGCCACCACTCTGGCTCATACCACTCCTCCAAGAGAAGCAGGGAGTCGGAGAGGAAGAGTCCTCACAGGTGGAAGAAATCTCACAAACAGGCCACGAAGAGTCATGAGCGGCACAGCTCAAGAAGAGGAAGAGAAGAGGGCAGCAGTCCAGGTCCGCAAAGCCAGAGCCACAGAACCTGA
