# Supplementary material for: A Case-by-Case Evolutionary Analysis of Four Imprinted Retrogenes
Source: Evolution. 2011 May;65(5):1413–27. doi: 10.1111/j.1558-5646.2010.01213.x (PMC3107425; doi:10.1111/j.1558-5646.2010.01213.x)
Supplement: Supplementary file 2 [file evo0065-1413-SD2.doc]

| **Gene Name** | **Forward Primer** | **Reverse Primer** |
| --- | --- | --- |
| *Mcts2* | TGCCATCCTTCCTGTAGACC | CCACAGCCCGTCATTTAGATA |
| *Mcts1* | TGCATCCAGTTGAAAACCTC | GCCAAGTTTGCTACCACATTAT |
| *U2af1-rs1* | CGTGGGAAAGGTGATTCAGT | TGACAAAGCATCACAATGTAAAGT |
| *U2af1-rs2* | CTGAGGGAAGAAAAGGCACA | CAACACAGCCACATCCATTC |
| *Nap1l5* | CCCCTTTTATTTCCAATGTCGG | AATGCTGTCAGTCCAGGCTTGC |
| *Nap1l2* | GCGGCTCTCTGCAGTCTCTA | CGTTGCCGTTAGTCTCTTCC |
| *Nap1l3* | TTTTTCCTGGTAATGTCCTAAAAAG | GCATGGCAGTTTATTTGATATTTACTC |
| *Inpp5f_v2* | CCGCTTGTTTTGTGCCAAATG | CCGAAAAGAGTAGGTTCAGGACC |
| *Vma21* | ACGGTTCCTATCGGCTTGTA | GAGCAATCTTTCCTCACTGACTT |
| *b-Actin* | TGTTACCAACTGGGACGACA | CTCTCAGCTGTGGTGGTGAA |
