# Supplementary material for: A Case-by-Case Evolutionary Analysis of Four Imprinted Retrogenes
Source: Evolution. 2011 May;65(5):1413–27. doi: 10.1111/j.1558-5646.2010.01213.x (PMC3107425; doi:10.1111/j.1558-5646.2010.01213.x)
Supplement: Supplementary file 3 [file evo0065-1413-SD3.doc]

**McCole et al 2010 Additional Table 1. Shimodaira-Hasegawa (SH) test of gene phylogeny versus species phylogeny**

| **Gene Family** | **SH p-value**  **Gene Phylogeny** | **SH p-value**  **Species Phylogeny** | **Best-fit Phylogeny**  **(5 % significance level)** |
| --- | --- | --- | --- |
| **Inpp5f_v2-Vma21** | 1.0000 | 0.1920 | equal |
| **Mcts** | *1.0000* | *0.0290* | *gene* |
| **Nap1l** | 1.0000 | 0.4450 | equal |
| **U2af1-rs** | 1.0000 | 0.1690 | equal |

Note: Tree with the lowest likelihood is given a p-value of 1. For the alternative phylogeny, a p-value below the 5 % significance level (p-value < 0.05), indicates significant exclusion of that phylogeny, resulting in the tree with the lowest likelihood being the best-fit phylogeny to the observed data.
