# Supplementary material for: A Case-by-Case Evolutionary Analysis of Four Imprinted Retrogenes
Source: Evolution. 2011 May;65(5):1413–27. doi: 10.1111/j.1558-5646.2010.01213.x (PMC3107425; doi:10.1111/j.1558-5646.2010.01213.x)
Supplement: Supplementary file 4 [file evo0065-1413-SD4.doc]

**Supplementary File 4. Description of models.** Each gene family either showed M3: K=2 or Model B as the best fit to the data (bold highlight).

| **Model Name** | **Characteristics** | **Positive selection (ω>1)?** |
| --- | --- | --- |
| M0 | One ω allowed across all sites | Allowed |
| **Site-specific models** |  |  |
| M1: neutral | Two classes, ω_1_ fixed at 0 and ω_2_ fixed at 1. Returns the proportion p_2_ of sites in the second category. 1- p_2_ returns p_1_ | Not allowed |
| M2: Selection | M1 plus an additional class where ω is estimated from the data and can be larger than 1. | Allowed |
| **M3: Discrete K=2** | **Two classes of ω allowed without constraint on either value, these values are estimated as are their relative proportions and ω can be larger than 1** | **Allowed** |
| M3: Discrete K=3 | As M3(K=2) but with 3 unconstrained classes of ω | Allowed |
| M7: beta | ω is assumed to have a beta distribution between values of 0 and 1 inclusive. 10 classes of ω allowed but none >1 | Not allowed |
| M8: beta and omega>1 | As Model 7 but a further ω category is estimated from the data and can be larger than 1. | Allowed |
| M8a: beta and omega=1 | As Model 8 but with ω fixed to 1 – is the null of M8. | Not allowed |
| **Lineage-specific models** |  |  |
| Model A | Lineage-specific extension of M1. Four ω classes allowed, two of which can vary between the foreground and background lineages, one which is unconstrained apart from equality for the foreground and background lineages, and one which is set ω=1 | Allowed |
| Model A null | As Model A but with both the ω classes that are allowed to differ between the foreground and background lineages set to 1 | Not allowed |
| **Model B** | **Lineage-specific extension of Model 3(K=2). Four classes or ω allowed, two of which can vary between the foreground and background lineages, no constraints** | **Allowed** |
