# Supplementary material for: A Case-by-Case Evolutionary Analysis of Four Imprinted Retrogenes
Source: Evolution. 2011 May;65(5):1413–27. doi: 10.1111/j.1558-5646.2010.01213.x (PMC3107425; doi:10.1111/j.1558-5646.2010.01213.x)
Supplement: Supplementary file 6 [file evo0065-1413-SD6.pdf]

Inpp5f_v2-Vma21 family codon evolution results

Model	omega used	lnL	P	Estimates of parameters
M0 : one ratio	0	-2039.75	1	$\omega = 0.10$
**Site-specific:**				
M1:Neutral	0	-2039.75	2	p_0= 0.99, ω_0=0.10
M2:Selection	0	-2039.75	4	p_0= 1, p_1= 0<br>ω_0=0.10, ω_1 = 1
**M3:Discrete(K = 2)**	**0**	**-2024.97**	**3**	**p_0= 049, (p_1= 0.51)<br>ω_0= 0.02, ω_1= 0.19**
M3:Discrete(K = 3)	0	-2024.97	5	p_0= 0.49, p_1= 0.51<br>ω_0= 0.02, ω_1=0.19
M7: Beta	0	-2025.79	2	p= 0.95, q= 7.83
M8: Beta&Omega > 1	0	-2025.79	4	p_0= 0.99, p = 0.95, q = 7.83<br>(p_1= 0.00001), ω =1
M8a: Beta&Omega = 1	1	-2025.79	3	p_0= 0.99, p = 0.95, q = 7.83<br>(p_1= ?), ω = 1
**Branch-specific**				
Model A	0	-2029.53	4	p_0 = 0.40, p_1 = 0,<br>(p_2 = 0.60, p_3 = 0), ω_0 = 0.09,<br>ω_1 = 1, ω_2 = 1
Model A null	1	-2029.53	3	p_0 = 0.40, p_1 = 0,<br>(p_2 = 0.60, p_3 = 0), ω_0 = 0.09,<br>ω_1 = 1, ω_2 = 1
**Model B**	**0**	**-2020.32**	**5**	**p_0 = 0.23, p_1 = 0.22, p_2 = 0.28, p_3 = 0.27<br>Foreground:<br>ω_0 = 0.01, ω_1 = 0.19, ω_2 = 0.80, ω_3 = 0.80<br>Background:<br>ω_0 = 0.01, ω_1 = 0.19, ω_2 = 0.01, ω_3 = 0.19**

Comparison	*df*	*Δl*	Critical χ^2 values
**M0 v M3k2**	**2**	**2(2039.75-2024.97)=29.56***	**≥ 5.99**
M3k2 v M3k3	-	1(2024.97-2024.97)=0	≥ 1.00
M1 v M2	2	2(2039.75-2039.75)=0	≥ 5.99
M7 v M8	2	2(2025.79-2025.79)=0	≥ 5.99
M8 v M8a	1	2(2025.79-2025.79)=0	≥ 2.71 (@5%)<br>≥ 5.41 (@1%)
M1 v Model A	2	2(2039.75-2029.53)=20.44*	≥ 5.99
Model A v Model A null	1	2(2029.53-2029.53)=0	≥ 3.84 (@5%)
**M3k2 v Model B**	**2**	**2(2024.97-2020.32)=9.3***	**≥ 5.99**

Mcts family codon evolution results

Model	omega used	lnL	P	Estimates of parameters
M0 : one ratio	0	-2554.31	1	$\omega = 0.04$
**Site-specific**				
M1:Neutral	0	-2539.00	2	p_0= 0.96, ω_0<0.03
M2:Selection	0	-2539.00	4	p_0= 0.96, p_1= 0.04 ω_0=0.03, ω_1 = 1
**M3:Discrete(K = 2)**	**0**	**-2528.13**	**3**	**p_0= 0.89, (p_1= 0.11) ω_0= 0.01, ω_1= 0.27**
M3:Discrete(K = 3)	0	-2527.49	5	p_0= 0.52, p_1= 0.41, (p_2= 0.07) ω_0= 0, ω_1=0.05, ω_2= 0.34
M7: Beta	0	-2528.36	2	p= 0.19, q= 3.65
M8: Beta&Omega > 1	0	-2525.36	4	p_0= 0.10, p = 0.185, q = 3.66 (p_1= 0.00001), ω =1.00
M8a: Beta&Omega = 1	1	-2528.36	3	p_0= 0.99, p = 0.19, q = 3.66 (p_1= 0.00001), ω = 1
**Branch-specific**				Values for Foreground
Model A	0	-2536.79	4	p_0 = 0.003, p_1 = 0.00001, (p_2 = 0.96, p_3 = 0.04), ω_0 = 0.02, ω_1 = 1, ω_2 = 1.00
Model A null	1	-2536.79	3	p_0 = 0.0006, p_1 = 0.00003, (p_2 = 0.96, p_3 = 0.04), ω_0 = 0.02, ω_1 = 1, ω_2 = 1
Model B	0	-2525.16	5	p_0 = 0.00, p_1 = 0.00, (p_2 = 0.88, p_3 = 0.11), ω_0 = 0.1, ω_1 = 0.26, ω_2 = 2.73

Comparison	*df*	*Δl*	Critical χ^2 values
**M0 v M3k2**	**2**	**2(2554.31-2528.13)=52.36***	**≥ 5.99**
M3k2 v M3k3	-	1(2528.13-2527.49)=0.64	≥ 1.00
M1 v M2	2	2(2539.00-2539.00)=0	≥ 5.99
M7 v M8	2	2(2528.36-2525.36)=0	≥ 5.99
M8 v M8a	1	2(2528.36-2525.36)=0	≥ 2.71 (@5%) ≥ 5.41 (@1%)
M1 v Model A	2	2(2539.00-2536.79)=4.42	≥ 5.99
Model A v Model A null	1	2(2536.79-2539.79)=0	≥ 3.84 (@5%)
M3k2 v Model B	2	2(2528.13-2525.16)=5.94	≥ 5.99

Nap1l family codon evolution results

Model	omega used	lnL	P	Estimates of parameters
M0 : one ratio	0	-12832.55	1	ω = 0.19
**Site-specific:**				
M1:Neutral	0	-12733.70	2	p_0= 0.75, , p_1=0.25 ω_0=0.13, ω_1=1
M2:Selection	0	-12733.70	4	p_0= 0.75, p_1= 0.26 ω_0=0.14, ω_1 = 1
**M3:Discrete(K = 2)**	**0**	**-12667.28**	**3**	**p_0= 0.50, p_1= 0.50 ω_0= 0.06, ω_1= 0.38**
M3:Discrete(K = 3)	0	-12667.28	5	p_0= 0.50, p_1= 0.50 ω_0= 0.06, ω_1=0.38
M7: Beta	0	-12663.91	2	p= 0.94, q= 3.27
M8: Beta&Omega > 1	0	-12663.91	4	p_0= 0.99, p = 0.94, q = 3.27 (p_1= 0.01), ω =1
M8a: Beta&Omega = 1	1	-12663.91	3	p_0= 0.99, p = 0.94, q = 3.27 (p_1= 0.01), ω = 1
**Branch-specific**				
Model A	0	-12727.76	4	p_0 = 0.47, p_1 = 0.15, p_2 = 0.38, ω_0 = 0.13, ω_1 = 1, ω_2 = 2.22
Model A null	1	-12728.61	3	p_0 = 0.45, p_1 =0.15 , p_2 = 0.31, ω_0 = 0.13, ω_1 = 1, ω_2 = 1
Model B	0	-12664.50	5	p_0 = 0.33, p_1 = 0.33, p_2 = 0.34, ω_0 = 0.06, ω_1 = 0.37, ω_2 = 1.07

Comparison	*df*	*Δl*	Critical χ^2 values
**M0 v M3k2**	**2**	**2(12832.55-12667.28)=330.54***	**≥ 5.99**
M3k2 v M3k3	-	1(12667.28-12667.28)=0	≥ 1.00
M1 v M2	2	2(12733.70-12633.70)=0	≥ 5.99
M7 v M8	2	2(12663.91-12663.91)=0	≥ 5.99
M8 v M8a	1	2(12663.91-12993.91)=0	≥ 2.71 (@5%) ≥ 5.41 (@1%)
M1 v Model A	2	2(12733.70-12727.76)=11.88*	≥ 5.99
Model A v Model A null	1	2(12727.76-12728.61)=-1.7	≥ 3.84 (@5%)
M3k2 v Model B	2	2(12667.28-12664.50)=5.56	≥ 5.99

U2af1-rs family codon evolution results

Model	omega used	lnL	P	Estimates of parameters
M0 : one ratio	0	-7449.37	1	$\omega = 0.14$
**Site-specific:**	0	-7304.01		
M1:Neutral			2	$p_0 = 0.83$, $\omega_0 < 1$
M2:Selection	0	-7302.37	4	$p_0 = 0.83$, $p_1 = 0.16$ ($p_2 = 0.008$), $\omega_0 < 0.074$, $\omega_1 = 1$, $\omega_2 = 38.93$
M3:Discrete(K = 2)	0	-7302.16	3	$p_0 = 0.80$, ($p_1 = 0.20$) $\omega_0 = 0.06$, $\omega_1 = 0.78$
**M3:Discrete(K = 3)**	**2**	**-7293.06**	**5**	**$p_0 = 0.58$, $p_1 = 0.31$, ($p_2 = 0.10$) $\omega_0 = 0.033$, $\omega_1 = 0.24$, $\omega_2 = 1.30$**
M7: Beta	0	-7307.48	2	p= 0.32, q= 1.32
M8: Beta&Omega > 1	0	-7293.27	4	$p_0 = 0.90$, p = 0.68, q = 5.46 ($p_1 = 0.100$), $\omega = 1.31$
M8a: Beta&Omega = 1	1	-7294.55	3	$p_0 = 0.87$, p = 0.80, q = 7.49 ($p_1 = 0.13$), $\omega = 1$
**Branch-specific**				
Model A	0	-7286.39	4	$p_0 = 0.77$, $p_1 = 0.16$, ($p_2 = 0.06$, $p_3 = 0.01$), $\omega_0 = 0.06$, $\omega_1 = 1$, $\omega_2 = 3.91$, $\omega_3 = 3.91$
Model A null	1	-7289.73	3	$p_0 = 0.72$, $p_1 = 0.14$, ($p_2 = 0.11$, $p_3 = 0.02$), $\omega_0 = 0.06$, $\omega_1 = 1$, $\omega_2 = 1$
**Model B**	**0**	**-7284.63**	**5**	**$p_0 = 0.74$, $p_1 = 0.18$, $p_2 = 0.06$, $p_3 = 0.01$ Foreground: $\omega_0 = 0.06$, $\omega_1 = 0.79$, $\omega_2 = 3.74$, $\omega_3 = 3.74$ Background: $\omega_0 = 0.06$, $\omega_1 = 0.79$, $\omega_2 = 0.06$, $\omega_3 = 0.78$**

Comparison	*df*	*Δl*	Critical χ^2 values
M0 v M3k2	2	2(7449.37-7302.16)=294.42*	≥ 5.99
M3k2 v M3k3	-	1(7302.16-7293.06)=9.1*	≥ 1.00
M1 v M2	2	2(7304.01-7302.37)=3.28	≥ 5.99
M7 v M8	2	2(7307.48-7293.27)=29.06*	≥ 5.99
M8 v M8a	1	2(7293.27-7294.35)=2.56	≥ 2.71 (@5%) ≥ 5.41 (@1%)
M1 v Model A	2	2(7304.01-7286.39)=35.42*	≥ 5.99
Model A v Model A null	1	2(7289.73-7286.39)=6.68*	≥ 3.84 (@5%)
**M3k2 v Model B**	**2**	**2(7302.16-7284.63)=107.06***	**≥ 5.99**
