# Supplementary material for: A Case-by-Case Evolutionary Analysis of Four Imprinted Retrogenes
Source: Evolution. 2011 May;65(5):1413–27. doi: 10.1111/j.1558-5646.2010.01213.x (PMC3107425; doi:10.1111/j.1558-5646.2010.01213.x)
Supplement: Supplementary file 7 [file evo0065-1413-SD7.doc]

**(a) Inpp5f_v2-Vma21 Species Phylogeny codon evolution**

| **Model** | **P** | **Initial/Fixed***  **Omega** | **Likelihood** | **Estimates of parameters** | **Positively**  **selected sites** |
| --- | --- | --- | --- | --- | --- |
| M0 : one ratio | 1 | 0 | -2051.07 | ω = 0.10 | none |
| **Site-specific:**  M1:Neutral | 2 | 0 | -2051.07 | p_0_= 1.00, ω_0_ = 0.10 | not allowed |
| M2:Selection | 4 | 0 | -2051.07 | p_0_= 1.00, p_1_= 0.00  (p_2_= 0.00), ω_0_ = 0.10,  ω_1_ = 1.00, ω_2_ = 64.04 | none |
| **M3:Discrete(K = 2)** | **3** | **0** | **-2036.55** | **p_0_= 0.46, (p_1_= 0.54)**  **ω_0_= 0.02, ω_1_= 0.18** | **none** |
| M3:Discrete(K = 3) | 5 | 0 | -2036.55 | p_0_= 0.46, p_1_= 0.54,  (p_2_= 0.00)  ω_0_= 0.02, ω_1_= 0.18,  ω_2_ = 12.69 | none |
| M7: Beta | 2 | 0 | -2037.64 | p= 0.97, q= 7.89 | not allowed |
| M8: Beta&Omega > 1 | 4 | 0 | -2037.64 | p_0_= 1.00, p = 0.97,  q = 7.89  (p_1_= 0.00), ω = 3.39 | none |
| M8a: Beta&Omega = 1 | 3 | 1* | -2037.64 | p_0_=1.00, p = 0.97,  q = 7.89  (p_1_= 0.00), ω = 1.00 | Not allowed |
| **Branch-specific** |  |  |  |  |  |
| Model A | 4 | 0 | -2040.55 | p_0_ = 0.40, p_1_ = 0.00,  (p_2_ = 0.60, p_3_ = 0.00), ω_0_ = 0.09, ω_1_ = 1.00, ω_2_ = 1.03 | BEB  100 > 50 %  9 > 95 %  6 > 99 % |
| Model A null | 3 | 1* | -2040.55 | p_0_ = 0.40, p_1_ = 0.00,  (p_2_ = 0.60, p_3_ = 0.00),  ω_0_ = 0.09, ω_1_ = 1.00, ω_2_ = 1.00 | not allowed |
| **Model B** | **5** | **0** | **-2031.10** | **p_0_ = 0.26, p_1_ = 0.19,**  **(p_2_ = 0.31, p_3_ = 0.25),**  **ω_0_ = 0.17, ω_1_ = 0.00, ω_2_ = 0.86** | **none** |

**(b) NAP1l Species Phylogeny codon evolution.**

| **Model** | **P** | **Initial/Fixed***  **Omega** | **Likelihood** | **Estimates of parameters** | **Positively**  **selected sites** |
| --- | --- | --- | --- | --- | --- |
| M0 : one ratio | 1 | 0 | -12826.39 | ω = 0.19 | none |
| **Site-specific:**  M1:Neutral | 2 | 0 | -12726.47 | p_0_= 0.75, ω_0_ = 0.14 | not allowed |
| M2:Selection | 4 | 0 | -12726.47 | p_0_= 0.75, p_1_= 0.16  (p_2_ = 0.09), ω_0_ = 0.14,  ω_1_ = 1.00 , ω_2_ = 1.00 | none |
| **M3:Discrete(K = 2)** | **3** | **0** | **-12660.57** | **p_0_ = 0.50, (p_1_= 0.49)**  **ω_0_ = 0.06, ω_1_= 0.38** | **none** |
| M3:Discrete(K = 3) | 5 | 1 | -12655.26 | p_0_ = 0.36, p_1_= 0.37,  (p_2_ = 0.26)  ω_0_ = 0.05, ω_1_= 0.20,  ω_2_ = 0.52 | none |
| M7: Beta | 2 | 0 | -12656.82 | p = 0.95, q = 3.26 | not allowed |
| M8: Beta&Omega > 1 | 4 | 0 | -12656.82 | p_0_= 1.00, p = 0.95,  q = 3.26  (p_1_= 0.00 ), ω = 1.00 | none |
| M8a: Beta&Omega = 1 | 3 | 1* | -12656.82 | p_0_= 0.99, p = 0.95,  q = 3.26  (p_1_= 0.00), ω = 1.00 | not allowed |
| **Branch-specific** |  |  |  |  |  |
| Model A | 4 | 0 | -12720.39 | p_0_ = 0.44, p_1_ = 0.14,  (p_2_ = 0.31, p_3_ = 0.10),  ω_0_ = 0.13, ω_1_ = 1.00, ω_2_ = 2.05 | BEB  46 > 50 % |
| Model A null | 3 | 1* | -12721.14 | p_0_ = 0.43, p_1_ = 0.14,  (p_2_ = 0.32, p_3_ = 0.11),  ω_0_ = 0.13, ω_1_ = 1.00, ω_2_ = 1.00 | not allowed |
| Model B | 5 | 0 | -12658.01 | p_0_ = 0.33, p_1_ = 0.33,  (p_2_ = 0.17, p_3_ = 0.17),  ω_0_ = 0.06, ω_1_ = 0.38, ω_2_ = 1.03 | none |

**(c) U2af1-rs Species Phylogeny codon evolution**

| **Model** | **P** | **Initial/Fixed***  **Omega** | **Likelihood** | **Estimates of parameters** | **Positively**  **selected sites** |
| --- | --- | --- | --- | --- | --- |
| M0 : one ratio | 1 | 0 | -7455.10 | ω = 0.14 | none |
| **Site-specific:**  M1:Neutral | 2 | 0 | -7304.93 | p_0_ = 0.83, ω_0_ = 0.07 | not allowed |
| M2:Selection | 4 | 0 | -7303.32 | p_0_= 0.83, p_1_= 0.17  (p_2_ = 0.01), ω_0_ = 0.07,  ω_1_ = 1.00, ω_2_ = 39.08 | none |
| M3:Discrete(K = 2) | 3 | 0 | -7303.29 | p_0_ = 0.80, (p_1_= 0.20)  ω_0_ = 0.06, ω_1_= 0.80 | none |
| **M3:Discrete(K = 3)** | **5** | **0** | **-7295.05** | **p_0_ = 0.58, p_1_= 0.31,**  **(p_2_ = 0.11)**  **ω_0_ = 0.03, ω_1_= 0.23,**  **ω_2_ = 1.25** | **NEB**  **39 > 50 %**  **16 > 95 %**  **5 > 95 %** |
| M7: Beta | 2 | 0 | -7309.30 | p = 0.32, q = 1.29 | not allowed |
| M8: Beta&Omega > 1 | 4 | 0 | -7295.18 | p_0_= 0.89, p = 0.69,  q = 5.74  (p_1_= 0.11), ω = 1.26 | BEB  13 > 50 % |
| M8a: Beta&Omega = 1 | 3 | 1* | -7296.15 | p_0_= 0.87, p = 0.81,  q = 7.76  (p_1_=0.13 ), ω = 1.00 | not allowed |
| **Branch-specific** |  |  |  |  |  |
| **Model A** | **4** | **0** | **-7287.36** | **p_0_ = 0.77, p_1_ = 0.16,**  **(p_2_ = 0.06, p_3_ = 0.01,**  **ω_0_ = 0.07, ω_1_ = 1.00, ω_2_ = 4.16** | **BEB**  **13 > 50 %**  **5 > 95 %**  **3 > 99%** |
| Model A null | 3 | 1* | -7290.75 | p_0_ = 0.72, p_1_ = 0.15,  (p_2_ = 0.11, p_3_ = 0.02),  ω_0_ = 0.06, ω_1_ = 1.00, ω_2_ = 1.00 | not allowed |
| Model B | 5 | 0 | -7285.73 | p_0_ = 0.74, p_1_ = 0.18,  (p_2_ = 0.06, p_3_ = 0.01),  ω_0_ = 0.06, ω_1_ = 0.80, ω_2_ = 3.86 | NEB  14 > 50%  4 > 95%  3 > 99 % |
