# Supplementary material for: A Case-by-Case Evolutionary Analysis of Four Imprinted Retrogenes
Source: Evolution. 2011 May;65(5):1413–27. doi: 10.1111/j.1558-5646.2010.01213.x (PMC3107425; doi:10.1111/j.1558-5646.2010.01213.x)
Supplement: Supplementary file 8 [file evo0065-1413-SD8.pdf]

```
>HumanIn
MPRSHRPPPTAQRAAEPKSEGVVAMTFKIFLLFAGLMVKVPVGLYFSCKLLLFQSLMLMSPEDSGFYATIVAVVGLHVVLAIFVFIVWKEGLP
>MouseIn
MPRSHRQPPPAPRAPEPKSDGVLAMTFKIFLLFAGLMVKVPVGLYFSCKLLLFQSLMLMSPEDSAFYATIVSVVGLHVVLAIFVFIVWKEGLP
>RatIn
MPCSHRQPPPVPRAPEPKSDGVLAMTFKIFLLFAGLMVKVPVGLYFSCKLLLFQSLMLMSPEDSAFYATIVSVVGLHVVLATFVFIVWEEGLP
>DogIn
MPRSHRPPPTAQRPAEPKPEGSLATTFKIFLLFAGLMVKVPVGLYFSCKLLLFQSLLLMSPDDSGFYATIVAVVGLHVVLAVFVFIVWKEGMP
>CowIn
MPRSHRPPPTAQRQAEPKPEGSLATTFKIFLLFAGLMVKVPVGLYFSCKLLLFQSLLLMSPDDSGFYATIVAVVGLHVVLAVFVFIVWKEGMP
>ElephIn
MPRSHRHPPAAPRTEEPKSDGVLPMTFKIFLLFAGLMVKVPVGLYFSCKLLLFQSLMLMSPEDSAFYATIVAVVGLHVELAFFFIIVWKEGLP
>ArmIn
MPRSHRPPPVAQRPAEPKSDGVLAMTFKIFLLFAGLMVKVPVGLYFSCKLLLFQSLMLMSPEDSAFYATIVAVVGLHVVLAIFVFIVWKEGLL
>HumanTmem
MERPDKAALNALQPPEFRNESSLASTLKTLLFFTALMITVPIGLYFTTKSYIFEGALGMSNRDSYFYAAIVAVVAVHVVLALFVYVAWNEGSR
>MouseTmem
MERLDKAALNALQPPEFRNENSLAATLKTLLFFTALMITVPIGLYFTTKAYIFEGALGMSNRDSYFYAAIVAVVAVHVVLALFVYVAWNEGSR
>CowTmem
MERLDKAALNALQPSDFRNESSLASTLKTLLFFTALMITVPIGLYFTTKSYVFEGAFGMSNRDSYFYAAIVAVVAVHVVLALFVYVAWNEGSR
>DogTmem
MERLDKAALNALQPPDFRNESSLASTLKTLLFFTALMITVPIGLYFTTKSYVFEGAFGMSNRDSYFYAAIVAVVAAHVVLALFVYVAWNEGSR
>ElephTmem
MERLDKAALNALQPPDFRSESSLASTLKTLLFFTALMITVPIGLYFTTKSYVFEGALGMSSRDSYFYAAIVAVVAVHVVLALFLYVAWNEGSR
>ArmTmem
MERFDKAALNALQPPEFRTESSLASTLKTLLFFTALMITVPIGLYFTTKSYLFEGALGMSNRDSYFYAAIVAVVAVHVVLALFVYVAWNEGSR
>OpMTmem
MERLEKTTQSALQPPEQRNEGSLTSTLKTLLVFTALMITLPIGLYFSSKSYVFEGFLGMSNGDSYFYAAIVAVIAVHVVLALFVYVAWNEGSR
>OpTTmem
MERLEKTTLSVLQPPELRNEGSLTSTLKTLLVFTALMITLPIGLYFSSKSYVFEGTLGLSNRDSYFYAAIVAVVAVHVVLALFVYVAWNEGSR
```

```
          *   .: .  . :  : : :. :. *:* :*.*:.**:.:*:****: *  :*:. : :*  ** ***:**:*:. ** ** *. :.*:**  .*:* *.:*
  HumanIn MPRSHRPPPTAQRAAEPKSEGVVAMTFKIFLLFAGLMVKVPVGLYFSCKLLLFQSLMLMSPEDSGFYATIVAVVGLHVVLAIFVFIVWKEGLPQWRENKNEX   102
  MouseIn MPRSHRQPPPAPRAPEPKSDGVLAMTFKIFLLFAGLMVKVPVGLYFSCKLLLFQSLMLMSPEDSAFYATIVSVVGLHVVLAIFVFIVWKEGLPQWRENKNDX   102
    RatIn MPCSHRQPPPVPRAPEPKSDGVLAMTFKIFLLFAGLMVKVPVGLYFSCKLLLFQSLMLMSPEDSAFYATIVSVVGLHVVLATFVFIVWEEGLPQWRENKNDX   102
    DogIn MPRSHRPPPTAQRPAEPKPEGSLATTFKIFLLFAGLMVKVPVGLYFSCKLLLFQSLLLMSPDDSGFYATIVAVVGLHVVLAVFVFIVWKEGMPDWQENKSEX   102
    CowIn MPRSHRPPPTAQRQAEPKPEGSLATTFKIFLLFAGLMVKVPVGLYFSCKLLLFQSLLLMSPDDSGFYATIVAVVGLHVVLAVFVFIVWKEGMPDWREEKNEX   102
  ElephIn MPRSHRHPPAAPRTEEPKSDGVLPMTFKIFLLFAGLMVKVPVGLYFSCKLLLFQSLMLMSPEDSAFYATIVAVVGLHVELAFFFIIVWKEGLPHWRENKNEX   102
    ArmIn MPRSHRPPPVAQRPAEPKSDGVLAMTFKIFLLFAGLMVKVPVGLYFSCKLLLFQSLMLMSPEDSAFYATIVAVVGLHVVLAIFVFIVWKEGLLQWRENKNDX   102
HumanTmem MERPDKAALNALQPPEFRNESSLASTLKTLLFFTALMITVPIGLYFTTKSYIFEGALGMSNRDSYFYAAIVAVVAVHVVLALFVYVAWNEGSRQWREGKQDX   102
MouseTmem MERLDKAALNALQPPEFRNENSLAATLKTLLFFTALMITVPIGLYFTTKAYIFEGALGMSNRDSYFYAAIVAVVAVHVVLALFVYVAWNEGSRQWREGKQDX   102
  CowTmem MERLDKAALNALQPSDFRNESSLASTLKTLLFFTALMITVPIGLYFTTKSYVFEGAFGMSNRDSYFYAAIVAVVAVHVVLALFVYVAWNEGSRQWREGKQDX   102
  DogTmem MERLDKAALNALQPPDFRNESSLASTLKTLLFFTALMITVPIGLYFTTKSYVFEGAFGMSNRDSYFYAAIVAVVAAHVVLALFVYVAWNEGSRQWREGKQDX   102
ElephTmem MERLDKAALNALQPPDFRSESSLASTLKTLLFFTALMITVPIGLYFTTKSYVFEGALGMSSRDSYFYAAIVAVVAVHVVLALFLYVAWNEGSRQWREGKQDX   102
  ArmTmem MERFDKAALNALQPPEFRTESSLASTLKTLLFFTALMITVPIGLYFTTKSYLFEGALGMSNRDSYFYAAIVAVVAVHVVLALFVYVAWNEGSRQWREGKQDX   102
  OpMTmem MERLEKTTQSALQPPEQRNEGSLTSTLKTLLVFTALMITLPIGLYFSSKSYVFEGFLGMSNGDSYFYAAIVAVIAVHVVLALFVYVAWNEGSRQWREGKQDX   102
  OpTTmem MERLEKTTLSVLQPPELRNEGSLTSTLKTLLVFTALMITLPIGLYFSSKSYVFEGTLGLSNRDSYFYAAIVAVVAVHVVLALFVYVAWNEGSRQWREGKQDX   102
    ruler 1.......10........20........30........40........50........60........70........80........90.......100..
```

```
>ElephantMcts1
MGKGRFDEKENVSNCIQLKTSVIKGIKNQLVEQFPGIEPWLNQIMPKKDPVKIVRCHEHI
EILTVNGELLFFRQREGPFYPTLRLLHKYPFILPHQQVDKGAIKFVLSGANIMCPGLTSP
GAKLYPAAVDTIVAIMAEGKQHALCVGVMKMSAEDIEKVNKGIGIENIHYLNDGLWHMKT
YKX
>GorillaMcts1
--KGRFDEKENVSNCIQLKTSVIKGIKNQLIEQFPGIEPWLNQIMPKKDPVKIVRCHEHI
EILTVNGELLFFRQREGPFYPTLRLLHKYPFILPHQQVDKGAIKFVLSGANIMCPGLTSP
GAKLYPAAVDTIVAIMAEGKQHALCVGVMKMSAEDIEKVNKGIGIENIHYLNDGLWHMKT
YKX
>HedgehogMcts1
MGKGRFDEKENVSNCIQLKTSVIKGIKNQLIEQFPGIEPWLNQIMPKKDPVKIVRCHEHI
EILTVNGELLFFRQREGPFYPTLRLLHKYPFILPHQQVDKGAIKFVLSGANIMCPGLTSP
GAKLYPAAVDTVVAIMAEGKQHALCVGVMKMSAEDIEKVNKGIGIENIHYLNDGLWHMKT
YKX
>PlatypusMcts1
-SFDRFDEKENVSNCIQLKTSVIKGIKNQLIDQFPGIEPWLNQIMPKKDPVKIVRCHEHI
EILTVNGELLFFRQREGTFYPTLRLLHKYPFILPHQQVDKGAIKFVLSGANIMCPGLTSP
GAKLYPAASDTVVAIMAEGKQHALCVGVMKMSADDIEKVNKGIGIENIHYLNDGLWHMKT
YKX
>ChickenMcts1
-MFKKFDEKENVSNCIQLKTSVIKGIKNQLIDQFPVIEPWLNQIMPKKDPVKIVRCHEHI
EILTVNGELLFFRQREGIFYPTLRLLHKYPFILPHQQVDKGAIKFVLSGANIMCPGLTSP
GAKLYPAAVDTVVAIMAEGKQHALCVGVMKMSADDIEKVNKGIGIENIHYLNDGLWHMKT
YKX
>ChimpMcts1
MGKGRFDEKENVSNCIQLKTSVIKGIKNQLIEQFPGIEPWLNQIMPKKDPVKIVRCHEHI
EILTVNGELLFFRQREGPFYPTLRLLHKYPFILPHQQVDKGAIKFVLSGANIMCPGLTSP
GAKLYPAAVDTIVAIMAEGKQHALCVGVMKMSAEDIEKVNKGIGIENIHYLNDGLWHMKT
YKX
>CowMcts1
-MFKKFDEKENVSNCIQLKTSVIKGIKNQLIEQFPGIEPWLNQIMPKKDPVKIVRCHEHI
EILTVNGELLFFRQREGPFYPTLRLLHKYPFILPHQQVDKGAIKFVLSGANIMCPGLTSP
GAKLYPAAVDTIVAIMAEGKQHALCVGVMKMSAEDIEKVNKGIGIENIHYLNDGLWHMKT
YKX
>DogMcts1
MGKGRFDEKENVSNCIQLKTSVIKGIKNQLIEQFPGIEPWLNQIMPKKDPVKIVRCHEHI
EILTVNGELLFFRQREGPFYPTLRLLHKYPFILPHQQVDKGAIKFVLSGANIMCPGLTSP
GAKLYPAAVDTIVAIMAEGKQHALCVGVMKMSAEDIEKVNKGIGIENIHYLNDGLWHMKT
YKX
>GuineaMcts1
MGKGRFDEKENVSNCIQLKTSVIKGIKNQLIEQFPGIEPWLNQIMPKKDPVKIVRCHEHI
EILTVNGELLFFRQREGPFYPTLRLLHKYPFILPHQQVDKGAIKFVLSGANIMCPGLTSP
GAKLYPAAADTIVAIMAEGKQHALCVGVMKMSAEDIEKVNKGIGIENIHYLNDGLWHMKT
YKX
>HumanMcts1
-MFKKFDEKENVSNCIQLKTSVIKGIKNQLIEQFPGIEPWLNQIMPKKDPVKIVRCHEHI
EILTVNGELLFFRQREGPFYPTLRLLHKYPFILPHQQVDKGAIKFVLSGANIMCPGLTSP
GAKLYPAAVDTIVAIMAEGKQHALCVGVMKMSAEDIEKVNKGIGIENIHYLNDGLWHMKT
YKX
>MarmosetMcts1mRNA
-MFKKFDEKENVSNCIQLKTSVIKGIKNQLIEQFPGIEPWLNQIMPKKDPVKIVRCHEHI
EILTVNGELLFFRQREGPFYPTLRLLHKYPFILPHQQVDKGAIKFVLSGANIMCPGLTSP
GAKLYPAAVDTIVAIMAEGKQHALCVGVMKMSAEDIEKVNKGIGIENIHYLNDGLWHMKT
YKX
>MouseMcts1
-MFKKFDEKENVSNCIQLKTSVIKGIKNQLLEQFPGIEPWLNQIMPKKDPVKIVRCHEHI
EILTVNGELLFFRQREGPFYPTLRLLHKYPFILPHQQVDKGAIKFVLSGANIMCPGLTSP
GAKLYPAAVDTIVAIMAEGKQHALCVGVMKMSAEDIEKVNKGIGIENIHYLNDGLWHMKT
YKX
>OpossumMcts1
```

```
-MFKKFDEKESVINCIQLKNSVIKGIKNQLTSLFPEIKPWLNQIIPKKDLIKIVRCHEHI
EILTVNGELLFFRQREGPFLPTLRLLHKYPFILPHQQVDKGAIKYVLSGANIMCPGLTSP
GAKLSPAEADTIVAVMAEGKQHALSVGIMKMSSEEIKKVNKGIGIENVHYLNDGLWHMKT
YKX
>RatMcts1
MGKGRFDEKENVSNCIQLKTSVIKGIKNQLLEQFPGIEPWLNQIMPKKDPVKIVRCHEHI
EILTVNGELLFFRQREGPFYPTLRLLHKYPFILPHQQVDKGAIKFVLSGANIMCPGLTSP
GAKLYPAAVDTIVAIMAEGKQHALCVGVMKMSAEDIEKVNKGIGIENIHYLNDGLWHMKT
YKX
>RhesusMcts1mRNA
MGKGRFDEKENVSNCIQLKTSVIKGIKNQLIEQFPGIEPWLNQIMPKKDPVKIVRCHEHI
EILTVNGELLFFRQREGPFYPTLRLLHKYPFILPHQQVDKGAIKFVLSGANIMCPGLTSP
GAKLYPAAVDTIVAIMAEGKQHALCVGVMKMSAEDIEKVNKGIGIENIHYLNDGLWHMKT
YKX
>ChimpMcts2
-MFKKFDEKESVSNCIQLKTSVIKGIKSQLVEQFPGIEPWLNQIMPKKDPVKIVRCHEHT
EILTVSGELLFFRQRKGPFCPTLRLLHKYPFILPHQQVDKGAIKFVLSGANIMCPGLTSP
GAKLYPAAVDTIVAVTAEGKQHALCVGVMKMSAEDIEKVNKGIGIENIHYLNDGLWHMKT
YKX
>MarmosetMcts2
-MFKKFDEKESVSNCIQLKTSVIKGIKSQLIEQFPGIEPWLNQIMPKKDPVKIVRCHEHT
EILTVSGELLFFRQRKGPFCPTLRLLHKYPFILPHQQVDKGAIKFVLSGANIMCPGLTSP
GAKLFPAAVDTIVAVTAEGKQHALCVGVMKMSAEDIEKVNKGIGIENIHYLNDGLWHMKT
YKX
>MouseMcts2
-MFKKFDEKESVSNCIQLKTSVIKGIKSQLTEQFPGIEPWLNQIMPKKDPVKIVRCHEHM
EILTVNGELLFFRQRKGPFYPTLRLLHKYPFILPHQQVDKGAIKFVLSGANIMCPGLTSP
GAKLYTAAVDTIVAVMAEGKEHALCVGVMKMAAADIEKINKGIGIENIHYLNDGLWHMKT
YKX
>OrangutanMcts2
-MFKKFDEKESVSNCIQLKTSVIKGIKSQLVEQFPGIEPWLNQIMPKKDPVKIVRCHEHT
EILTVSGELLFFRQRKGPFCPTLRLLHKYPFILPHQQVDKGAIKFVLSGANIMCPGLTSP
GAKLYPAAVDTIVAVTAEGKQHALCVGVMKMSAEDIEKVNKGIGIENIHYLNDGLWHMKT
YKX
>RatMcts2
-MFKKFDEKESVSNCIQLKTSVIKGIKSQLTEQFPGIEPWLNQIMPKKDPVKIVRCHEHM
EILTVNGELLFFRQRKGPFYPTLRLLHKYPFILPHQQVDKGAIKFVLSGANIMCPGLTSP
GAKLYTAAVDTIVAVMAEGKEHALCVGVMKMAAADIEKINKGIGIENIHYLNDGLWHMKT
YKX
>newhumanMcts2
-MFKKFDEKESVSNCIQLKTSVIKGIKSQLVEQFPGIEPWLNQIMPKKDPVKIVRCHEHT
EILTVSGELLFFRQRKGPFCPTLRLLHKYPFILPHQQVDKGAIKFVLSGANIMCPGLTSP
GAKLYPAAVDTIVAVTAEGKQHALCVGVMKMSAEDIEKVNKGIGIENIHYLNDGLWHMKT
YKX
```

```
             :*****.* ******.*******.** . ** *:******:**** :******** *****.*********:* * ***********************:****************** .*  **:**: ****:***.**:***:: :*:*:********:**************
    EleMcts1 MGKGRFDEKENVSNCIQLKTSVIKGIKNQLVEQFPGIEPWLNQIMPKKDPVKIVRCHEHIEILTVNGELLFFRQREGPFYPTLRLLHKYPFILPHQQVDKGAIKFVLSGANIMCPGLTSPGAKLYPAAVDTIVAIMAEGKQHALCVGVMKMSAEDIEKVNKGIGIENIHYLNDGLWHMKTYKX   183
    GorMcts1 --KGRFDEKENVSNCIQLKTSVIKGIKNQLIEQFPGIEPWLNQIMPKKDPVKIVRCHEHIEILTVNGELLFFRQREGPFYPTLRLLHKYPFILPHQQVDKGAIKFVLSGANIMCPGLTSPGAKLYPAAVDTIVAIMAEGKQHALCVGVMKMSAEDIEKVNKGIGIENIHYLNDGLWHMKTYKX   181
    HedMcts1 MGKGRFDEKENVSNCIQLKTSVIKGIKNQLIEQFPGIEPWLNQIMPKKDPVKIVRCHEHIEILTVNGELLFFRQREGPFYPTLRLLHKYPFILPHQQVDKGAIKFVLSGANIMCPGLTSPGAKLYPAAVDTVVAIMAEGKQHALCVGVMKMSAEDIEKVNKGIGIENIHYLNDGLWHMKTYKX   183
   PlatMcts1 -SFDRFDEKENVSNCIQLKTSVIKGIKNQLIDQFPGIEPWLNQIMPKKDPVKIVRCHEHIEILTVNGELLFFRQREGTFYPTLRLLHKYPFILPHQQVDKGAIKFVLSGANIMCPGLTSPGAKLYPAASDTVVAIMAEGKQHALCVGVMKMSADDIEKVNKGIGIENIHYLNDGLWHMKTYKX   182
  ChickMcts1 -MFKKFDEKENVSNCIQLKTSVIKGIKNQLIDQFPVIEPWLNQIMPKKDPVKIVRCHEHIEILTVNGELLFFRQREGIFYPTLRLLHKYPFILPHQQVDKGAIKFVLSGANIMCPGLTSPGAKLYPAAVDTVVAIMAEGKQHALCVGVMKMSADDIEKVNKGIGIENIHYLNDGLWHMKTYKX   182
  ChimpMcts1 MGKGRFDEKENVSNCIQLKTSVIKGIKNQLIEQFPGIEPWLNQIMPKKDPVKIVRCHEHIEILTVNGELLFFRQREGPFYPTLRLLHKYPFILPHQQVDKGAIKFVLSGANIMCPGLTSPGAKLYPAAVDTIVAIMAEGKQHALCVGVMKMSAEDIEKVNKGIGIENIHYLNDGLWHMKTYKX   183
    CowMcts1 -MFKKFDEKENVSNCIQLKTSVIKGIKNQLIEQFPGIEPWLNQIMPKKDPVKIVRCHEHIEILTVNGELLFFRQREGPFYPTLRLLHKYPFILPHQQVDKGAIKFVLSGANIMCPGLTSPGAKLYPAAVDTIVAIMAEGKQHALCVGVMKMSAEDIEKVNKGIGIENIHYLNDGLWHMKTYKX   182
    DogMcts1 MGKGRFDEKENVSNCIQLKTSVIKGIKNQLIEQFPGIEPWLNQIMPKKDPVKIVRCHEHIEILTVNGELLFFRQREGPFYPTLRLLHKYPFILPHQQVDKGAIKFVLSGANIMCPGLTSPGAKLYPAAVDTIVAIMAEGKQHALCVGVMKMSAEDIEKVNKGIGIENIHYLNDGLWHMKTYKX   183
   GuinMcts1 MGKGRFDEKENVSNCIQLKTSVIKGIKNQLIEQFPGIEPWLNQIMPKKDPVKIVRCHEHIEILTVNGELLFFRQREGPFYPTLRLLHKYPFILPHQQVDKGAIKFVLSGANIMCPGLTSPGAKLYPAAADTIVAIMAEGKQHALCVGVMKMSAEDIEKVNKGIGIENIHYLNDGLWHMKTYKX   183
     HuMcts1 -MFKKFDEKENVSNCIQLKTSVIKGIKNQLIEQFPGIEPWLNQIMPKKDPVKIVRCHEHIEILTVNGELLFFRQREGPFYPTLRLLHKYPFILPHQQVDKGAIKFVLSGANIMCPGLTSPGAKLYPAAVDTIVAIMAEGKQHALCVGVMKMSAEDIEKVNKGIGIENIHYLNDGLWHMKTYKX   182
    MarMcts1 -MFKKFDEKENVSNCIQLKTSVIKGIKNQLIEQFPGIEPWLNQIMPKKDPVKIVRCHEHIEILTVNGELLFFRQREGPFYPTLRLLHKYPFILPHQQVDKGAIKFVLSGANIMCPGLTSPGAKLYPAAVDTIVAIMAEGKQHALCVGVMKMSAEDIEKVNKGIGIENIHYLNDGLWHMKTYKX   182
     MoMcts1 -MFKKFDEKENVSNCIQLKTSVIKGIKNQLLEQFPGIEPWLNQIMPKKDPVKIVRCHEHIEILTVNGELLFFRQREGPFYPTLRLLHKYPFILPHQQVDKGAIKFVLSGANIMCPGLTSPGAKLYPAAVDTIVAIMAEGKQHALCVGVMKMSAEDIEKVNKGIGIENIHYLNDGLWHMKTYKX   182
     OpMcts1 -MFKKFDEKESVINCIQLKNSVIKGIKNQLTSLFPEIKPWLNQIIPKKDLIKIVRCHEHIEILTVNGELLFFRQREGPFLPTLRLLHKYPFILPHQQVDKGAIKYVLSGANIMCPGLTSPGAKLSPAEADTIVAVMAEGKQHALSVGIMKMSSEEIKKVNKGIGIENVHYLNDGLWHMKTYKX   182
    RatMcts1 MGKGRFDEKENVSNCIQLKTSVIKGIKNQLLEQFPGIEPWLNQIMPKKDPVKIVRCHEHIEILTVNGELLFFRQREGPFYPTLRLLHKYPFILPHQQVDKGAIKFVLSGANIMCPGLTSPGAKLYPAAVDTIVAIMAEGKQHALCVGVMKMSAEDIEKVNKGIGIENIHYLNDGLWHMKTYKX   183
    RheMcts1 MGKGRFDEKENVSNCIQLKTSVIKGIKNQLIEQFPGIEPWLNQIMPKKDPVKIVRCHEHIEILTVNGELLFFRQREGPFYPTLRLLHKYPFILPHQQVDKGAIKFVLSGANIMCPGLTSPGAKLYPAAVDTIVAIMAEGKQHALCVGVMKMSAEDIEKVNKGIGIENIHYLNDGLWHMKTYKX   183
  ChimpMcts2 -MFKKFDEKESVSNCIQLKTSVIKGIKSQLVEQFPGIEPWLNQIMPKKDPVKIVRCHEHTEILTVSGELLFFRQRKGPFCPTLRLLHKYPFILPHQQVDKGAIKFVLSGANIMCPGLTSPGAKLYPAAVDTIVAVTAEGKQHALCVGVMKMSAEDIEKVNKGIGIENIHYLNDGLWHMKTYKX   182
   MarmMcts2 -MFKKFDEKESVSNCIQLKTSVIKGIKSQLIEQFPGIEPWLNQIMPKKDPVKIVRCHEHTEILTVSGELLFFRQRKGPFCPTLRLLHKYPFILPHQQVDKGAIKFVLSGANIMCPGLTSPGAKLFPAAVDTIVAVTAEGKQHALCVGVMKMSAEDIEKVNKGIGIENIHYLNDGLWHMKTYKX   182
     MoMcts2 -MFKKFDEKESVSNCIQLKTSVIKGIKSQLTEQFPGIEPWLNQIMPKKDPVKIVRCHEHMEILTVNGELLFFRQRKGPFYPTLRLLHKYPFILPHQQVDKGAIKFVLSGANIMCPGLTSPGAKLYTAAVDTIVAVMAEGKEHALCVGVMKMAAADIEKINKGIGIENIHYLNDGLWHMKTYKX   182
   OranMcts2 -MFKKFDEKESVSNCIQLKTSVIKGIKSQLVEQFPGIEPWLNQIMPKKDPVKIVRCHEHTEILTVSGELLFFRQRKGPFCPTLRLLHKYPFILPHQQVDKGAIKFVLSGANIMCPGLTSPGAKLYPAAVDTIVAVTAEGKQHALCVGVMKMSAEDIEKVNKGIGIENIHYLNDGLWHMKTYKX   182
    RatMcts2 -MFKKFDEKESVSNCIQLKTSVIKGIKSQLTEQFPGIEPWLNQIMPKKDPVKIVRCHEHMEILTVNGELLFFRQRKGPFYPTLRLLHKYPFILPHQQVDKGAIKFVLSGANIMCPGLTSPGAKLYTAAVDTIVAVMAEGKEHALCVGVMKMAAADIEKINKGIGIENIHYLNDGLWHMKTYKX   182
     HuMcts2 -MFKKFDEKESVSNCIQLKTSVIKGIKSQLVEQFPGIEPWLNQIMPKKDPVKIVRCHEHTEILTVSGELLFFRQRKGPFCPTLRLLHKYPFILPHQQVDKGAIKFVLSGANIMCPGLTSPGAKLYPAAVDTIVAVTAEGKQHALCVGVMKMSAEDIEKVNKGIGIENIHYLNDGLWHMKTYKX   182
       ruler 1.......10........20........30........40........50........60........70........80........90.......100.......110.......120.......130.......140.......150.......160.......170.......180...
```

```
>P.troglodytesNap1l5
MADSENQGPAEPSQAAAA-----AEAAAEEVMAEGGAQGGDCDSAAGDP-----------
-------------------------DSAAGEMAEEPQTPAENAPKPKNDFIENLPNLVKR
PIMALKKLQKRCDKIEAKFDKEFQALEKKYNDIYKPLLAKIQELT-----GEMEGCAWTL
EGEEEE------------------EEEYEDDEEEGEDEEEEE-AAAEAAA----------
------------------------------------------------------------
------------------------------------------------------------
-----------------------------------------------------GAKHDDA
HAEMPDDAKKX-------------------------------------------------
------------------------------------------------------------
------------------------------------------------------------
----------------------------
>B.taurusNap1l5
MADSQNQGSAEPSQAAAAAAAADAAAAAEEVMAEGGAQGGDSDSASSDS-----------
-------------------------DGVVGQMAEEPQTPAENAPKPRNDFIESLPNSVKC
RVLALKKLQKRCDKIEAKFDKEFQALEKKYNDIYKPLLAKIQELT-----GEMEGCAWTL
EGDEEDD----------------DDDEYEDEEEGEEEDEEEEEPAAEAAG-T--------
------------------------------------------------------------
------------------------------------------------------------
----------------------------------------------------AAAKDEGP
HSAVPDDAKKX-------------------------------------------------
------------------------------------------------------------
------------------------------------------------------------
----------------------------
>C.l.familiarisNap1l5
MADSENQGPAEPSQA--------AAEAAEEVMAEGGAQGGDSDSAA--------------
-----------------------------GPTAEEPQTPADNAPKPKNDFIESLPNSVKC
RVLALKKLQKRCDKIEAKFDKEFQALEKKYNDIYKPLLAKIQELT-----GEMEGCAWTL
EGEEDDD----------------DEEEYEDEEEGEEEEEEEEEAAAEAAVEA--------
------------------------------------------------------------
------------------------------------------------------------
----------------------------------------------------AAAKDEGP
HSAVPDDAKK--------------------------------------------------
------------------------------------------------------------
------------------------------------------------------------
----------------------------
>H.sapiensNap1l5
MADSENQGPAEPSQAAAA-----AEAAAEEVMAEGGAQGGDCDSAAGDP-----------
-------------------------DSAAGQMAEEPQTPAENAPKPKNDFIESLPNSVKC
RVLALKKLQKRCDKIEAKFDKEFQALEKKYNDIYKPLLAKIQELT-----GEMEGCAWTL
EGEEEE------------------EEEYEDDEEEGEDEEEEE-AAAEAAA----------
------------------------------------------------------------
------------------------------------------------------------
-----------------------------------------------------GAKHDDA
HAEMPDDAKKX-------------------------------------------------
------------------------------------------------------------
------------------------------------------------------------
----------------------------
>C.jacchusNap1l5
MADSENQGPAEPSQAAAA-----AEA----------------------------------
---------------------------AAGQMAEEPQTPAENAPKPKNDFIESLPNSVKC
RVLALKKLQKRCDKIEAKFDKEFQALERKYNDIYKPLLAKIQELT-----GEMEGCAWTL
EGEEE------------------------------------------GGR----------
------------------------------------------------------------
------------------------------------------------------------
-----------------------------------------------------GVRGX--
------------------------------------------------------------
------------------------------------------------------------
------------------------------------------------------------
----------------------------
>M.musculusNap1l5
```

```
MADPEKQGPAE-------------SRAEDEVME--GAQGGE-DAATG-------------
-------------------------DSAAAPAAEEPQAPAENAPKPKKDFMESLPNSVKC
RVLALKKLQKRCDKIEAKFDKEFQALEKKYNDIYKPLLAKIQELT-----GEMEGCAWTL
EGEDDE----------------------DDEEEDDEEEEEEEEAAAGATG----------
------------------------------------------------------------
------------------------------------------------------------
------------------------------------------------------------
---GPNFAKKX-------------------------------------------------
------------------------------------------------------------
------------------------------------------------------------
----------------------------
>P.pygmaeusNap1l5
MADSENQGPAEPSQAAAA-----AEAAAEEVMAEGGAQGGDCDSAAGDP-----------
-------------------------DSAAGEMAEEPQTPAENAPKPKNDFIESLPNSVKC
RVLALKKLQKRCDKIEAKFDKEFQALEKKYNDIYKPLLAKIQELT-----GEMEGCAWTL
EGEEEE------------------EEEYEDDEEEGEEEEEEEEAAAEAAA----------
------------------------------------------------------------
------------------------------------------------------------
-----------------------------------------------------GAKHDDA
HAEMPDDAKKX-------------------------------------------------
------------------------------------------------------------
------------------------------------------------------------
----------------------------
>R.novergicusNap1l5
MADPEKQGPAE-------------SRAEDEVME--GAQGGE-DAATG-------------
-------------------------DSATAPAAEEPQAPAENAPKPKNDFIESLPNPVKC
RVLALKKLQKRCDKIEAKFDKEFQALEKKYNDIYKPLLAKIQELT-----GEMEGCAWTL
EGEDDE----------------------DDEEEEDEEEEEEE-AAAGATG----------
------------------------------------------------------------
------------------------------------------------------------
------------------------------------------------------------
---GPDSAEKX-------------------------------------------------
------------------------------------------------------------
------------------------------------------------------------
----------------------------
>M.mulattaNap1l5
MADSENQGPAEPSQAAAA-----AEAAAEEVMAEGGAQGGDCDSAAGDP-----------
-------------------------DSAAGQMAEEPQTPAENAPKPKNDFIESLPNSVKC
RVLALKKLQKRCDKIEAKFDKEFQALEKKYNDIYKPLLAKIQELT-----GEMEGCAWTL
EGEEEE------------------EEEYEDDEEEGEEEEEED-AAAEAAA----------
------------------------------------------------------------
------------------------------------------------------------
-----------------------------------------------------GAKHDDA
HAEMPDDAKKX-------------------------------------------------
------------------------------------------------------------
------------------------------------------------------------
----------------------------
>H.sapiensNapl13
MAEAD-FKMVSEPVAHGVAEEEMASSTSDSGEESDSSSSSSSTSDSSSSSSTSGSSS--G
SGSSSSSSGS---------TSSRSRLYRKKRVPEPSRRARRA--PLGTNFVDRLPQAVRN
RVQALRNIQDECDKVDTLFLKAIHDLERKYAELNKPLYDRRFQIINAEYEPTEEECEWNS
EDEEFSSDEEVQDNTPSEMPPLEGE-EEENPKENPEVKAEEKEVPKEIPEVKDEEKEVPK
EIPEVK------------------------------------------------------
----------------AEEKADSKDCMEATPEVKEDPKEVPQVKADDKEQPKATEAKARA
AVRETHKRVPEERLQDSVDLKRARKGKPKREDPKGIPDYWLIVLKNVDKLGPMIQKYDEP
ILKFLSDVSLKFSKPGQPVSYTFEFHFLPNPYFRNEVLVKTYIIKAKPDHNDPFFSWGWE
IEDCKGCKIDWRRGKDVTVTTTQSRT--TATGEIEIQPRVVPNASFFNFFSPPEIPMIGK
---LEPREDAILDEDFEIGQILHDNVILKSIYYYTGEVN--GTYYQFGKHYGNKKYRKX-
----------------------------
>C.jacchusNap1l3
MAEAD-FKEVSEPVAQSVAEEEMASSASDSGEESDSSSSSSSSTSSSSSSSSSSSSS--S
```

```
SGSSSTSSGS--------------GLYRKKRVPEPSRRARRA--PWRTDFVIKLPQAVRN
RVQALRNIQDECDKVDTMFLKAIHDLERKYAELNKPLYDRRFQIINAEYEPTEEECEWNS
EDEEFSSDEEVQDDTLSEMPPLEGE-EEEKPKENPEVKAEEKEVPKEIPEVKDEEKEVPK
EIPEVKDEEKEVPKEIPEVKDEEKEVPKEIAEVMDEEKVIPKEIAEVMDEEKVIPKEIAE
VMDEEKVVPKEIAEVKAEEKADSEDGMEAIPEVKEDPKEAPQAKAEDKEQPKATEAKPRA
GVREAHKRVPEERLPERVSLKRARRGKPKREDPKGIPDYWLIVLKNVDKLGPMIQKYDEP
ILKFLSDVNLKFSKPGQPVSYTFEFHFLPNPYFRNEVLVKTYIIKSKPDHHDPFFSWGWE
IEDCKGCKIDWRRGKDVTVKTIQSRT--TATG--EIQPRVVPNSSFFNFFSPPEIPMIGK
---LEPQEDAILDEDFEIGQILHDNVILKSIYYYTGEVNEYGSYYGVGKDYGNRKYRKX-
----------------------------
>M.musculusNap1l3
MAEAD-PKMVTEPGAHGVAEEAMASTACDSGDESDSNSSSSTNSCSSSGSSSSGSSSSSS
SSSSSSSSSSSSSSGSSGSSSNGSHLNRKKRVPEPSRRAQRR--PSGKLFLDKLPQAVRN
RVQALRNIQNECDKVDTLFLRAIHDLERKYAELNKPLYDKRFQIINAEYEPTEEECEWNS
E-EEFSGDEEMQDDTPNEMPPLEGEEEEEESCNEKAEVKEEGTHVPEEVPEAKVEEEEAPK
ETPEVKTEEKDIPKEGAEEKAEEQESSKE-------------------------------
-----------IPEVKGEEKADSTDCIDIAPEEKEDVKEVTQANTENKDQP-TEEFTPRA
PAREAQKRVPETRPEEGVNIKRARKGKPKKEDPKGIPDYWLTVLKNVDKLGPMIQKCDEP
ILKFLSDVSLKFSNPGQPIGYTFEFHFLPNPYFRNELLMKTYIIRSKPDHYDPFFAWGWE
IEECKGCKIDWRRGKDVTVTTTRSRP--GITGEIEVQPRVVPNASFFNFFSPPEIPLIGK
---LEPREDAILDEDFEIGQILHDNVILKSIYYFTGEIN--DPYYHDFRDYGNRKYYKX-
----------------------------
>P.pygmaeusNap1l3
MAEAD-FKMVSEPVAHGVAEEEMASSTSDSGEESDSSSSSSSTSGSSSSSSTSGSSSSSG
SGSSSSSSGSGS-------TSSRSRLYRKKRVPEPSRRARRA--PLGTNFVDRLPQAVRN
RVQALRNIQDECDKVDTLFLKAIHDLERKYAELNKPLYDRRFQIINAEYEPTEEECEWNS
EDEEFSSDEEVQDNTPSEMPPLEGE-EEENPKENPEVKAEEKEVPKEIPEVKDEEKEVPK
EIPQVK------------------------------------------------------
----------------AEEKADSKDCMEATPEVNEDPKEAPQVKADDKEQPKATEAKARA
AVREAHKRVPEERLQDSVDLKRARKGKPKREDPKGIPDYWLIVLKNVDKLGPMIQKYDEP
ILKFLSDVSLKFSKPGQPVSYTFEFHFLPNPYFRNEVLVKTYIIKSKPDHNDPFFSWGWE
IEDCEGCKIDWRRGKDVTVTTTQSRT--TATGEIEIQPRVVPNASFFNFFSPPEIPMIGK
---LEPREDAILDEDFEIGQILHDNVILKSVYYYTGEVN--GTYYQFGKHYGNKKYRKX-
----------------------------
>R.novergicusNap1l3
MAEAD-PKMVTEPGAHGVAEEAMASTACDSGDESDSNSSSSTNSCSSSGSSSSGSSSSSS
SSSSSSSSSSSSSSGSSGSSSNGSHLNRKKRVPEPSRRAQRR--PSGKLFLDKLPQAVRN
RVQALRNIQNECDKVDTLFLRAIHDLERKYAELNKPLYDKRFQIINAEYEPTEEECEWNS
E-EEFSGDEEMQDDTPNEMPPLEGEEEEEESCNEKAEVKEEGTHVPEEVPEAKVEEEEAPK
ETPEVKTEEKDIPKEGAEEKAEEQESSKE-------------------------------
-----------IPEVKGEEKADSTDCIDIAPEEKEDVKEVTQANTENKDQP-TEEFTPRA
PAREAQKRVPETRPEEGVNIKRARKGKPKKEDPKGIPDYWLTVLKNVDKLGPMIQKCDEP
ILKFLSDVSLKFSNPGQPIGYTFEFHFLPNPYFRNELLMKTYIIRSKPDHYDPFFAWGWE
IEECKGCKIDWRRGKDVTVTTTRSRP--GITGEIEVQPRVVPNASFFNFFSPPEIPLIGK
---LEPREDAILDEDFEIGQILHDNVILKSIYYFTGEIN--DPYYHDFRDYGNRKYYKX-
----------------------------
>P.troglodytesNap1l3
MAEAD-FKMVSEPVARGVAEEEMASSTSDSGEESDSSSSSSSTSDSSSSSSTSGSSS--G
SGSSSSSSGS---------TSSRSRLYRKKRVPEPSGRARRA--PLGTNFVDRLPQAVRN
RVQALRNIQDECDKVDTLFLKAIHDLERKYAELNKPLYDRRFQIINAEYEPTEEECEWNS
EDEEFSSDEEVQDNTPSEMPPLEGE-EEENPKENPEVKAEEKEVPKEIPEVKDEEKEVPK
EIPEVK------------------------------------------------------
----------------AEEKADSKDCMEATPEVKEDPKEVPQVKADDKEQPKATEAKARA
AVREAHKRVPEERLQDSVDLKRARKGKPKREDPKGIPDYWLIVLKNVDKLGPMIQKYDEP
ILKFLSDVSLKFSKPGQPVSYTFEFHFLPNPYFRNEVLVKTYIIKSKPDHNDPFFSWGWE
IEDCKGCKIDWRRGKDVTVTTTQSRT--TATGEIEIQPRVVPNASFFNFFSPPEIPMIGK
---LEPREDAILDEDFEIGQILHDNVILKSIYYYTGEVN--GTYYQFGKHYGNKKYRKX-
----------------------------
>C.l.familiarisNap1l3
MAEED-LNMVSEPAAQRVTEEKMASSSSDSGEESDSNSSSSSTSCSSSS----------G
SGRS--------------------RLYRKKRVSGPSRGARGA--PLGKSFVDRLPQAVRN
```

```
RVQALRNIQDECDKVDILFLKAIHDLERKYAELNKPLYDRRFQIINAEYEPTEEEYEWNS
EDEVFSSDEEVQEDSPIEMPALESEQEDDNPKEKPQVKAEENEVPKEIPEAKTEEN----
------------------------------------------------------------
--------------------AESKDFLGIKPEVKEEPEVVPQQNAEDQKQPKAAEAKAKA
AVIEAPKRISEVRPKERVNLKRARKGKPKKEDPKGIPDYWLTVLKNVDKLGPMIQKYDAP
ILKFLSDISLKFSKPGQPISYTFEFYFLPNPYFRNEMLTKTYIIKSKPDHNDPFFSWGWE
IQDCKGCKIDWRRGKDVTVTTTQSRT--TASGEIEIQPRVVANASFFNFFSPPEIPKIGK
---LEPREDAILDEDFEIGQILHDNVILKSIYYYTGEVK--GTYDD-GKDYGNRKYRKX-
----------------------------
>P.troglodytesNap1l2
MAESENHKELSESSQEEAGNQIMVEGLGEHLERGEDAAAGLGDDGKCGEEAAAGLGEEGE
NGEDTAAGSG----------EDGKKGGDTDEDSEADRPKGLIGYVLDTDFVESLPVKVKY
RVLALKKLQTRAANLESKFLREFHDIERKFAEMYQPLLEKRRQIINAIYEPTEEECEYKS
DSEDCDDEEMCH----EEMYGNEEGMVHEYVDEDDGYEDYYYDYAVEEEEEE--------
------------------------------------------------------------
------------------------------------------------------------
------------EEEE--DIEATGEENKEEEDPKGIPDFWLTVLKNVDTLTPLIKKYDEP
ILKLLTDIKVKLSDPGEPLSFTLEFHFKPNEYFKNELLTKTYVLKSKLAYYDPHPYRGTA
IEYSTGCEIDWNEGKNVTLKTIKKKQKHRIWGTIRTVTEDFPKDSFFNFFSPHGITSNGR
----DGND------DFLLGHNLRTYIIPRSVLFFSGDALE-SQQEGVVREVNDAIYDKII
YDNWMAAIEEVKACCKNLEALVEDIDRX
>C.l.familiarisNap1l2
MAESADHKKLLEFSQEEADNKVIMEGPGEQPEQSEDVAAGPGDDKERGEEAAVGPGKEGG
KGEDAAAGSG----------EGGVKDEDIDEDSD--RPKGLIGYLLDTDFVESLPLKVKY
RVLALKKLQTRVANLESKFMREFHGIERKFAEMYQPLLEKRRQIINAIYEPTKEECEYKS
DSEDYDD-EMYDE---EEMYGNEEGLVHEYMDEDDGYEGDYYDYAVEEDDGD--------
------------------------------------------------------------
------------------------------------------------------------
----------DDDDDNGDDIEATGKENKEEQDPKGIPDFWLTVLKNVDTLTPLIKKYDEP
ILKLLTDIKVKLSGPGEPLSFTLEFHFKPNEYFKNELLTKTYVLKSRLAYYDPHPYRGTA
IEYCTGCEIDWNEGKNVTLKTIRKKQKHRIWGTVRTVTEDFPKDSFFNFFTPQGISSNGK
----DGND------DFLLGHNLRTYIIPRSVLFFSGDALE-SQQEGVVREVNDAIYDKII
YDNWMAAIEEVKACCKNLETIVEDIDRX
>E.caballusNap1l2
MAESADYKELLESSQEEAGNKVMMEGPREQPE--------------RGEEAAAGPGEEGE
RGEDAAAGSG----------EGGEKGEDTDEDSDPDRPKGLTGYLLDTDFVESLPVKVKY
RVLALKKLQTRVANLESKFLREFHDIERKFAELYQPLLEKRRQIINAIYEPTKEECAYKS
DSEDYDEEEMYDE---EEMYGNEESLVHEYMDEGDGYEEDYYDYDVEEEEDDE-------
------------------------------------------------------------
------------------------------------------------------------
----------DDDDDDDDDIEATREENKEE-DPKGIPDFWLTVLKNVDTLTPLIKKYDEP
ILKLLTDIKVKLSDPGEPLSFTLEFHFKPNEYFKNELLTKTYVLKSRLAFYDPHPYRGTA
IEYCTGCEIDWNEGKNVTLKTIKKKQKHRIWGTIRTVTEDFPKDSFFNFFTPHGISSNGK
----DGND------DFLLGHNLRTYIIPRSVLFFSGDALE-SQQEGVVREVNDAIYDKII
YDNWMAAIEEVKACCKNLETIVEDIDRX
>H.sapiensNap1l2
MAESENRKELSESSQEEAGNQIMVEGLGEHLERGEDAAAGLGDDGKCGEEAAAGLGEEGE
NGEDTAAGSG----------EDGKKGGDTDEDSEADRPKGLIGYVLDTDFVESLPVKVKY
RVLALKKLQTRAANLESKFLREFHDIERKFAEMYQPLLEKRRQIINAIYEPTEEECEYKS
DSEDCDDEEMCH----EEMYGNEEGMVHEYVDEDDGYEDYYYDYAVEEEEEE--------
------------------------------------------------------------
------------------------------------------------------------
------------EEED--DIEATGEENKEEEDPKGIPDFWLTVLKNVDTLTPLIKKYDEP
ILKLLTDIKVKLSDPGEPLSFTLEFHFKPNEYFKNELLTKTYVLKSKLAYYDPHPYRGTA
IEYSTGCEIDWNEGKNVTLKTIKKKQKHRIWGTIRTVTEDFPKDSFFNFFSPHGITSNGR
----DGND------DFLLGHNLRTYIIPRSVLFFSGDALE-SQQEGVVREVNDAIYDKII
YDNWMAAIEEVKACCKNLEALVEDIDRX
>C.jacchusNap1l2
MAESADHKELSESSQEEAGNQIMMEGPGEQPERGEDAAAGLGDDGKCGDEAAAGLGEEGE
KGEDTAAGSG----------EDGKKGGDTDEDLDPDRPKGLIGYVLDTDFVESLPVKVKY
RVLALKKLQTRAANLESKFLREFHDIERKFAEMYQPLLEKRRQIINAIYEPTEEECEYKS
```

```
DSEDYDDEEMYD----EEMYGNEEGMVHEYVDEDDGYEDYYYDYAVEEEEDD--------
------------------------------------------------------------
------------------------------------------------------------
------------DDDDDDDIEATGEENKEEEDPKGIPDFWLTVLKNVDALTPLIKKYDEP
ILKLLTDIKVKLSDPGEPLSFTLEFHFKPNEYFKNELLTKTYVLKSKLAYYDPHPYRGTA
IEYSTGCEIDWNEGKNVTLKTIKKRQKHRIWGTIRTVTEDFPKDSFFNFFSPHGFTSNGR
----DGND------DFLLGHNLRTYIIPRSVLFFSGDALE-SQQEGVVREVNDEIYDKII
YDNWMAAIEEVKACCKNLEALVEDIDRX
>M.musculusNap1l2
MAESVDHKELSESNQEELGSQVMAEGPGESQDRSEGVSIEPGDGGQHGEETVAAGVGEEG
KGEEAAAGSG----------EDAGKCGGTDEDSDSDRPKGLIGYLLDTDFVESLPVKVKC
RVLALKKLQTRAAHLESKFLREFHDIERKFAEMYQPLLEKRRQIINAVYEPTEEECEYKS
DCEDYFEEEMDEE---EETNGNEDGMVHEYVDEDDGYEDCYYDYDDEEEEEE--------
------------------------------------------------------------
------------------------------------------------------------
--------------EDD-SAGATGGEEVNEEDPKGIPDFWLTVLKNVEALTPMIKKYDEP
ILKLLTDIKVKLSDPGEPLSFTLEFHFKPNEYFKNELLTKTYVLKSKLACYDPHPYRGTA
IEYATGCDIDWNEGKNVTLRTIKKKQRHRVWGTVRTVTEDFPKDSFFNFFSPHGISLNGG
----DEND------DFLLGHNLRTYIIPRSVLFFSGDALE-SQQEGVVREVNDEIYDKII
YDDWMAAIEEVKACCKNLEALVEDIDRX
>R.novergicusNap1l2
MAESAEHKELSDSNQEELGSQVMAEGPGGSQDCSEGVSTEPGDGGQQGEETVAAGVEEEG
KGEEAAAGSG----------EDAGKRGGAAEDSDSDRPKGLIGYLLDTDFVESLPVKVKY
RVLALKKLQTRAAHLESKFLREFHDIERKFAEMYQPLLEKRRQIINAVYEPTEEECEYKS
DCEDYFEEEMDEE---EETNGNEEGMVHEYVDEDDGYEDCYYDYDDEEEEEE--------
------------------------------------------------------------
------------------------------------------------------------
--------------EEDESAGATGGEAVNEEDPKGIPDFWLTVLKNVEALTPMIKKYDEP
ILKLLTDIKVKLSDPGEPLSFTLEFHFKPNEYFKNELLTKTYVLKSKLACYDPHPYRGTA
IEYATGCHIEWNEGKNVTLRTIKKKQRHRVWGTVRTVTEDFPKDSFFNFFSPHGISLNGG
----DEND------DFLLGHNLRTYIIPRSVLFFSGDALE-SQQEGVVREVNDEIYDKII
YDDWMAAIEEVKACCKNLEALVEDIDRX
>M.mulattaNap1l2
MAESANHKELSESSQQEAGNQIMVEGPGEHPERGEDAAAGLGDDGKCGEEAAAGLGEEGE
NGEDTAAGSG----------EDGKKGEDTDKDSDPDRPKGLIGYVLDTDFVESLPVKVKY
RVLALKKLQTRAANLESKFLREFHDIERKFAEMYQPLLEKRRQIINAIYEPTEEECEYKS
DSEDYDDEEMCD----EEMYGNEEGMVHEYVDEDDGYEDYYYDYAVEEEEEE--------
------------------------------------------------------------
------------------------------------------------------------
------------EDEDEDDIEATGEENKEEEDPKGIPDFWLTVLKNVDTLTPLIKKYDEP
ILKLLTDIKVKFSDPGEPLSFTLEFHFKPNEYFKNELLTKTYVLKSKLAYYDPHPYRGTA
IEYSTGCEIDWNEGKNVTLKTIKKKQKHRIWGTIRTVTEDFPKDSFFNFFSPHGITSNGR
----DGND------DFLLGHNLRTYIIPRSVLFFSGDALE-SQQEGVVREVNDAIYDKII
YDNWMAAIEEVKACCKNLEALVEDIDRX
>P.troglodytesNap1l1
MADID-NKEQSELDQDLDDVEEVEE--EETGEETKLKARQLTVQMMQ-------------
--------------------------NPQILAALQERLDGLV--ETPTGYIESLPRVVKR
RVNALKNLQVKCAQIEAKFYEEVHDLERKYAVLYQPLFDKRFEIINAIYEPTEEECEWKP
D---------------------EEDEISEELKEKAKIEDEKKDEEKE-------------
------------------------------------------------------------
------------------------------------------------------------
-------------------------------DPKGIPEFWLTVFKNVDLLSDMVQEHDEP
ILKHLKDIKVKFSDAGQPMSFVLEFHFEPNEYFTNEVLTKTYRMRSEPDDSDPFSFDGPE
IMGCTGCQIDWKKGKNVTLKTIKKKQKHKGRGTVRTVTKTVSNDSFFNFFAPPEVPESGD
---LDDDAEAILAADFEIGHFLRERIIPRSVLYFTGEAIEDDDD-DYDEEGEEADEEGEE
EGDEENDPDYDPKKDQNPAECKQQX---
>H.sapiensNap1l1
MADID-NKEQSELDQDLDDVEEVEE--EETGEETKLKARQLTVQMMQ-------------
--------------------------NPQILAALQERLDGLV--ETPTGYIESLPRVVKR
RVNALKNLQVKCAQIEAKFYEEVHDLERKYAVLYQPLFDKRFEIINAIYEPTEEECEWKP
D---------------------EEDEISEELKEKAKIEDEKKDEEKE-------------
```

```
------------------------------------------------------------
------------------------------------------------------------
-------------------------------DPKGIPEFWLTVFKNVDLLSDMVQEHDEP
ILKHLKDIKVKFSDAGQPMSFVLEFHFEPNEYFTNEVLTKTYRMRSEPDDSDPFSFDGPE
IMGCTGCQIDWKKGKNVTLKTIKKKQKHKGRGTVRTVTKTVSNDSFFNFFAPPEVPESGD
---LDDDAEAILAADFEIGHFLRERIIPRSVLYFTGEAIEDDDD-DYDEEGEEADEEGEE
EGDEENDPDYDPKKDQNPAECKQQX---
>P.troglodytesNap1l4
MADHS-FSDG----VPSDSVEAAKN--ASN-------TEKLTDQVMQ-------------
--------------------------NPRVLAALQERLDNVP--HTPSSYIETLPKAVKR
RINALKQLQVRCAHIEAKFYEEVHDLERKYAALYQPLFDKRREFITGDVEPTDAESEWHS
ENE-------------------EEEKLAGDMKNKVVVTEKEAATAEEP------------
------------------------------------------------------------
------------------------------------------------------------
-------------------------------NPKGIPEFWFTIFRNVDMLSELVQEYDEP
ILKHLQDIKVKFSDPGQPMSFVLEFHFEPNDYFTNSVLTKTYKMKSEPDKADPFSFEGPE
IVDCDGCTIDWKKGKNVTVKTIKKKQKHKGRGTVRTITKQVPNESFFNFFNPLKGNSSGD
GESLDEDSEFTLASDFEIGHFFRERIVPRAVLYFTGEAIEDDDNFEEGEEGEEEELEGDE
EGEDEDDAEINPKV--------------
>M.domesticusNap1l4
MELFRRCSFRFLXNCXSXQRKTYRPGDAESPSASSSPGAAX-------------------
--------------------------ECVPHAFELHXNLTQSGQETHXCFETAAGEVCTH
RSQVLRGSAXFGAKIRSPLPASLX-XETGFCHWXCXTYRCRIRMAQX--EXGGGETGWRS
EKQSGDS---------------REGSSCRRAKSQRDSRILVYNLXKRGYAER--------
------------------------------------------------------------
------------------------------------------------------------
----------------------------------------------------ISAGVXXA
DFETPAGYQSQVLXPWAAHVLLVRVPLXAQRLLYQFCPDXDLQDEIRARHHRPLLLRRPG
NSRLXRVHYRVEEGQERHRENNQEKAETXGPWHREDSHXASPQRFLFXLLXPHQSIWGWR
VAGXRFRIYISFRLXNWTLLPXEDSSTCRPLFYWRSHRGXXXFXRRRGRGRRGKKWQRDP
FPGRGPQSRPRERVRPACRVQAA-----
>H.sapiensNap1l4
MADHS-FSDG----VPSDSVEAAKN--ASN-------TEKLTDQVMQ-------------
--------------------------NPRVLAALQERLDNVP--HTPSSYIETLPKAVKR
RINALKQLQVRCAHIEAKFYEEVHDLERKYAALYQPLFDKRREFITGDVEPTDAESEWHS
ENE-------------------EEEKLAGDMKSKVVVTEKAAATAEEP------------
------------------------------------------------------------
------------------------------------------------------------
-------------------------------DPKGIPEFWFTIFRNVDMLSELVQEYDEP
ILKHLQDIKVKFSDPGQPMSFVLEFHFEPNDYFTNSVLTKTYKMKSEPDKADPFSFEGPE
IVDCDGCTIDWKKGKNVTVKTIKKKQKHKGRGTVRTITKQVPNESFFNFFNPLK--ASGD
GESLDEDSEFTLASDFEIGHFFRERIVPRAVLYFTGEAIEDDDNFEEGEEGEEEELEGDE
EGEDEDDAEINPKV--------------
```

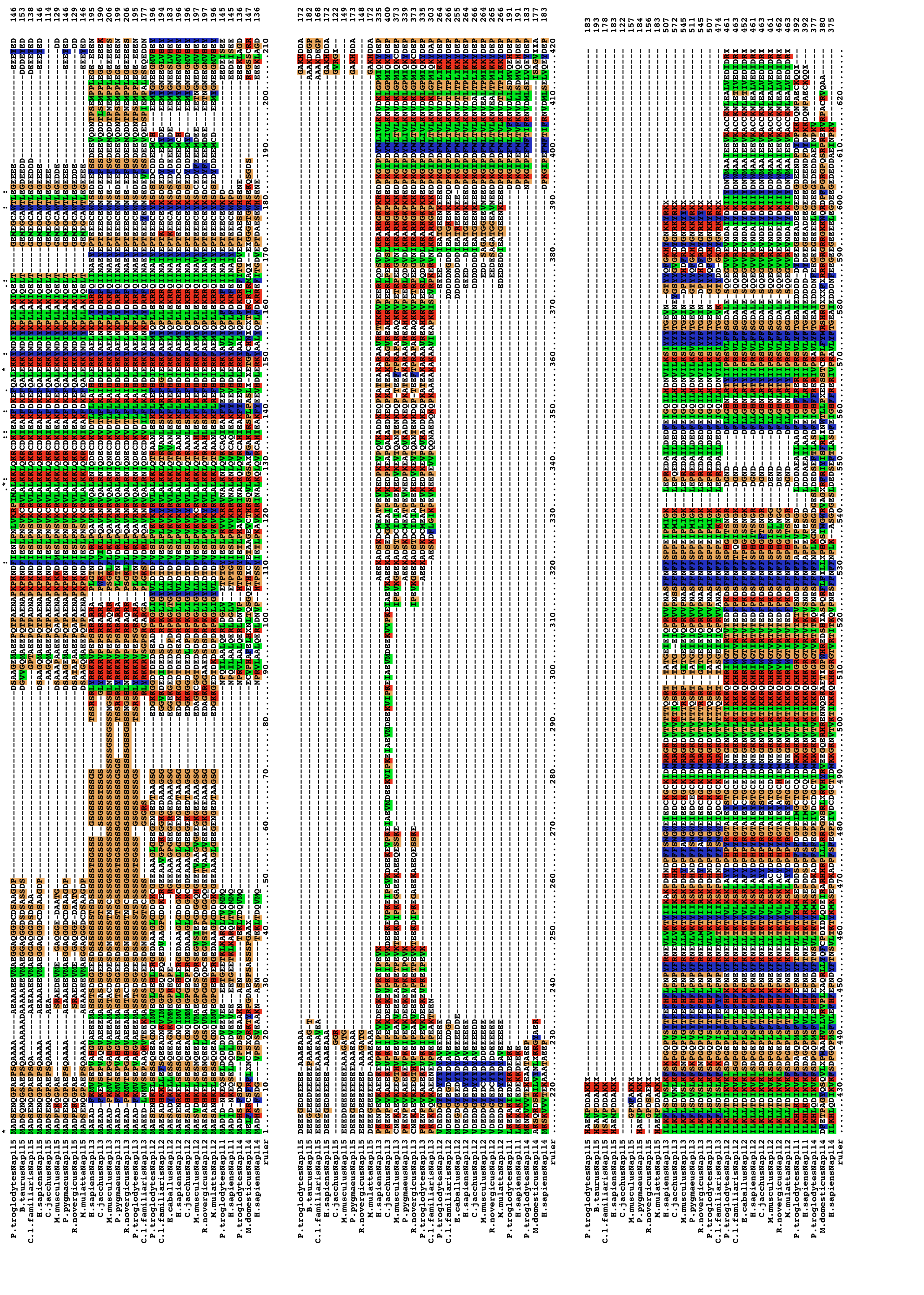

```
>ChimpU2af1-rs2
----------MTFPEKPSHKKYRAALKKEKRKKRRQELARLRDSGLSQKEEEEDTFIEEQ
QLEEEKLLERERQRLHEEWLLREQKAQEEFRIKKEKEEAAKKRQEEQERKLKEQWEEQQR
KEREEEEQKQQEKKEKEEALQKMLDQAENELENGTTWQNPEPPVDFRVMEKDRANCPFYS
KTGACRFGDRCSRKHNFPTSSPTLLIKSMFTTFGMEQCRRDDYDPDASLEYSEEETYQQF
LDFYEDVLPEFKNVGKVIQFKVSCNLEPHLRGNVYVQYQSEEECQAALSLFNGRWYAGRQ
LQCEFCPVTRWKMAICGLFEIQQCPRGKHCNFLHVFRNPNNEFWEANRDIYLSPDRTGSS
FGKNSERRERMGHHDDYYSRLRGRR-NPSPDHSYKRNGESERKR-SSHRGKK-SHKRTSK
SRERHNSRSRGRNRDRS-----------RDRSRG-------RGSRSRSR----RSRRSRS
QSSSRSRSRGRRRSGNRDRTVQSPKSKX--------------------------------
---------------------------------------------
>DogU2af1-rs2
----MAAPEKMMFPEKPSHKKYRAALKKAKRKKRRQELARLRDSGLLQKEEE-DAFIEEQ
RLEEEKLLEIERQKLHEAWLLREQKAQEEFRRKKEKEEAARKRQEEQERKLKEEWEEQQR
REREEEEQKLQEKRERE-------------LENGATWQNPEPPIDLRIMEKDRANCPFYS
KTGACRFGDRCSRKHNFPSSSPTLLIKSMFTTFGMEQCRRDDYDPDASLEYSEEETYQQF
LDFYDDVLPEFKNVGKVIQFKVSCNLEPHLRGNVYVQYQSEEECQAALSLFNGRWYAGRQ
LQCEFCPVTRWKMAICGLFEIHQCPRGKHCNFLHVFRNPNNEFWEANRDIYLSPDRTGSS
FGKGSERRERTGHHDEYYGRPRRRR-SPSPVHSYKRNGEAERKRRSSHRGKK-SHKHLSR
SRDGPSSRSRGRK---------------RGRSRG-------RG--------------SRS
QSSSRSRSRGRRRSGSRDX-----------------------------------------
---------------------------------------------
>HumanU2af1-rs2
----MAAPEKMTFPEKPSHKKYRAALKKEKRKKRRQELARLRDSGLSQKEEEEDTFIEEQ
QLEEEKLLERERQRLHEEWLLREQKAQEEFRIKKEKEEAAKKRQEEQERKLKEQWEEQQR
KEREEEEQKRQEKKEKEEALQKMLDQAENELENGTTWQNPEPPVDFRVMEKDRANCPFYS
KTGACRFGDRCSRKHNFPTSSPTLLIKSMFTTFGMEQCRRDDYDPDASLEYSEEETYQQF
LDFYEDVLPEFKNVGKVIQFKVSCNLEPHLRGNVYVQYQSEEECQAALSLFNGRWYAGRQ
LQCEFCPVTRWKMAICGLFEIQQCPRGKHCNFLHVFRNPNNEFWEANRDIYLSPDRTGSS
FGKNSERRERMGHHDDYYSRLRGRR-NPSPDHSYKRNGESERKS-SRHRGKK-SHKRTSK
SRERHNSRSRGRNRDRS-----------RDRSRG-------RGSRSRSRSRSRRSRRSRS
QSSSRSRSRGRRRSGNRDRTVQSPKSKX--------------------------------
---------------------------------------------
>MarmosetU2af1-rs2
----MAAPEKMTFPEKPSHKKYRAALKKEKRKKRRQELARLRDSGLSQKEEEEDTFIEEQ
QLEEEKLLERERQRLHEEWLLREQKAQEEFRIKKEKEEAARKRQEEQERKLKEQWEEQQR
KEREEEEQKRQEKREKEEAVQKMLDRAENELENGTTWQNPEPPVDFRVMEKDRANCPFYS
KTGACRFGDRCSRKHNFPTSSPTLLIKSMFTTFGMEQCRRDDYDPDASLEYSEEETYQQF
LDFYEDVLPEFKNVGKVIQFKVSCNLEPHLRGNVYVQYQSEEECQAALSLFNGRWYAGRQ
LQCEFCPVTRWKMAICGLFEIQQCPRGKHCNFLHVFRNPNNEFWEANRDIYLSPDRTGSS
FGKNSERRERMGHHDEYYSRLRGRR-NPSPDHSYKRNGESERKR-SSHRGKK-SHKRTSK
SRERHSSRSRGRN---------------RDRSRG-------RGSRSQSR----RSRRSRS
QSSSRSRSRGRRRSGNKDRTVQSPKSKX--------------------------------
---------------------------------------------
>MouseU2af1-rs2
METAGATADATAGPQKLSRKKYLALRKKERRKRRRQALARLREAELAQKEEEEDPLAEEK
RLEEERLLEEERQRLHEEWLLREEKAQEEFRAKKKKEEEARKRKEELERKLKAEWEEQQR
KEREEEEQKRQEKREREEAVQKMLDQAENELENGGTWQNPEPPMDIRVLEKDRANCPFYS
KTGACRFGDRCSRKHNFPTSSPTLLIKGMFTTFGMEQCRRDDYDPDSSLEFSEEEIYQQF
LDFYYDVLPEFKSVGKVIQFKVSCNLEPHLRGNVYVQYQSEEDCQAAFSVFNGRWYAGRQ
LQCEFCPVTRWKMAICGLFEVQQCPRGKHCNFLHVFRNPNNEYRDANRDLYPSPDWTSSS
FGKNSERRERASHYDEYYGRSRRRRRSPSPDF-YKRNGESDRKSSSRHRVKK-SHRYGMK
SRERRSSPSRRRK-DHSPGPWSQSRRSHRSRSRSRSRSRSRSRTRSRSRGRGR--SRSRS
RSRSRGRSRSRGRGSGRGRGRGRGRGRNQSRSWSQSRSRSSSSSRSRSRGRR-------S
GSRDKTTQSPKSKX-------------------------------
>OpposumU2af1-rs2
----------------------------------------MKVDDLPEKDEEEEESIV-E
LEEEEKQLEAERQKLHEEWLLREQKAQEEFKLKKEKEEAAKRRQEEEERKIKEEWEEQQR
KEKQEEEQRQQEKRDREEAVQKMLDQAESQLENGVTWHNPEPPVDLRATEKDQANCPFYI
KTGSCRFGDRCSRKHNYPTSSQTLLIRSMFITFGMEQCRRDDYDTDASLEYSEEETYQQF
LDFYEDVLPEFKNVGKVIQFKVSCNFEPHLRGNVYVQYQSEEECQAAFSLFNGRWYAGRQ
```

```
LQCEFSPVTRWKMAICGLFGRQKCPRGKHCNFLHVFRNPNNEFWEANRDIHMSPDWANTS
FGKSSERRDRTSYHEEYYSRSRRRR-SPSPDHSYKRNGESERKKSNHHRSKKKSHKHSSK
SHERRRSRSRGRK---------------RDRSRR-------SRSQSRSR-------RSQS
KSASRSRSRDRKRSTSRDRNTX--------------------------------------
---------------------------------------------
>RatU2af1-rs2
---------MVAG----SRKKYMALRKKEKRKKRRQELARLRDAELSQKEEEEDPLGEEK
RLEEERLLEAERQRLHEEWLLREEKAQEEFRAKKRKEEAARKRKEELERKLKAEWEEQQR
KEREEEEQKQQEKRERE-------------LENGGTWQNPEPPTDIRVLEKDRANCPFYS
KTGACRFGDRCSRKHNFPTSSPTLLIKSMFTTFGMEQCRRDDYDPDSSLEYSEEETYQQF
LDFYYDVLPEFKSVGKVIQFKVSCNLEPHLRGNVYVQYQSEEDCQAAFSVFNGRWYAGRQ
LQCEFCPVTRWKMAICGLFEVQQCPRGKHCNFLHVFRNPNNEYREANRDIYLSPDWTSSS
FAKSSERRERASHYDEYYGRSRRRR-SPSPGLSYKRNGESDRKSSSNHRVKK-SHKHGMK
NRERHSSRSRRRKRDHSLGPWSQSRRSRHSRSRSHSQSRSRSSSRSRSCGRGRGWSHSRS
HSRSRKRSRNRSRSRSRNRNRNRNRKRNQSRSWSHSHSHSRSSSRSRSPGRRRSANMDMG
GSDHLQCEMPQEVISKGGQLCQSNFFKNTTWGFSFLWRENVRLWX
>RhesusU2af1-rs2
----------MTFPEKPSHKKYRAALKKEKRKKRRQELARLRDSGLSQKEEEEDTFIEEQ
QL-EEKLLERERQRLHEEWLLREQKAQEEFRIKKEKEEAAKKRQEEQERKLKEQWEEQQR
KEREEEEQKRQEKKEKEEALQKMLDQAENELENGTTWQNPEPPVDFRVMEKDRANCPFYS
KTGACRFGDRCSRKHNFPTSSPTLLIKSMFTTFGMEQCRRDDYDPDASLEYSEEETYQQF
LDFYEDVLPEFKNVGKVIQFKVSCNLEPHLRGNVYVQYQSEEECQAALSLFNGRWYAGRQ
LQCEFCPVTRWKMAICGLFEIQQCPRGKHCNFLHVFRNPNNEFWEANRDIYLSPDRTGSS
FGKNSERRERMGHHDDYYSRLRGRR-NPSPDHSYKRNGESERKR-SSHRGKK-SHKRTSK
SRERHNSRSRGRNKDRS-----------RDRSRG-------RGSRSRSRSR--RSRRSRS
QSSSRSRSRGRRRSGNKDRTVQSPKSKX--------------------------------
---------------------------------------------
>platypusU2af1-rs2
----MAAPAARDLAGKLSHRQYRAALKKEKRKKRRQELARLRDSGFAEKDEENSVAED-E
ELEEEKKLEAERQKLHEEWLLREQKAQEEFRLKKEKEEAAKRRQEEEERRIKEEWEEQQR
KEREEEEQKLQKKRDREEAVQKMLDQAESQLENGVTWHNPEPPADVRITEKDRANCPFYI
KTGSCRFGDRCSRKHNYPTLSPTLLIRSMFITFGMEQCRRDDYDTDASLEYSEEETYQQF
LDFYEDVLPEFKNVGKVIQFKVSCNFEPHLRGNVYVQYQSEEECQEAFSLFNGRWYAGRQ
LQCEFCPVTRWKMAICGLFERQKCPRGKHCNFLHVFRNPNNEFWEANRDIHMSPDRTSQS
FGRSSERRDRPSHYEDYHGRSRRRR-SPSPDYSYKRNGESERKKRSSHRSSK-YHKSSSR
SSDKRSSHSRGRK---------------RDRSR------------SRSR-------RSQS
RSPSRSRSRGRRRSASRERNPQSPKSKX--------------------------------
---------------------------------------------
>mouseU2af1-rs1
-----MASRQTAIPEKLSRKQYKAAMKKEKRKKRRQKMARLRALEAPPE-EDDDVSAN--
EELAERLLEIERQRLHEEWLLREEKAQEEFRIKKKKEEAARKQKEEQERQIKAEWEEQQK
KQREEEEQKLQEKREREEAVQKMLDQA----ENERIWQNPEPPKDLR-LEKYRPSCPFYN
KTGACRFGNRCSRKHDFPTSSPTLLVKSMFTTFGMEQCRRDDYDSDANLEYSEEETYQQF
LDFYHDVLPEFKNVGKVIQFKVSCNLEPHLRGNVYVQYQSEEECQAALSLFNGRWYAGRQ
LQCEFCPVTRWKVAICGLFEMQKCPKGKHCNFLHVFRNPNNEFREANRDIYMSPPAWTGS
SGKNSDRRERKDHHEEYYSKSRSYH--SGSYHSSKRNRESERKS--PHRWKK-SHKQTTK
SHERHSSR-RGRE----------------------------------------------E
DSSPGPQSQSHRTX----------------------------------------------
---------------------------------------------
>RatU2af1-rs1
-----MASQQTAFPEKLSRKQYKAAMKKEKRKKRRQKMARLRALEAPPEVEEEDVSAN--
EELAERLLEIERQKLHEEWLLREEKAQEEFRIKKKKEEAARKQKEEQERQIKAEWEEQQK
KQREEEEQKLQEKKEREEAVQKMLDQA----ENEGTWQNPEPPKDLR-LEKYRPSCPFYN
KTGACRFGNRCSRKHDFPTSSPTLLVKSMFTTFGMEQCRRDDYDSDANLEYSEEETYQQF
LDFYHDVLPEFKNVGKVIQFKVSCNLEPHLRGNVYVQYQSEEECQAALSLFNGRWYAGRQ
LQCEFCPVTRWKVAICGLFEMQKCPKGKHCNFLHVFRNPNNEFRDANRDIYL-PPASTGS
SGKNSDRGDRKDHREESYSKSRSHH--SGSYHSSKRSRESERKS--PHRWKK-SHKQATK
SHERHSSR-RGRE----------------------------------------------E
GSSPGPQSQSHRTX----------------------------------------------
---------------------------------------------
```

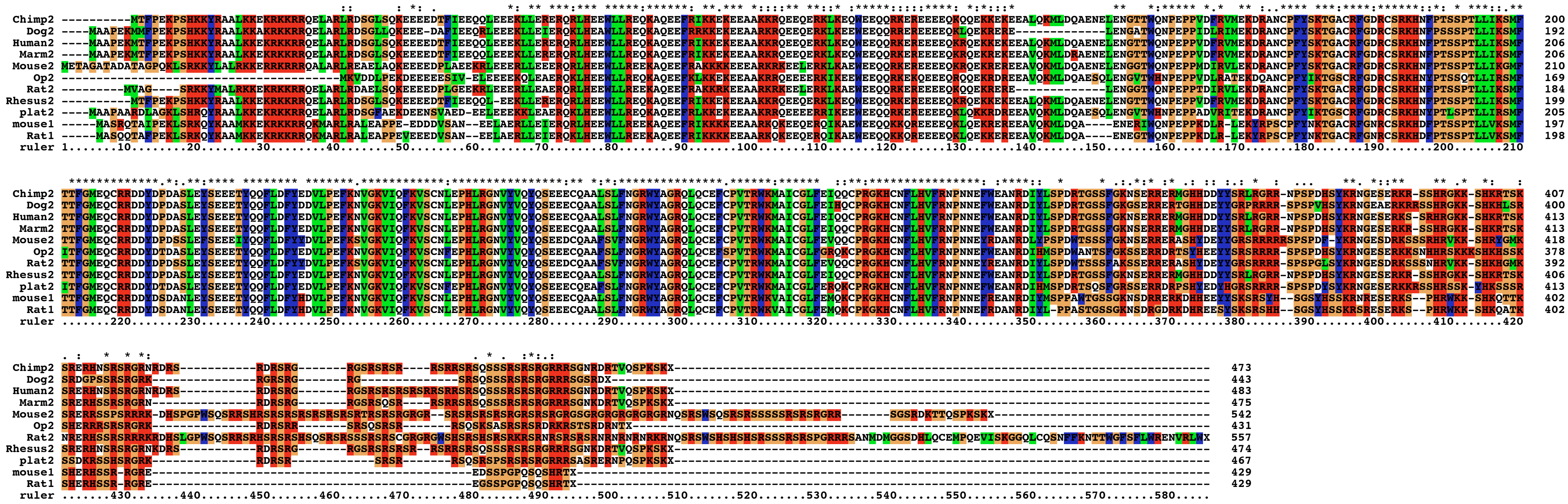
Chimp2
Dog2
Human2
Marm2
Mouse2
Op2
Rat2
Rhesus2
plat2
mouse1
Rat1
ruler
