# Supplementary material for: A Case-by-Case Evolutionary Analysis of Four Imprinted Retrogenes
Source: Evolution. 2011 May;65(5):1413–27. doi: 10.1111/j.1558-5646.2010.01213.x (PMC3107425; doi:10.1111/j.1558-5646.2010.01213.x)
Supplement: Supplementary file 9 [file evo0065-1413-SD9.doc]

**McCole_et_al_2010_Additional Table 6. Positions of positively selected codons in the U2af1-rs1 retrogene lineage – detected using species phylogeny.**

| **Position in**  **alignment^a^** | **P value^b^** |
| --- | --- |
| **38** | 0.714 |
| **63** | 0.997 |
| **154** | 0.607 |
| **206** | 0.774 |
| **313** | 0.705 |
| **355** | 0.998 |
| **384** | 0.895 |
| **385** | 0.974 |
| **388** | 0.524 |
| **480** | 0.996 |
| **485** | 0.620 |
| **491** | 0.948 |
| **493** | 0.832 |

^a^ The position differs from alignment to protein as the alignments file contains sequence gaps – see Table 3 (Main body text).

^b^ Our confidence in each of these sites being positively selected is calculated using the posterior probability and summarised in the P values shown. P values vary from 0.00 (no evidence for belonging in the positively selected category) to 1 (100 % confidence of belonging to the positively selected category).

Greyed out area refers to residues deemed to be false positives due to missing data at that position across all species sampled (gap present in the alignment at that position in at least one of the sampled taxa) and/or poor alignment of the U2af1-rs sequences.
